# MicroRNA-triggered *in situ* programmed assembly of photosensitizers with controlled dimension and accelerated kinetics for precise cancer therapy

**DOI:** 10.1093/nsr/nwaf424

**Published:** 2025-09-29

**Authors:** Jie Sun, Ling-Hong Xiong, Ben Zhong Tang, Xuewen He

**Affiliations:** State Key Laboratory of Bioinspired Interfacial Materials Science, The Key Lab of Health Chemistry and Molecular Diagnosis of Suzhou, College of Chemistry, Chemical Engineering and Materials Science, Soochow University, Suzhou 215123, China; School of Public Health, Suzhou Medical College of Soochow University, Soochow University, Suzhou 215123, China; Guangdong Basic Research Center of Excellence for Aggregate Science, School of Science and Engineering, The Chinese University of Hong Kong, Shenzhen (CUHK-Shenzhen), Shenzhen 518172, China; Department of Chemistry, Hong Kong Branch of Chinese National Engineering Research Centre for Tissue Restoration and Reconstruction, Institute for Advanced Study and Division of Life Science, The Hong Kong University of Science and Technology, Hong Kong 999077, China; State Key Laboratory of Bioinspired Interfacial Materials Science, The Key Lab of Health Chemistry and Molecular Diagnosis of Suzhou, College of Chemistry, Chemical Engineering and Materials Science, Soochow University, Suzhou 215123, China

**Keywords:** microRNA, *in situ* assembly, accelerated, kinetics, dimension control, precise theranostics

## Abstract

The selective *in situ* synthesis and activation of therapeutic agents within tumor cells are critical for enhancing the targetability and preciseness of cancer therapy. Herein, triggered by specific tumor microRNA biomarkers, programmed hybridization-chain reaction (HCR) assemblies of aggregation-induced emission (AIE) photosensitizers were conducted for the *in situ*, rapid and controllable synthesis of anticancer agents in cancer cells. Robust fluorescence and photodynamic activities were thus provoked from scratch for precise cancer therapy. By precisely tuning the DNA valency conjugated to the photosensitizer, controllable assembly of one-dimensional linear-, two-dimensional dendritic-, and three-dimensional spherical-type structures were achieved, in which the two-dimensional assembly showed the greatest gains in turn-on fluorescence and reactive oxygen species (ROS) signals. Notably, the photosensitizer conjugation significantly accelerated the HCR kinetics of hairpin DNAs, thereby facilitating the rapid response to microRNA biomarkers within tumor cells and tissues. This microRNA-responsive kinetics-accelerated and dimension-controllable assembly strategy, provides a new avenue for *in situ* precise cancer theranostics.

## Teaser

*In situ* synthesis and activation of photosensitizer in tumor cell was used for precise tumor therapy via microRNA biomarker triggered controlled assembly.

## INTRODUCTION

Cancer remains one of the most lethal diseases in humans due to its therapeutic resistance, high metastatic potential, and frequent recurrence [1]. Current anticancer strategies, including chemotherapy, radiotherapy and combination therapies, often rely on the targeted delivery and controlled release of therapeutic agents [2,3]. While the unique size and physicochemical properties of nanomaterials can enhance delivery efficiency of therapeutic agents to specific targets, off-target effects during systemic circulation remain a significant challenge [4]. These effects not only reduce the local concentration of therapeutic agents at the tumor site, thereby compromising treatment efficacy, but also increase the risk of systemic toxicity [5]. Therefore, *in situ* synthesis of active therapeutic agents within tumors holds great promise for precise cancer therapy [6,7].

An effective strategy for the *in situ* synthesis of therapeutic agents within cancer cells is programmed assembly [8]. Biological systems inherently rely on programmed assembly processes, such as DNA replication and chromatin organization, where single-stranded DNA is unwound, replicated, and assembled into nucleosomes, ultimately forming higher-order chromatins for storage and transmission of genetic information [9]. Similarly, protein assembly underpins essential cellular functions, as seen in ATP-dependent polymerization of G-actin into F-actin, forming cytoskeletal microfilaments, or the tetramerization of hemoglobin subunits with heme for oxygen transport [10]. Mimicking these endogenous assembly mechanisms for *in situ* synthesis of therapeutic agents is expected to offer enhanced biocompatibility and treatment efficacy [11]. Xiao *et al*. have conducted pioneering work in the *in situ* assembly and synthesis of anticancer drugs for tumor-targeted therapy [12–14]. Moreover, if assembly can be triggered by specific stimuli or pathological changes, such as oncogenic transformation, it ensures precise spatiotemporal control while enabling monitoring of the dynamic cellular statuses through the output of physicochemical signals [15–17]. This approach holds great promise for real-time tracking of cellular processes and operating adaptive disease interventions.

Owing to their pivotal roles in disease onset and progression, biomarkers serve as specific indicators in cellular transition from normal to pathological statuses [18]. Consequently, they provide specific triggering signals for constructing the stimuli-response and *in situ* assembly of systems. Among these, microRNAs (miRNAs) are crucial nucleic acid biomarkers for cancer, with aberrant expression patterns often associated with tumorigenesis [19,20]. For instance, miRNA-21 is markedly upregulated in multiple cancer types, functioning in suppressing tumor-suppressor genes involved in kinase signaling and promoting metastasis and recurrence, making miRNA an ideal initiator for assembly reactions [21]. As an enzyme-free nucleic acid amplification strategy driven by strict base-pairing principles, the hybridization chain reaction (HCR) can be specifically triggered by target nucleic acids to implement programmed assembly, ensuring high selectivity and signal amplification [22,23]. Previous studies have demonstrated the potential of HCR-based amplification strategies for the *in situ* multiplexed bioanalysis at the single-cell/molecule level [24–26]. However, HCR is inherently slow and often takes up tens of minutes or even hours while requiring elevated ionic strength or increased temperature to enhance reaction kinetics, thus limiting its applicability in the real-time monitoring of dynamic fluctuations of nucleic acid variants in living cells [27]. Therefore, employing an HCR strategy for the fast biomarker response and *in situ* assembly of therapeutic agents to intervene in diseases remains challenging.

Compared with small-molecule drugs and agents, nanostructured therapeutic agents via *in situ* assembly within living cells can significantly extend intracellular retention time, thereby enhancing therapeutic efficacy against tumors [28]. As a spatiotemporally controllable and minimally invasive manner, photodynamic therapy (PDT) has attracted considerable attention in the elimination of pathogens [29] and tumor ablation [30] due to its negligible risk of drug resistance. However, current PDT approaches still face many challenges, including poor selectivity, potential off-target cytotoxicity, and low activity under physiological conditions [31]. *In situ* assembly of photosensitizers at specific tumor sites represents a promising strategy to enhance both the selectivity and efficacy of PDT [32]. Photosensitizers with aggregation-induced emission (AIE) properties not only enable ‘turn-on’ and amplified fluorescence signals but also enhance intersystem crossing efficiency and optimize the energy gap between singlet and triplet states, thereby improving reactive oxygen species (ROS) generation capability and PDT outcomes *in situ* [33–35]. Our previous studies demonstrated augmented PDT activities in the alation of tumor and pathogens via nanosurface-bound and enzyme-response AIE photosensitizers [36–43]. However, conventional aggregation of AIE-photosensitizers based on solubility modulation, electrostatic interactions, or hydrophobic effects often result in poor morphological control, leading to random and disordered aggregates, and inconsistent output of fluorescence and PDT properties [39,40,43]. Developing controllable strategies that enable precise assembly of photosensitizers is expected to form ordered structures and output stable and reproducible imaging signals as well as boosting photodynamic activities.

**Figure 1.**
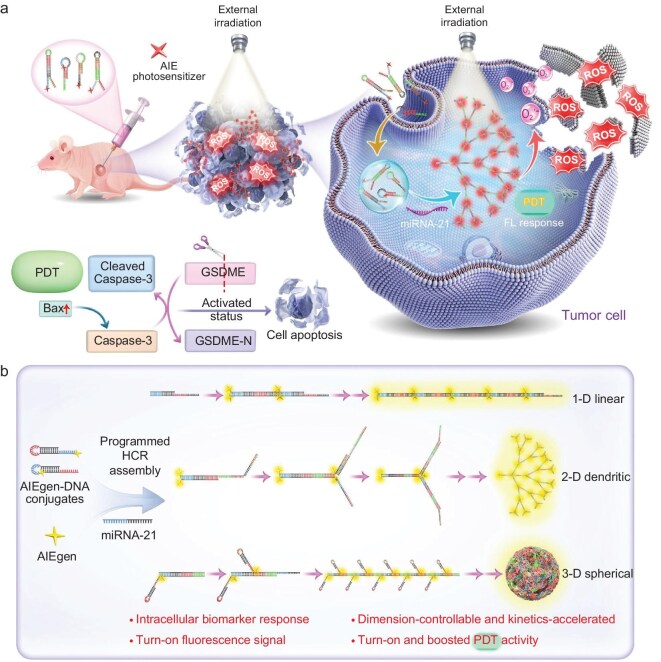
Schematic illustration of the preparation and application of HCR assemblies. (a) After endocytosis, the AIEgen-hairpin DNA conjugates can swiftly assemble upon response to microRNA biomarkers, with the produced fluorescence signal and augmented ROS generation rate applied for cancer cell imaging and discrimination and *in situ* boosted cancer therapy. (b) 1-D liner-, 2-D dendritic- and 3-D spherical-type assemblies were produced via programmed HCR reaction of AIEgen-hairpin DNA conjugates upon the microRNA biomarker triggering.

In this contribution, a novel strategy for the *in situ* synthesis and activation of anticancer agents within cancer cells was developed to precisely interfere with the tumor through specific miRNA biomarker triggered by programmed-assembly of AIE photosensitizers (Fig. 1). A series of AIE luminogens with diverse conjugation tags were designed to covalently link with hairpin DNA of various valencies. Once triggered by a specific miRNA biomarker, HCR assembly is initiated, enabling the controllable assembly of luminogens into one-dimensional (1-D) linear-, two-dimensional (2-D) dendritic-, and three-dimensional (3-D) spherical-type structures, respectively (Fig. S1). A detailed scheme for the step-by-step assembly process of AIEgen-DNA conjugates into 2-D dendritic- and 3-D spherical-type structures is shown in Fig. S2. Among them, the 2-D assembly of AIE photosensitizers showed the greatest amplifications in the generated turn-on fluorescence and ROS signals. Significantly, the conjugation of photosensitizers markedly reduced the melting temperature (*T*_m_) of hairpin DNA, thereby accelerating HCR kinetics and shortening the photosensitizer assembly time to a few minutes. Both *in vitro* and *in vivo* experiments demonstrated the fact that the selective assembly of photosensitizers can be driven by the ultrasensitive miRNA-21 response at picomolar concentrations. The *in situ* provoked fluorescence and PDT activities facilitated sensitive cancer discrimination and image-guided tumor ablation, providing a promising strategy for precise malignancy theranostics.

## RESULTS AND DISCUSSION

### Synthesis and assembly of AIEgen-DNA conjugate

**Figure 2.**
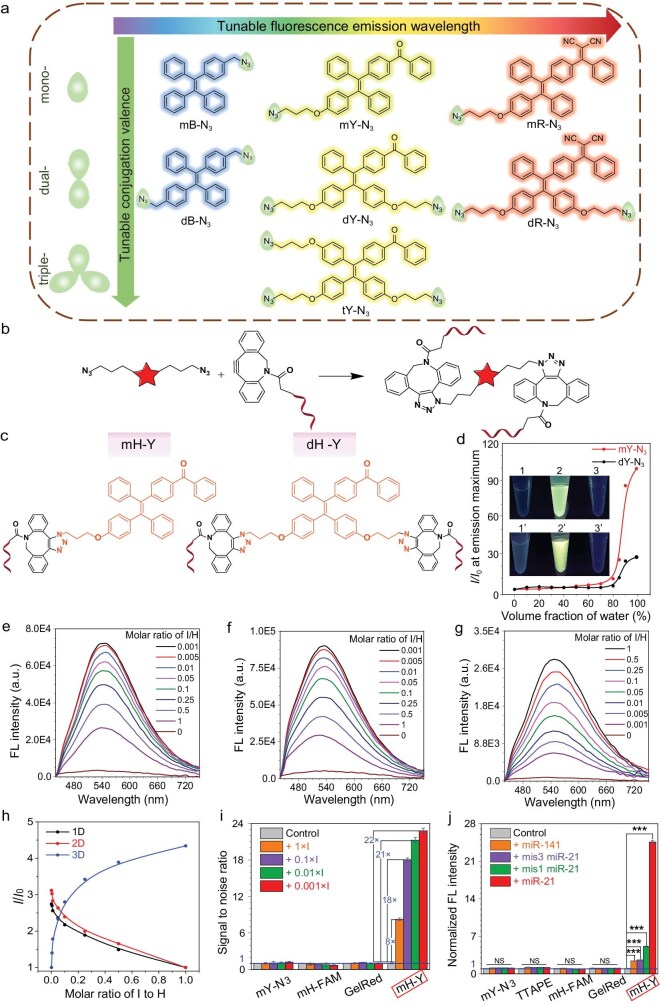
Characterization of the AIE properties of m/dY-N_3_, the signal to noise ratio and selectivity of DNA-programmed AIEgen assembly via HCR reaction. (a) A series of AIEgen molecular structures with different conjugation tags and emission wavelengths. (b) Specific strain-promoted azide-alkyne cycloaddition (SPAAC) reaction with AIEgens and dibenzocyclooctyne (DBCO)-modified DNA strands. (c) Molecular structures of AIEgen-DNA conjugates mH-Y and dH-Y. (d) Plotting of the enhancement ratio at maximum emission of m/dY-N_3_ versus the volume fraction of water in the THF/water mixture. The inset picture was the photo under excitation of a 365 nm laser, in which No.1/1’ was m/dY-N_3_ in 100% THF, No.2/2’ was m/dY-N_3_ in 99% water, and No.3/3’ was m/dH-Y in 100% water. (e–g) Fluorescence spectra of the (e) 1-D, (f) 2-D and (g) 3-D assembly of AIEgen-DNA via HCR reactions with various molar ratios of Initiator to Hairpin (I/H). The excitation wavelength was 365 nm. (h) Plotting of the fluorescence intensity at the maximum of produced 1-D, 2-D and 3-D assemblies versus the molar ratio of I/H. The HCR reaction time was 30 min for all groups. (i) Comparison of the signal-to-noise ratio of mY-N_3_, H_1_ and H_2_ modified FAM, GelRed and mH-Y before and after adding 1-fold, 0.1-fold, 0.01-fold, 0.001-fold Initiator. (j) Selectivity comparison of mY-N_3_, TTAPE which can recognize nucleic acid, H_1_ and H_2_ modified FAM, GelRed and mH-Y with non-complementary target (miR-141), target RNA with one or three mismatched bases, and target miRNA-21 before and after adding 0.001-fold Initiator. ***P < 0.01 versus control.

We initially designed and synthesized AIE luminogens (AIEgens) with mono-, dual-, and triple-azido (abbreviated as ‘m’, ‘d’, or ‘t’, respectively) tagging groups and different emission colors (B: Blue. Y: Yellow. R: Red), termed mB-N_3_, dB-N_3_, mY-N_3_, dY-N_3_, tY-N_3_, mR-N_3_, and dR-N_3_, respectively (Fig. 2a). The synthesis procedures are described in Fig. S3 in detail with molecular structures characterized by ^1^H and ^13^C nuclear magnetic resonance (NMR) and high-resolution mass spectroscopy (HRMS) in the Supplementary Information. These compounds exhibited excellent AIE characteristics, demonstrating negligible fluorescence in the solvent tetrahydrofuran (THF). Whereas the fluorescence (FL) intensified significantly upon increasing the proportion of water, a poor solvent, as illustrated in Fig. 2d and Fig. S4, with FL intensity increased by 99.5-, 27.6-, and 34.5-fold for mY-N_3_, dY-N_3_, and tY-N_3_, respectively, at 99% water fraction, highlighting their potential for turn-on biosensing and imaging.

Subsequently, we covalently linked these AIEgens with dibenzocyclooctyne (DBCO)-modified DNA strands through a strain-promoted azide-alkyne cycloaddition (SPAAC) reaction under mild conditions (Fig. 2b). As depicted in Fig. 2c, mY-N_3_ with mono-azido group was designed to conjugate with one DNA strand for preparation of 1-D and 2-D hybridization chain reaction (HCR) assemblies, while dY-N_3_, featuring dual-azido groups, linked with two DNA strands, was designed for preparing 3-D HCR assemblies. Meanwhile, tY-N_3_, with triple-azido groups, linked with three DNA strands, was also designed for 3-D HCR assemblies. Following separation and purification via high-performance liquid chromatography (HPLC) equipped with a reverse phase C18 column, we obtained the AIEgen-DNA conjugates with one AIE molecule covalently linked with one, two, or three hairpin DNA strands (abbreviated as H), termed as mH-Y, dH-Y, tH-Y, mH-R, dH-R, respectively. UV-visible spectra confirmed the successful conjugation of AIEgen with DNA, as the conjugates exhibited a characteristic DNA absorption peak at 260 nm and an AIEgen absorption peak at 365 nm. The measured molar extinction coefficients of mY-N_3_, dY-N_3_, and tY-N_3_ confirmed the 1 : 1, 2 : 1, and 3 : 1 molar ratios of DNA to AIEgen in mH-Y, dH-Y, and tH-Y, respectively (Fig. S5). Under non-denaturing polyacrylamide gel electrophoresis (PAGE) analysis, the mobility rate of AIEgen-DNA conjugates was significantly slower compared to their free DNA counterparts (Figs S6–S8). In the reverse phase column in HPLC, the retention time of hairpin DNA increased from 2.6 min to 12.3 min (or 7.2 min or 6.1 min) after conjugating AIEgens with the mono- (or dual- or triple-) strand hairpin DNA, respectively (Fig. S9). Mass spectra data showed that all the AIEgen-DNA conjugates for 1-D, 2-D, and 3-D assemblies were perfectly consistent with the predicted results, further confirming the successful connection of AIEgen to hairpin DNA with a defined conjugation valence (Fig. S10).

To produce 1-D linear, 2-D dendritic, and 3-D spherical-type assemblies, the AIEgen-DNA conjugates underwent an HCR reaction triggered by a specific DNA initiator (herein is the sequence of miRNA-21). As shown in Fig. 2e–h and Fig. S11, accompanied by the progress of the HCR reaction, AIEgen-conjugated hairpin DNA assembled to linear or dendritic polymers, resulting in the strong restriction of intramolecular motion of the conjugated AIEgens in the assemblies and markedly enhanced fluorescence signals. As the molar ratio of initiator DNA (abbreviated as ‘I’) to hairpin DNA (abbreviated as ‘H’) decreased, larger aggregates formed in both 1-D (mH_1_-Y + mH_2_-Y, abbreviated as mH_1-2_-Y) and 2-D (mH_3_-Y + mH_4_-Y + mH_5_-Y + mH_6_-Y, abbreviated as mH_3-6_-Y) assemblies. Notably, the fluorescence signal of the 2-D aggregates increased by 10.1-fold compared to that without initiator (I) input. In contrast, when increasing the I/H molar ratio, the fluorescence intensity of produced 3-D assemblies after HCR reaction of [d(H_7_/H_7_)-Y + d(H_8_/H_8_)-Y] or [t(H_7_/H_7_/H_7_)-Y + H_8_] showed an intensifying trend. This result is likely due to the increased steric hindrance and restricted HCR reaction rate since dual hairpin (H_7_ or H_8_) strands or triple hairpin (H_7_) was designed to link with one AIEgen in the 3-D HCR mode. The probability for initiator DNA to open the dual H_7_ strands conjugated on one AIEgen is hypothesized to be higher than that for the one-by-one conjugation mode in the 1-D and 2-D HCR reaction, which may inhibit the formation of large assemblies in the 3-D HCR reaction. Moreover, when one H_7_ complementarily paired with H_8_, it became more difficult for the adjacent H_7_ to hybridize with H_8_ that conjugated on another AIEgen, leading to a much lower HCR reaction efficiency. When increasing the molar ratio of initiator (I), the conversion rate of the HCR reaction can be improved with more AIEgen-conjugated hairpin DNA being assembled, leading to an intensified fluorescence signal. For further investigation, we designed another hairpin DNA strand, H_9_, which cannot hybridize with either H_7_ or H_8_ via complementary base pairing. We first conjugated dual-azide grouped AIEgens (dY-N_3_) with either one H_7_ or H_8_ strand, and then saturated with a H_9_ strand, generating a type of heterobivalent conjugate, as d(H_7_/H_9_)-Y and d(H_8_/H_9_)-Y. Similar to the above 1-D and 2-D HCR assemblies, it was found that the fluorescence intensity in the HCR assembly of d(H_7_/H_9_)-Y + d(H_8_/H_9_)-Y exhibited a continuously decreasing trend as the miRNA-21 initiator ratio increased (Fig. S11). Thus, the reason for the positive enhancement in the fluorescence intensity of the 3-D HCR assemblies along with the increase in I/H molar ratio can be attributed to the strong steric hindrance effect from the dual or triple hairpins that conjugated to a single AIEgen that only allow a limited degree of assembly at a low ratio of Initiator and a greater amount of Initiator is needed to complete the HCR assembly of those AIEgen-hairpin DNA and finally induce its aggregated fluorescence.

Given the excellent turn-on property of the fluorescence in response to miRNA-21 biomarkers, we next examined the fluorescence signal-to-noise ratio by comparison with four different groups: AIEgen-conjugated hairpin DNA (mH-Y), naked AIEgen (mY-N_3_), hairpin DNA modified with fluorescein amidite (FAM) fluorophore (mH-FAM), and the commercial nucleic acid staining dye, GelRed, under the same fluorophore concentration. As shown in Fig. 2i and Fig. S13, upon sequential addition of varying proportions of Initiator (I), the fluorescence signals of mY-N_3_, mH_1-2_-FAM, and GelRed remained nearly unchanged compared to their respective controls without I input. However, the AIEgen-conjugated DNA, mH_1-2_-Y, exhibited a significantly enhanced fluorescence signal as the proportion of added Initiator (I) decreased. Notably, at an Initiator concentration of 0.001-fold, the fluorescence signal of mH-Y was amplified by 22-fold compared to mH-Y alone. This remarkable fluorescence signal-to-noise ratio highlights the superior sensitivity of mH-Y for targeted miRNA response. Next, the selectivity of the system in recognition of target miRNA-21 was evaluated. In addition to the four previously mentioned groups, we introduced another AIEgen, TTAPE (structure shown in Fig. S14a), which can bind DNA strands and emit fluorescence signals [44,45], as a control. As shown in Fig. 2j and Fig. S14, regardless of whether 0.001-fold non-complementary target (miR-141), target RNA with one or three mismatched bases, or the perfectly matched target miRNA-21 (I) was added, the fluorescence signals of mY-N_3_, TTAPE, mH-FAM, and GelRed showed no significant increase compared to their respective intrinsic fluorescence. However, upon the addition of target miRNA-21, mH-Y efficiently initiated the HCR reaction, leading to the aggregation of AIEgen and a significant enhancement in fluorescence. Further, negligible fluorescence enhancement was observed when using non-target miRNA-141 or target miRNA-21 with a three-base mismatch as the initiator in the HCR reaction of mH-Y. Even with a single-base mismatch, the fluorescence enhancement was almost inhibited, demonstrating the excellent selectivity of the designed HCR reaction.

### Accelerated kinetics of HCR assembly

**Figure 3.**
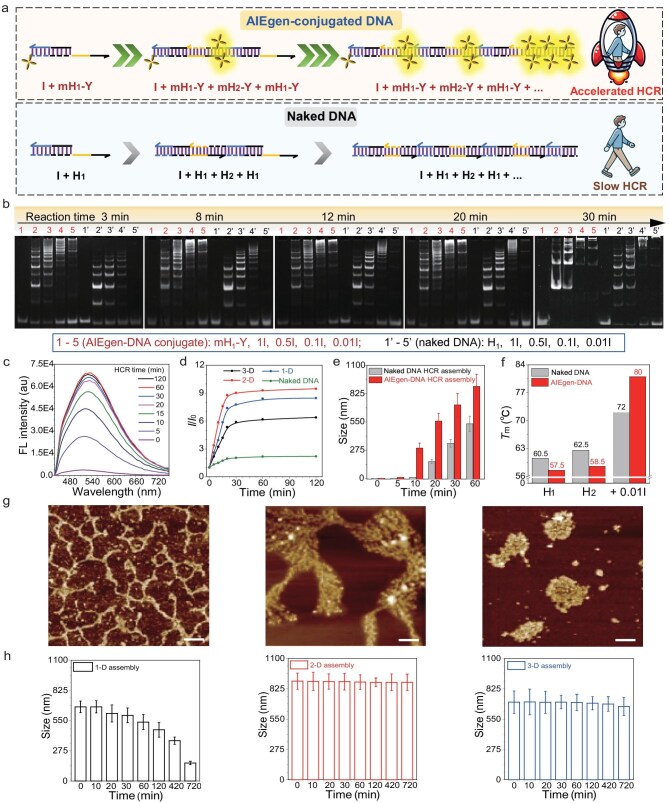
Characterization of the kinetics of DNA-programmed AIEgen assembly via HCR reaction and morphology characterization of various dimensional HCR assemblies. (a) Schematic comparison of HCR reaction kinetics between AIEgen conjugated DNA and naked DNA. (b) Monitoring of the HCR reaction process via non-denaturing PAGE gel electrophoresis of the produced 1-D assemblies from free hairpin DNA or AIEgen-conjugated hairpin DNA along with the reaction time from 3 min to 30 min in 0.4× PBS. (c) Fluorescence spectra of the 2-D assembly of AIEgen-DNA via HCR reactions with various times excited by a 365 nm laser [mH-Y] = 2 × 10^−6^ M. (d) Plotting of fluorescence intensity at the maximum produced 1-D, 2-D and 3-D assemblies versus various times. (e) Monitoring of the HCR reaction process via dynamic light scattering measurements of the hydrodynamic sizes of the produced 1-D assemblies from free hairpin DNA or AIEgen-conjugated hairpin DNA along with the reaction time from 3 min to 60 min. (f) Nucleic acid melting temperature testing of H_1_, H_2_, and 1-D HCR assemblies with or without AIEgen conjugation via UV-Vis spectra. (g) Morphological characterization of the controlled assembly AIEgen-DNA for linear 1-D, dendritic 2-D, and spherical 3-D structures via AFM imaging. The 0.001-fold (1-D and 2-D) and 0.1-fold (3-D) Initiator was used as the trigger for the HCR reaction. All scale bars are 500 nm. (h) Dynamic light scattering measurements of the hydrodynamic sizes of the produced 1-D, 2-D, and 3-D assemblies after adding 50 U L^−1^ DNase I with incubation times from 0 min to 720 min.

It is interesting that the HCR reaction rate of AIEgen-DNA conjugates was remarkably faster than their naked DNA counterparts (Fig. 3a). As shown in Fig. 3b, the 1-D HCR reactions using mH_1_-Y and mH_2_-Y were set up at varying I/H molar ratios of 1, 0.5, 0.1, and 0.01. After HCR reaction for 3, 8, 12, 20, and 30 min, the two sets of samples were simultaneously characterized via PAGE electrophoresis. Direct imaging results without GelRed staining revealed that as the HCR progressed, the fluorescence in the AIEgen-DNA conjugates intensified and became brighter with decreasing molar ratios of I/H (Fig. S15), aligning with the above fluorescence spectra results. After GelRed staining, typical HCR ladder bands appeared in both groups while located at different positions. The group of mH_1-2_-Y conjugates showed more pronounced bands near the well, with far fewer unreacted hairpins remaining compared to the naked hairpin DNA (H_1_ + H_2_) group, suggesting a more complete reaction and the formation of larger assembly products. At a 0.01 molar ratio of I/H, the conjugates mH_1-2_-Y formed a large amount of assemblies at the top of the lane after 20 min of HCR, whereas the band was much lower in naked hairpin DNA (H_1_ + H_2_). After 30 min HCR, the mH_1-2_-Y group thoroughly reacted, with no remaining mH-Y observed at the bottom of the lane. In contrast, a substantial amount of unreacted DNA was still present in the naked hairpin DNA group (H_1_ + H_2_). Figures S16–S18 also showed accelerated HCR rates for 2-D (mH_3-6_-Y) and 3-D [d(H_7_/H_7_)-Y + d(H_8_/H_8_)-Y] or [t(H_7_/H_7_/H_7_)-Y + H_8_] conjugates versus naked hairpin DNA. These results demonstrate the notably faster HCR rates for AIEgen-conjugated hairpin DNA compared to their naked hairpin DNA counterparts.

Next, the HCR reaction was monitored via fluorescence spectra (Fig. 3c and Fig. S19). The fluorescence emission of mY-N_3_ was employed for the direct indication of HCR production in the AIEgen-conjugated hairpin DNA group, and the HCR assemblies of naked hairpin DNA were stained with GelRed for fluorescence measurement. It can be observed that the fluorescence intensified rapidly in the mH_1-2_-Y group and saturated at ∼20 min. It was much faster than that in the naked hairpin DNA group (H_1_ + H_2_), whose HCR reaction did not complete even after 2 h (Fig. 3d). The assembly rate (20 min) made our AIEgen-conjugated hairpin DNA system one of the fastest HCR systems reported to date (Table S1), facilitating the efficient and rapid target response in living cells. Additionally, dynamic light scattering (DLS) was employed to assess the rate of the HCR reaction. As shown in Fig. 3e and Fig. S20, the particle sizes of the HCR assemblies formed by the mH_1-2_-Y group or the naked hairpin DNA group (H_1_ + H_2_) were monitored at 0, 5, 10, 20, 30, and 60 min, respectively. The results indicated that, at the same reaction time, the assembly particle size of the mH_1-2_-Y group was always larger than that of the naked hairpin DNA group (H_1_ + H_2_). For instance, at 10 min, the particle size of the mH_1-2_-Y group was 295.3 nm, while the naked hairpin DNA group (H_1_ + H_2_) showed 9.8 nm. At 30 min, the particle size of the mH_1-2_-Y group increased to 715.1 nm, whereas it only grew to 342 nm in the naked hairpin DNA group (H_1_ + H_2_). This further demonstrates the much faster HCR reaction rate after conjugation of AIEgen to hairpin DNA.

In addition to these kinetic experiments, the accelerated HCR was further validated from a thermodynamic perspective. As shown in Fig. 3f, and Figs S21 and S22, the *T*_m_ values of AIEgen-conjugated hairpin DNA were lower than those of naked hairpin DNA. For instance, the *T*_m_ values of H_1_ and H_2_ were 60.5 and 62.5°C, respectively, whereas those of mH_1_-Y and mH_2_-Y were 57.5°C and 58.5°C, a decrease of 3.0°C and 4.0°C, respectively. Similarly, the *T*_m_ values of mH_3_-Y, mH_4_-Y, mH_5_-Y, and mH_6_-Y decreased by 3.0°C, 2.0°C, 3.0°C, and 2.5°C, respectively, compared to their naked hairpin DNA counterparts. These results indicated that under identical conditions, AIEgen-conjugated hairpin DNA was more prone to open its hairpin structure compared to naked hairpin DNA, thereby facilitating HCR more promptly. Moreover, the *T*_m_ values of the HCR assemblies formed by AIEgen-conjugated hairpin DNA were significantly higher than those of naked HCR assemblies. For example, for 1-D mH_1-2_-Y and 2-D mH_3-6_-Y assemblies with an I/H molar ratio of 0.01, the *T*_m_ values were 8.0°C and 7.5°C higher than that of the naked DNA composed HCR assemblies, respectively, suggesting that the HCR assemblies become more stable after the hairpin DNA is conjugated by AIEgens. In other words, AIEgen-conjugated hairpin DNA was more likely to undergo HCR upon target miRNA response and form more stable assemblies compared to naked hairpin DNA. These results demonstrated that our AIEgen-conjugated DNA probes can facilitate a much more rapid HCR, indicating great potential for fast miRNA biomarker response.

### Dimension-controllable HCR assembly

Through fluorescence spectra, we successfully demonstrated that the AIEgen-conjugated hairpin DNA underwent a specific response to target miRNA-21, triggering HCR-mediated aggregation and intensifying the fluorescence emission. The next step involved investigating whether they formed the anticipated 1-D, 2-D, and 3-D morphologies. Initially, AIEgen-DNA conjugates were prepared in phosphate buffer and pre-mixed separately to facilitate the formation of distinct hierarchical structures: 1-D (mH_1-2_-Y), 2-D (mH_3-6_-Y), and 3-D [d(H_7_/H_7_)-Y + d(H_8_/H_8_)-Y] or [t(H_7_/H_7_/H_7_)-Y + H_8_]. The I/H molar ratios of 0.001, 0.001, and 0.1 were selected for characterizing the morphology of the 1-D, 2-D, and 3-D HCR assemblies, respectively. As shown in Fig. 3g, and Figs S23 and S24, the atomic force microscopy (AFM) images matched our design: the 1-D HCR assemblies appeared as linear chains, the 2-D HCR formed dendritic-like assemblies, and the 3-D HCR exhibited spherical structures. We then employed DLS to measure the particle sizes of the assemblies (Fig. S25). The hydrated particle size was ∼630 nm for the 1-D products, ∼1000 nm for the 2-D products which had the largest size, and ∼750 nm for the 3-D products, which are consistent with the above AFM results. Scanning electron microscopy (SEM) images of the 1-D, 2-D, and 3-D products further confirmed the successful assembly into linear, dendritic, and spherical structures (Fig. S26). Additionally, elemental analysis of the 3-D assemblies verified the presence of carbon, nitrogen, oxygen, and phosphorus—four elements contained in the assemblies (Fig. S27). Fluorescence imaging directly verified the formation of 1-D, 2-D, and 3-D assemblies as they emitted bright yellow fluorescence (Fig. S28).

Next, the structural stability of the HCR assemblies was further evaluated. The HCR reaction was initiated using a 0.001-fold initiator for the 1-D and 2-D assemblies and a 0.1-fold initiator for the 3-D assemblies, and their hydrodynamic diameters were monitored over time using DLS after incubation with 50 U L^−1^ DNase I. As shown in Fig. 3h, shrinking size from 675 nm to 164 nm appeared in the 1-D assemblies after incubation with 50 U L^−1^ DNase I, likely due to their less compact structure and reduced resistance to enzymatic degradation. In contrast, the particle sizes of the 2-D and 3-D assemblies remained unchanged even after 12 h of incubation with DNase I, demonstrating their exceptional stability. PAGE electrophoresis results also showed negligible changes in the 2-D and 3-D assemblies after DNase I treatment, further confirming their structural stability (Fig. S29).

### miRNA response in living cells and intracellular HCR characterization

**Figure 4.**
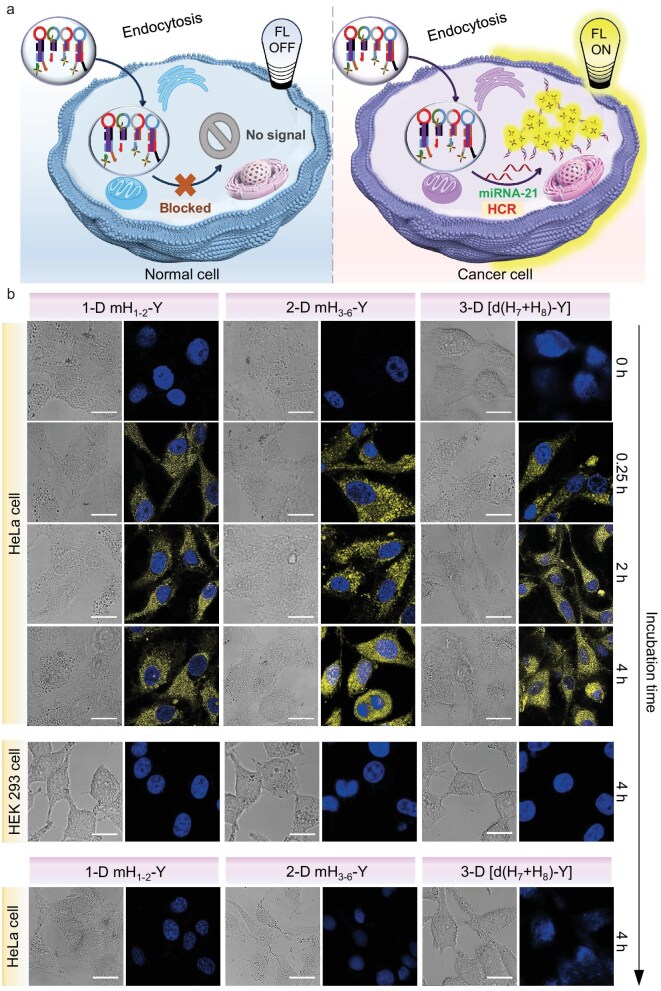
Confocal laser scanning imaging of the formation of 1-D, 2-D, and 3-D assemblies in living cells. (a) Schematic representation of the action of HCR assembly in cells. (b) Confocal laser scanning imaging of living cells used to monitor the assembly process of 1-D, 2-D, and 3-D AIEgen-DNA assemblies along with varying co-incubation time. The cancerous HeLa cells were fed by mH_1-2_-Y, mH_3-6_-Y, and [d(H_7_/H_7_)-Y + d(H_8_/H_8_)-Y] to produce 1-D, 2-D, and 3-D assemblies, respectively. Normal HEK cells that coincubated with AIEgen-DNA conjugates for 4 h was set as the control group. Confocal laser scanning imaging of HeLa cells that were fed by mH_1-2_-Y, mH_3-6_-Y, and [d(H_7_/H_7_)-Y + d(H_8_/H_8_)-Y] was set as a negative control after coincubation for 4 h. The cells were stained by Hoechst nuclear dye. Yellow channel for m/dY-N_3_: excitation 405 nm, emission 530–600 nm. Blue channel for Hoechst: excitation 405 nm, emission 430–470 nm. All scale bars are 20 ${\mathrm{\mu }}$m.

The AIEgen-DNA conjugated probes were next applied for *in situ* miRNA-21 response in live cells (Fig. 4a). To enhance the cellular uptake of the DNA probes, three sets of conjugates—1-D (mH_1-2_-Y), 2-D (mH_3-6_-Y), and 3-D [d(H_7_/H_7_)-Y + d(H_8_/H_8_)-Y]—were transfected into HeLa cells using Lipofectamine. As shown in Fig. 4b, after just 15 min of co-incubation, bright yellow fluorescence was observed in HeLa cells for all three groups, indicating the miRNA-21 response in HeLa cells and initiation of the HCR reaction of AIEgen-hairpin DNA. Co-staining Hoechst nuclear dye revealed that the assemblies were located in the cytoplasm where the conjugates can contact with miRNA biomarkers. Continued co-incubation for 2 h and 4 h still displayed bright yellow fluorescence signals within HeLa cells, as observed under confocal laser scanning imaging, indicating the excellent photostability of the assemblies, and providing a broad time-window for potential therapy operation. In contrast, in human embryonic kidney cells (HEK293), no fluorescence signal was detected even after 4 h co-incubation, suggesting the specificity of the HCR assembly of AIEgen-DNA conjugated probes triggered by the tumor biomarker miRNA-21 and capability to accurately distinguish between tumor and normal cells. Further, when only partial conjugates of 1-D (e.g. mH_1_-Y), 2-D (e.g. mH_3_-Y + mH_6_-Y), and 3-D (e.g. dH_7_-Y) were transfected into HeLa cells, no fluorescence activation was observed even after 4 h of co-incubation (Fig. 4b), highlighting the extremely low fluorescence background and strong turn-on signal which occurred only upon specific miRNA biomarker response. Additionally, a concentration-dependent positive response manner of fluorescence signal was found in the cancer cell imaging in the range of 2 to 8 μM mH_3-6_-Y conjugates for *in situ* 2-D assembly (Fig. S30).

HeLa cells treated with mH_3-6_-Y conjugates were then lysed for AFM imaging. It revealed the formation of 2-D dendritic, network-like structures (Fig. S31), validating that the occurrence of HCR assembly within living cells. In addition to HeLa cells, the mH_3-6_-Y conjugates were also delivered to three other cancer cell lines with abnormally high miRNA-21 expression (MCF-7, B16, and HepG-2), in which bright yellow fluorescence can all be observed (Fig. S32). These results collectively demonstrate the capability of AIEgen-conjugated hairpin DNA to perform ultra-sensitive *in situ* response to low-abundance miRNA biomarkers within living cells, with virtually zero background and high fluorescence signal. This allows for the discrimination of cancerous cells from normal cells, laying a strong foundation for precise image-guided cancer therapy.

### PDT activity characterization and image-guided cancer therapy

**Figure 5.**
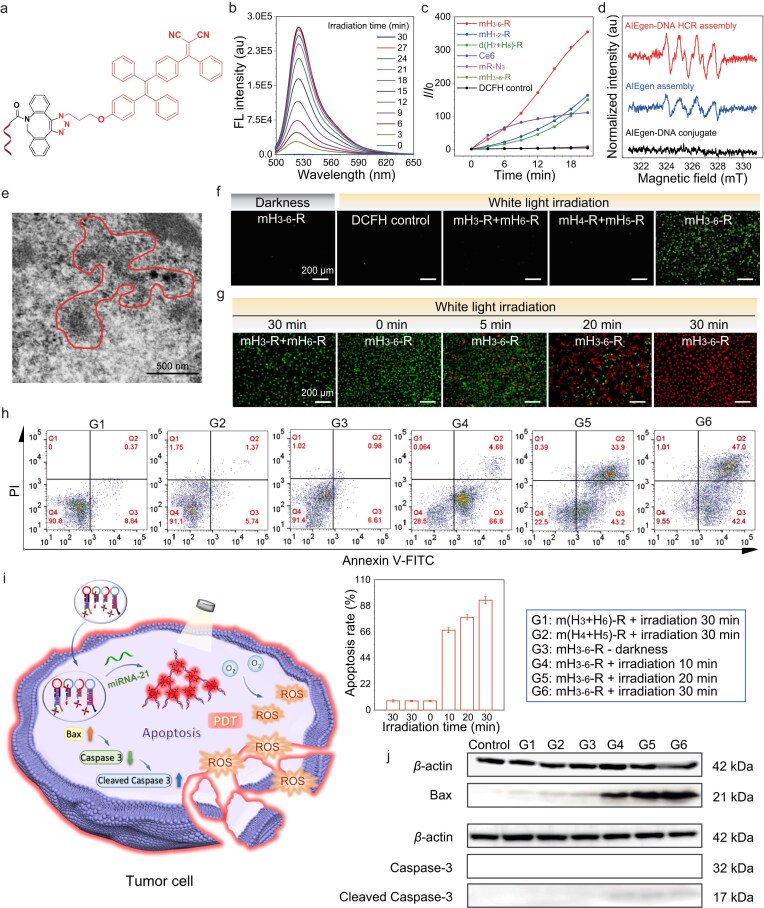
Photodynamic killing of cancer cells via targeted microRNA-triggered AIEgen-DNA assembly. (a) The molecular structure of mH-R. (b) ROS generation capability tests of the formed 2-D–type assemblies via Initiator + mH_3-6_-R under white light irradiation. DCFH was used as an indicator which can emit green-color fluorescence under the activation of generated ROS. The white-light power density was 2 mW cm^−2^. (c) Plotting of the fluorescence intensity of DCFH indicator after mixing with various AIEgen-DNA conjugates and their dimension-controllable assemblies versus irradiation time under white light. (d) ESR spectroscopy of the formed 2-D–type assemblies via mH_3-6_-R with or without Initiator and AIEgen-aggregate with similar concentration after white light irradiation, respectively. (e) HeLa cells treated with mH_3-6_-R for 4 h. The 2-D–type assembly morphology of HeLa cells was detected by TEM imaging. (f) ROS generation characterization in HeLa cells via fluorescence imaging of DCFH indicators after incubating with mH_3-6_-R and treatment by white light irradiation or darkness. The green color emission was from the ROS-activated DCFH. (g) Live/dead cell staining by Calcein AM/PI along with white light irradiation time. All scale bars are 200${\mathrm{\ \mu }}$m. (h) Flow cytometric tests of HeLa cells via detection of stained Annexin V-FITC and PI after treatment with mH_3-6_-R and white light irradiation. The statistic ratio of the number of apoptosis cells indicated by the FITC and PI signal is presented as a histogram. (i) Schematic representation of the apoptosis pathway of HeLa cells under 2-D assembly therapy. (j) Bax, Caspase-3, and cleaved Caspase-3 expression levels of HeLa cells with mH_3-6_-R after white light irradiation as detected by western blotting. $\beta $-actin was used as a control for protein loading.

Initially, we designed and synthesized AIE photosensitizers mR-N_3_ and dR-N_3_, which also displayed unique AIE fluorescence. As shown in Fig. S4d and e, the fluorescence intensity ratios for mR-N_3_ and dR-N_3_ increased by 310.7-fold and 27.6-fold, respectively, at 99% water fraction. To gauge the ROS generation efficiency of mR-N_3_ and dR-N_3_, we utilized 2′,7′-dichlorofluorescin diacetate (DCFH) as an indicator, which emits fluorescence in a turn-on fashion when triggered by ROS. As depicted in Fig. S33, during 10 min white light irradiation (∼2 mW cm^−2^), the emission intensity of the DCFH indicator gradually increased when mixed with aggregated mR-N_3_ or dR-N_3_, achieving enhancements of 5.5-fold and 3.6-fold, respectively, compared to the DCFH control. This underscores the potential of mR-N_3_ and dR-N_3_ serving as photodynamic agents. Thus, we covalently attached these AIE photosensitizers to DBCO-modified hairpin strands (H_1_–H_8_) through the SPAAC reaction, as shown in Fig. 5a. Subsequently, the conjugates specifically respond to miRNA-21, as previously described, to initiate the HCR reaction and form 1-D (mH_1_-R + mH_2_-R, abbreviated as mH_1-2_-R), 2-D (mH_3_-R + mH_4_-R + mH_5_-R + mH_6_-R, abbreviated as mH_3-6_-R), and 3-D [d(H_7_/H_7_)-R + d(H_8_/H_8_)-R] assembled structures, thereby triggering the aggregation of AIE photosensitizers and consequently enhancing their ROS generation capabilities. As illustrated in Fig. 5b, c and Fig. S34, the fluorescence intensity of DCFH in these aggregates remarkably escalated over 30 min white light irradiation with the rate of ROS production accelerating significantly, displaying 61.9-fold, 78.3-fold, and 57.9-fold increases, respectively, compared to the DCFH control. Among these, the 2-D assembly exhibited the strongest ROS-generating capabilities—outperforming commercial photosensitizers like Chlorin e6 (Ce6) by up to 3.9-fold. The ROS production efficiency of non-aggregated conjugates was significantly inhibited; only when miRNA triggers the formation of HCR assemblies can there be a significant enhancement in ROS production capability. Additionally, under white light irradiation in the presence of the superoxide anion (O_2_^•−^) indicator dihydrorhodamine 123 (DHR 123), there was a notable fluorescence enhancement in the 2-D assemblies, which remained the most pronounced with an increase in fluorescence intensity of 36.3-fold (Fig. S35). Subsequent electron spin resonance (ESR) tests, presented in Fig. 5d, after white light irradiation, both the 2-D assembly and the aggregated mR-N_3_ exhibited ESR characteristic peaks representing O_2_^•‒^ in the presence of the free radical scavenger 5,5-dimethyl-1-pyroline N-oxide (DMPO) with a much stronger signal for the 2-D assembly. Conversely, non-assembled mH_3-6_-R conjugates showed negligible radical signals.

Importantly, the assemblies formed by AIE photosensitizer-DNA conjugates demonstrated significantly stronger ROS-producing capabilities compared to the aggregated states of mR-N_3_ and dR-N_3_ at the same concentration of AIE photosensitizers. It is speculated that, in addition to the higher degree of aggregation of AIE photosensitizers in the HCR assemblies, the presence of azide groups in mR-N_3_ and dR-N_3_ may contribute to ROS quenching. Consequently, we designed and synthesized mR-Cl, an AIE photosensitizer with a chlorine atom replacing the azide group. As shown in Fig. S36, upon white light irradiation, the emission intensity of DCFH mixed with the aggregated state of mR-Cl significantly increased, showing a 7.5-fold enhancement over the aggregated state of mR-N_3_. This confirmed the restoration of ROS generation capability. In contrast, mR-Cl and mR-N_3_ in dispersed states, along with the standalone DCFH control, exhibited negligible green fluorescence emission. As depicted in Fig. S37, under identical concentrations of AIE photosensitizers, the 2-D assembly continued to show superior ROS generation, with rates significantly faster than the other groups. Particularly, the ROS production capability of the 2-D assembly was 1.7-fold that of the aggregated mR-Cl, 57.8-fold that of the aggregated mR-N_3_, and 259.4-fold that of the mH_3-6_-R conjugates. These results collectively indicate the assemblies formed by AIE photosensitizer-DNA conjugates have strong capacity to produce ROS, holding great potential in photodynamic tumor therapy. Further, we examined the ROS production efficiency of the 2-D assemblies after one- and two-rounds of 30 min white light irradiation. As shown in Fig. S38, the fluorescence of the DCFH indicator intensified by 1168-fold (after first-round irradiation) and 1095-fold (after second-round irradiation), showing almost the same efficiency in ROS generation. In addition, AFM images confirmed that the 2-D assemblies kept the similar branched assembled morphology before and after white light irradiation (Fig. S38f and g). These findings verified that the AIE photosensitizer-DNA assemblies exhibit excellent structural stability and sustained ROS generation ability after one cycle of PDT.

As the strongest ROS generation capability, we selected the 2-D AIE photosensitizer-DNA conjugates for PDT in tumor cells. To verify whether the mH_3-6_-R conjugates can undergo assembly within tumor cells, treated HeLa cells exhibiting fluorescence were centrifuged and collected. Following fixation, the cells were sectioned for transmission electron microscope (TEM) imaging. A two-dimensional dendritic structure was successfully captured that is similar to the *in vitro* assembly, as outlined by the red dashed line in Fig. 5e. Next, the generation of ROS by the 2-D assembly within living cells was assessed. As illustrated in Fig. 5f, bright green fluorescence of DCFH was only detected when the mH_3-6_-R conjugates underwent HCR assembly upon specific response to miRNA-21 and in the presence of white light irradiation within HeLa cells. In the absence of irradiation or when assembly did not occur, no cellular fluorescence was observed, which was similar to that of standalone cells and the DCFH control. Subsequently, we explored the photodynamic activity of the assemblies of AIE photosensitizer-DNA conjugates in tumor cells. Live/dead cell staining and flow cytometry using Calcein acetoxymethyl ester (Calcein AM)/propidium iodide (PI) were performed to assess cell viability. As depicted in Fig. 5g and Figs S39–S42, with extending white light irradiation time and increasing the concentration of mH_3-6_-R conjugates, HeLa cells exhibited an intensifying red fluorescence signal in the PI channel, indicating a rising proportion of apoptotic cells. In contrast, cells retained bright green fluorescence, indicating good cellular viability without light exposure or in the absence of conjugate treatment, suggesting negligible phototoxicity and dark toxicity of the AIEgen-DNA conjugates. The photodynamic cytotoxicity of the conjugates was further evaluated using MTT assay. Upon specific response to miRNA-21 and the consequent HCR assembly, a concentration and irradiation time-dependent phototoxicity was observed. At a concentration of 5 μM of mH_3-6_-R and 30 min white light irradiation, a 98% kill rate against tumor cells was achieved, indicating a strong photodynamic activity (Figs S43 and S44). In contrast, when treating HEK-293 cells with mH_3-6_-R conjugates, under both irradiated and non-irradiated conditions, cytotoxicities were negligible, indicating that ROS only generated in miRNA-21 target overexpressed cancer cells thus enabling specific cancer treatments (Figs S45–S48).

Additionally, the apparent apoptosis of HeLa cells, which increased with prolonged white light irradiation, was confirmed using an Annexin V-fluorescein isothiocyanate (FITC) apoptosis detection kit through flow cytometry (Fig. 5h), consistent with the findings from Calcein AM/PI staining and the 3-(4,5-dimethylthiazol-2-yl)-2,5-diphenyltetrazolium bromide (MTT) assay. Fluorescence assays showed that the AIEgen-DNA assemblies exhibit no significant co-localization with either mitochondria or endoplasmic reticulum, indicating that the cancer cell apoptosis caused by the HCR assembly was negligibly correlated to the direct interactions with endoplasmic reticulum (ER) or mitochondria (Fig. S49). To further investigate the specific apoptotic pathways triggered by ROS production under white light exposure in AIE photosensitizer-DNA conjugate assemblies, we first examined the expression level of the key apoptotic effector protein Caspase-3 using western blot analysis (Fig. 5j). The results revealed that with extending white light irradiation time, the expression of the inactive 32 kDa Caspase-3 gradually decreased, while its active cleaved form, ∼17 kDa, increased, indicating its activation during apoptosis. Then we investigated the expression level of Gasdermin E (GSDME) activated by the upstream Caspase-3 protein cleavage. As the duration of white light irradiation increased, the full-length GSDME proteins gradually cleaved and transformed into cleaved products (Fig. S50). We also investigated the expression level of Bax, an upstream regulator of Caspase-3 (Fig. 5j). It showed that with prolonged white light irradiation, Bax expression increased, thereby stimulating Caspase-3 activation and accelerating the apoptotic process (Fig. 5i). No matter whether assembly occurred or was white light irradiated, it was observed that the tumor necrosis factor-α (TNF-α) expression level was consistently low in the supernatants of cell mediums (Fig. S51). In summary, these results demonstrate that AIE photosensitizer-DNA conjugates can significantly enhance PDT activity and trigger apoptosis against cancer cells while maintaining low cytotoxicity in normal cells, holding great promise for targeted PDT *in vivo*.

### *In vivo* tumor imaging and augmented photodynamic therapy

**Figure 6.**
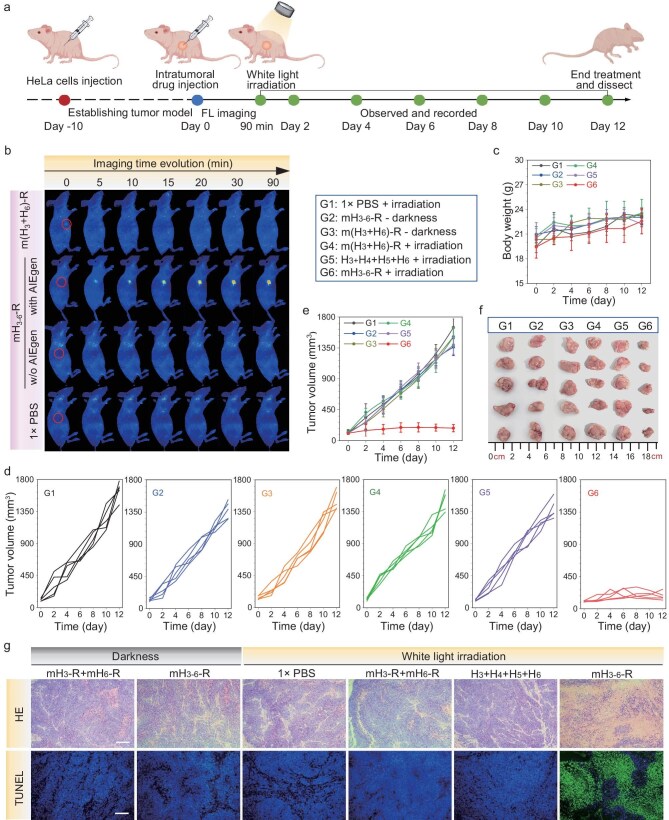
*In vivo* cancer imaging and cancer growth inhibition. (a) Schematic illustration of the antitumor experiment in mice. (b) Time evolution of fluorescence imaging of tumor-bearing mice after treatment with mH_3-6_-R, H_3_ + H_4_ + H_5_ + H_6_, (mH_3_-R + mH_6_-R) and 1× PBS excited by 455 nm laser. (c) The evolution of mouse weight curves after different treatments. (d) Individual tumor growth curves after different treatments. Data are mean ± SD (*n* = 5). (e) The evolution of tumor growth curves after different treatments. (f) Photographs of dissected tumors at the end of various treatments. (g) H&E and TUNEL staining images of tumor slices collected from tumor-bearing mice after treatment by mH_3-6_-R, H_3_ + H_4_ + H_5_ + H_6_, (mH_3_-R + mH_6_-R) and 1× PBS with or without white light irradiation. All scale bars are 200 μm.

Encouraged by the promising *in vitro* results, the *in vivo* antitumor effect of the AIE photosensitizer-DNA conjugates was further investigated using a tumor-bearing mouse model. Hemolysis assays demonstrated that incubation with various concentrations of mH_3-6_-R for 12 h did not induce lysis of red blood cells (Fig. S52), verifying the good hemocompatibility and safety of the AIE photosensitizer-DNA conjugates that are feasible for biomedical applications. As shown in Fig. 6a, tumor-bearing mice were randomly divided into six groups and treated with mH_3-6_-R via intra-tumoral injection, followed by white light irradiation or darkness. Control groups received treatment with (mH_3_-R + mH_6_-R) under white light irradiation or darkness, or with naked (H_3_ + H_4_ + H_5_ + H_6_) hairpins or 1× phosphate-buffered saline (PBS) under white light irradiation. Subsequently, the fluorescence from the AIE photosensitizer assemblies in the tumor region was monitored at predetermined time intervals. As depicted in Fig. 6b, significantly enhanced fluorescence signals were observed in the tumor region following the injection of mH_3-6_-R, while mice treated with (mH_3_-R + mH_6_-R) or naked (H_3_ + H_4_ + H_5_ + H_6_) hairpins or 1× PBS showed negligible fluorescence signals. TEM images of excised tumor tissue showed obvious 2-D dendritic-type assemblies inside the cytoplasm with a much darker contrast different from the other cytoplasm area (Fig. S53a). Additionally, AFM imaging of the lysate from tumor tissue also revealed clear 2-D dendritic-type assemblies, verifying the occurrence of the HCR assembly of AIE-DNA conjugates within the complex tumor tissue (Fig. S53b). The PDT activity of the assemblies within the tumor-bearing mice was subsequently evaluated. As recorded in Fig. 6d–f, tumor growth in the groups treated with mH_3-6_-R or (mH_3_-R + mH_6_-R) in darkness remained almost unaffected, similar to the 1× PBS group, indicating minimal dark toxicity associated with the AIE photosensitizer-DNA conjugates. Notably, tumor growth was significantly suppressed in the mH_3-6_-R group after 30 min white light irradiation. In contrast, in the groups treated with (mH_3_-R + mH_6_-R) or (H_3_ + H_4_ + H_5_ + H_6_) hairpins, which were unable to assemble, tumor growth was only minimally inhibited, even though they received an equivalent dose of conjugates. Importantly, no significant decrease in body weight was observed across all groups during the entire experimental period (Fig. 6c), ruling out concerns of systemic toxicity.

Further histological assessments using hematoxylin and eosin (H&E) staining and terminal deoxynucleotidyl transferase-mediated dUTP nick end labeling (TUNEL) staining provided evidence of significant tumor cell death in the mH_3-6_-R conjugate-treated groups under white light irradiation (Fig. 6g). *In vivo* toxicity assessment indicated that the probes exhibited good biosafety for *in vivo* therapy (Fig. S54). These findings collectively demonstrated that, in response to miRNA biomarkers, the assembly of AIE photosensitizer-DNA conjugated probes induced remarkable PDT activity, efficiently inhibiting tumor growth while showing negligible toxicity. This highlighted their potential for precise and augmented tumor therapy.

## CONCLUSIONS

In this contribution, we have developed a dimension-controllable and kinetics-accelerated assembly strategy of AIE photosensitizers upon cancer biomarker response, achieving *in situ* precise tumor therapy. A series of AIEgens with diverse conjugation tags and emission wavelengths were designed and synthesized, followed by click reaction with hairpin DNAs to prepare photosensitizer-conjugated DNA probes. Upon triggering by specific miRNA biomarkers, AIE photosensitizers programmed and assembled within tumor cells via the HCR reaction, leading to the emergence and boosting of photodynamic activity from scratch. The accompanied turn-on fluorescence signal exhibited an ultrahigh signal-to-noise ratio, and even miRNA-21 at picomolar concentration can trigger the assembly, allowing for real-time monitoring of the subtle miRNA variation in living cells with single-nucleotide discrimination. Further, by precisely controlling the DNA conjugation valencies on AIE photosensitizers, dimension-controllable assembly was achieved with production of 1-D linear-, 2-D dendritic-, and 3-D spherical-type assembly structures, thereby enhancing the stability and anti-interference capability of the output imaging and therapeutic properties. Conventional AIE fluorophores and photosensitizers typically aggregate and generate ROS through changes in solubility, binding to target molecules, hydrophobic interactions, and electrostatic adsorption, etc. [46]. These aggregation states are inherently uncontrollable and structurally disordered, leading to unstable fluorescence signals and substantial variability in photosensitizer performance [47]. By contrast, triggered by specific intracellular nucleic acid-type tumor biomarkers and guided by DNA strand hybridization reactions, programmable and dimension-controllable assembly enables ordered aggregation of AIE photosensitizers *in situ*, markedly improving both the efficiency and stability of fluorescence and ROS generation. Meanwhile, an HCR process specifically triggered by target miRNA not only yields significantly enhanced fluorescence signals but also affords selective, image-guided PDT that can successfully distinguish tumor cells from normal cells, thereby greatly reducing the side effects of PDT and enhancing its therapeutic selectivity and efficiency. Notably, the photosensitizer conjugation markedly reduced the hairpin DNA melting temperature, whereas their assembly exhibited an elevated melting temperature, thereby accelerating the HCR reaction within a few minutes and promoting their reactivities in the intracellular environment. This *in situ* dimension-controllable and accelerated assembly strategy triggered by specific response to tumor biomarkers provides a promising approach for early and precise cancer theranostics.

## METHODS

### Synthesis and characterization of AIEgen-DNA conjugates

AIEgen-DNA conjugates were synthesized by copper-free click chemistry. DBCO groups will preferentially and spontaneously label AIEgen-containing azide groups (–N_3_). The monomeric click reaction occurred in a 1 : 1 volume ratio of deionized water to dimethyl sulfoxide (DMSO). The stock solutions of hairpin DNA strands (including H_1_, H_2_, H_3_, H_4_, H_5_, and H_6_) and AIEgen (including mY-N_3_ and mR-N_3_) were added in a molar ratio of 1 : 1.5 and reacted overnight at room temperature with vibration. The dimeric click reaction occurred in a 1 : 1 volume ratio of sodium citrate-HCl buffer to DMSO. The stock solutions of hairpin DNA strands (including H_7_ and H_8_) and AIEgen (including dY-N_3_ and dR-N_3_) were added in a molar ratio of 2.5 : 1 and reacted overnight at room temperature with shaking. The trimeric click reaction occurred in a 1 : 1 volume ratio of sodium citrate-HCl buffer to DMSO. The stock solutions of hairpin DNA strands (including H_7_) and AIEgen (including dY-N_3_) were added in a molar ratio of 3.5 : 1 and reacted overnight at room temperature with shaking.

AIEgen-DNA conjugates were purified by high-performance liquid chromatography (HPLC, Agilent) on a 4.6 × 150 mm Agilent C-18 reverse-phase analytical column connected to a diode-array detector (DAD) monitor. The following gradient system was used at detection wavelengths of 260 nm, 365 nm, and 400 nm at a flow rate of 1.5 mL per minute: phase A is ammonium acetate (5 mM) buffer; phase B is acetonitrile. The solution was lyophilized to yield colorless solids, which were kept at −20°C. Deionized water was added to dissolve the AIEgen-DNA conjugates to yield a stock solution with known concentrations. The conjugates’ mass spectrometry was tested by Thermo linear ion trap (LTQ). Quantitation of conjugates was performed using approximate extinction coefficients of DNA with *λ*_max_ = 260 nm and AIEgens with *λ*_max_ = 365 nm.

### Assembly of AIEgen-DNA conjugates

The assembly of AIEgen-DNA conjugates occurred via hybridization chain reaction (HCR). The reaction started after adding various molar ratios of Initiator strand to hairpin DNA strand (2 μM) in 1× PBS buffer and was incubated at room temperature for 20 min before fluorescence spectra measurement and non-denaturing PAGE.

### Morphological characterization of HCR reaction product of AIEgen-DNA

The surface of a freshly cleaved mica substrate was positively charged by using 0.1% polylysine (PLL). The AIEgen-DNA conjugate assemblies (5 nM, 30 μL) were deposited onto the mica surface and left to adsorb to the surface for 20 min, washed with 50 μL of water for more than 3 times and dried with compressed air. A MultiMode V8 AFM (Bruker) system was used to image the samples under ScanAsyst-Air mode.

Exactly 10 μL of each sample was dispersed onto clean silicon wafers or glass slides and dried at room temperature. SEM characterization was performed on a HITACHI S-4700 field emission scanning electron microscope. Fluorescence microscopy characterization was captured on an Olympus IX-71 inverted fluorescence microscope.

### miRNA imaging in living cells

A total of 200 μL of HeLa, B16, MCF-7, HepG-2, or HEK293 cells (1.5 × 10^4^ cells per well) were seeded into 8-well chamber slides. After incubation overnight, the cells were washed once with 1× PBS. A 200 μL mixture containing 0.6 μL Lipofectamine^TM^ 2000 and 10 μM AIEgen-DNA conjugates were added into each well and incubated at 37°C in a humidified incubator for 1 h. Subsequently, the minimal essential medium (MEM) was replaced by fresh FBS-containing cell media and the cells were further incubated for up to 3 h. All the cells were washed twice with 1× PBS for confocal laser scanning microscopic imaging (Leica TCS SP5 II). For co-localization experiments, the cells were first incubated with nanoprobes, washed twice with 1× PBS, and then incubated with 200 μL Hoechst (0.2 mg mL^−1^) at 37°C for 20 min, and washed three times with 1× PBS. Cell images were acquired on a confocal laser scanning microscope (Leica TCS SP5 II) using a 63 × oil immersion objective.

### *In vivo* imaging and therapy

All procedures of animal experiments were approved by the Animal Care and Use Committee of Soochow University and complied with all relevant ethical regulations. The BALB/c mice (female, 6–8 weeks) were bought from Soochow University Laboratory Animal Center. Tumor models were established by injecting HeLa cells (1 × 10^6^ cells in 100 μL of PBS) subcutaneous at the right back of the mice. Tumor volume was calculated using the following formula: volume = width^2^ × length/2. When the tumor volume reached the appropriate size, the tumor-bearing mice were used for experiments.

HeLa tumor-bearing mice were randomly divided into seven groups (*n* = 5) for the intratumor injection of 1× PBS, mH_3-6_-R, (mH_3_-R + mH_6_-R), H_3_ + H_4_ + H_5_ + H_6_ under dark and white light irradiation, respectively. At predetermined time intervals, the mice were anesthetized with isoflurane and imaged by Maestro In-Vivo Imaging System (Caliper Life Science) equipped with a 455 nm laser (power density = 0.3 W cm^−2^) and an 850 nm short pass emission filter (Chroma).

### Statistical analysis

Statistical significances were determined by Student’s *t*-test, with *P* < 0.05 defined as significant (denoted by asterisks in graphs) and *P* > 0.05 defined as not significant.

## Supplementary Material

nwaf424_Supplemental_File
