# Supplementary material for: MicroRNA-triggered in situ programmed assembly of photosensitizers with controlled dimension and accelerated kinetics for precise cancer therapy
Source: Natl Sci Rev. 2025 Sep 29;13(1):nwaf424. doi: 10.1093/nsr/nwaf424 (PMC12796811; doi:10.1093/nsr/nwaf424)
Supplement: nwaf424_Supplemental_File [file nwaf424_supplemental_file.pdf]

Supplementary Information

MicroRNA-triggered in situ programmed assembly of photosensitizers with controlled dimension and accelerated kinetics for precise cancer therapy

Jie Sun[1], Ling-Hong Xiong[2], Ben Zhong Tang[3,4,], Xuewen He[1,]**

[1]State Key Laboratory of Bioinspired Interfacial Materials Science, The Key Lab of Health Chemistry and Molecular Diagnosis of Suzhou, College of Chemistry, Chemical Engineering and Materials Science, Soochow University, Suzhou 215123, China

[2]School of Public Health, Suzhou Medical College of Soochow University, Soochow University, Suzhou 215123, China

[3]Guangdong Basic Research Center of Excellence for Aggregate Science, School of Science and Engineering, The Chinese University of Hong Kong, Shenzhen (CUHK-Shenzhen), Guangdong 518172, China

[4]Department of Chemistry, Hong Kong Branch of Chinese National Engineering Research Centre for Tissue Restoration and Reconstruction, Institute for Advanced Study and Division of Life Science, The Hong Kong University of Science and Technology, Clear Water Bay, Kowloon 00852, Hong Kong

*E-mail: tangbenz@cuhk.edu.cn; xheao@suda.edu.cn

Materials

Titanium tetrachloride ($TiCl_4$, 99.9%), n-butyllithium (n-BuLi, 2.0 M in hexane), potassium carbonate (K_2CO_3, AR), bis[4-(diethylamino)phenyl]methanone, bis(4-methoxyphenyl)methanone, benzophenone, (4-bromophenyl)(phenyl)methanone, N,N-dimethylbenzamide, malononitrile, pyridine and boron tribromide (BBr_3, 1.0 M in DCM solution) were purchased from J&K. Dimethyl sulfoxide (DMSO, 99.7%) was purchased from Sigma-Aldrich. N-hexane, Chloroform ($CHCl_3$), dichloromethane (DCM), tetrahydrofuran (THF), N, N-dimethylformamide (DMF), methanol, ethanol and ethyl acetate were purchased from Energy Chemical. Try3-(4,5-dimethylthiazol-2-yl)-2,5-diphenyltetrazolium bromide (MTT, 97.5%), hydroxyphenyl fluorescein (HPF) and dihydrorhodamine 123 (DHR 123) were purchased from Sigma-Aldrich. Hydrochloric acid (HCl, GR), N,N,N',N'-tetramethylethylenediamine (TEMED, 98%) and ammonium persulfate (APS, 98%) were purchased from Sinopharm Chemical Reagent Co., Ltd. Acrylamide/Bis solution (40% (*w*/*v*)) was purchased from Bio-Rad. 4SGelred was purchased from Sangon Biotechnology Co., Ltd. (Shanghai, China). Agarose powder was purchased from Biowest. Glycerol and tris(hydroxymethyl)aminomethane (Tris) were purchased from Beijing Solarbio Science &Technology Co., Ltd. Phosphate buffered saline (10× PBS) was purchased from Thermo Scientific (HyClone). Lipofectamine-2000, Hoechst 33342, 2,7-Dichlorodihydrofluorescein diacetate (DCFH-DA), Calcein AM/PI double stain kit and Lysotracker green was purchased from Invitrogen. CHLORIN E6 (Ce6) and 5,5-Dimethyl-1-pyrroline N-oxide (DMPO) were purchased from Aladdin. RIPA Lysis Buffer was purchased from Beijing GenStar BioSolutions Co., Ltd. BAX Monoclonal antibody, Caspase 3/P17/P19 Polyclonal antibody, DFNA5/GSDME Polyclonal antibody was purchased from Proteintech. β-actin rabbit mAbs, anti-rabbit IgG labeled HRP were obtained from Abcam. Annexin V-FITC Apoptosis Detection Kit was purchased from Beyotime Biotechnology. All cells were purchased from China Center for Type Culture Collection (CCTCC). Dulbecco's Modified Eagle's Medium (DMEM), fetal bovine serum (FBS), and trypsin/EDTA (0.25%) were purchased from

Hyclone. All DNA strands (see Table S1) were synthesized and HPLC purified by Sangon Biotechnology Co., Ltd. (Shanghai, China). Water (18.2 MΩ) was purified by Milli-Q Direct-8 water purification system (Millipore). All other reagents and solvents are of analytical grade.

DNA sequences

H_1 for 1D assembly:

5'-/DBCO/TCAACATCAGTCTGATAAGCTACTAGAGCCTAGCTTATCAGACT-3'

H_2 for 1D assembly:

5'-TAGCTTATCAGACTGATGTTGA/iDBCOdT/GTCTGATAAGCTAGGCTCTAG -3'

H_3 for 2D assembly: 5'-/DBCO/

TCAACATCAGTCTGATAAGCTACACTAGAGCCTGTAGCTTATCAGACTTGACGAAC

TC-3'

H_4 for 2D assembly: 5'-

TGTAGCTTATCAGACTGATGTTGAA/iDBCOdT/AAGCTACAGGCTCTAG-3'

H_5 for 2D assembly:

5'-TGACGAACTCCTAGAGCCCGAAGTAGGAGTTCGTCAAGTCTG/DBCO/-3'

H_6 for 2D assembly: 5'-

CTACTTCGGGCTCTAGGAGTTCGTCACAGACTTGACGAACTCCTAGAGCCTGTAGC

TTAT/DBCO/-3'

H_7 for 3D assembly: 5'-/DBCO/

TCAACATCAGTCTGATAAGCTACTAGAGCCTAGCTTATCAGACT-3'

H_8 for 3D assembly: 5'-

TAGCTTATCAGACTGATGTTGAAGTCTGATAAGCTAGGCTCTAG/DBCO/-3'

H_9 for control of 3D assembly: 5'-/DBCO/

TTTTTTTTCGCGTACGTACGTATTTTTTTTTACGTACGTACGCG-3'

In vitro Initiator (miR-21) (I'): 5'-TAGCTTATCAGACTGATGTTGA-3'

(**miRNA-21 Initiator (I)**: 5'-UAGCUUAUCAGACUGAUGUUGA-3')

In vitro non-target DNA (miR-141): 5'-TAACACTGTCTGGTAAAGATGG-3'

One-base mismatched miRNA-21 (mis1-miR-21):

5'-TAGCTTATCAGACTGTTGTTGA-3'

Three-base mismatched miRNA-21 (mis3-miR-21):

5'-TACCTTACCAGACTGTTGTTGA-3'

Dynamic light scattering and Zeta potential measurement

Dynamic light scattering (DLS) and zeta potential measurements were performed on a Zetasizer Nano ZS90 (Malvern) with 90 ° scattering angle and a He-Ne laser.

ROS generation measurements

DCFH was used to measure the generated total ROS. A DCFH stock solution (10 μM, 100 μL) was added into the assemblies produced by HCR reaction of AIEgen-DNA conjugates (5 μM in 100 μL 1× PBS buffer). White light (2 mW•cm^{-2}) was used to irradiate the suspensions. The FL intensity of DCFH at 525 nm (488 nm excitation) was recorded at desired time points. Similarly, DHR 123 stock solution (10 μM, 100 μL) were added to the assemblies produced by HCR reaction of AIEgen-DNA conjugates (5 μM in 100 μL 1× PBS buffer). Under 505 nm excitation, the emission maximum intensities were employed for the tracking of the generation of superoxide ($O_2^{\bullet-}$). DMPO is a water-soluble one-electron oxidant spin trap that can measure ROS at room temperature. Electron spin resonance (ESR) spectroscopy of the assemblies produced by HCR reaction of AIEgen-DNA conjugates was tested with DMPO in MeOH after 10 min white light irradiation.

Cell Culture

Cell lines including HeLa, MCF-7, B16, HepG-2 and HEK-293 were cultured on 25 cm^2 cell culture plates with vent caps (Corning) in DMEM supplemented with 10% fetal bovine serum and 1% antibiotics penicillin/streptomycin (100 U•mL^{-1}). MCF-7 cells were cultured in DMEM supplemented with 10% fetal bovine serum, 0.01 mg•mL^{-1} insulin, and 1% antibiotics penicillin/streptomycin (100 U•mL^{-1}). All the cells were grown in a humidified incubator at 37 ℃ containing CO_2 (5%). Cells grown to sub-confluence were dissociated from the surface

with a solution of 0.25% trypsin/EDTA. Then aliquots of cells were seeded into 96-well plate (Corning) or 8-well chamber slide (Lab-Tek) and grown for required duration in FBS-containing cell media before experiments.

Intracellular ROS detection

The intracellular ROS detection by fluorescence microscope was carried out as following: HeLa cells were seeded in a 96-well plate (Corning), and cultured at 37 ℃ for 24 h. The medium was replaced with fresh cell culture medium containing AIEgen-DNA conjugates. After 4 h incubation, the medium was removed. The cells were washed thoroughly with 1× PBS, and DMEM medium containing 10 μM DCFH-DA was added. The cells were incubated at 37 ℃ for another 20 min. Then the cells were washed and irradiated by white light for 30 min. The treated cells were imaged by fluorescence microscope with a 10× objective.

Cell toxicity assay

Aliquots (100μL) of HeLa and HEK-293 cells were seeded (1.5×10^4 cells) into a 96 well plate. After overnight incubation, cell media were removed and the cells were washed 3 times with 1× PBS. Next, 100 μL of cell media containing 0, 1, 2, 3, 4, 5 μM AIEgen-DNA conjugates were added to each well, and the cells were incubated at 37℃ for 4 h before thoroughly washing with 1×PBS. After irradiation for 0, 5, 10, 20, 30 min under white light, the cells were further incubated for 24 h. Then the media were replaced with 100μL of cell media containing 0.5 mg•mL^{-1} 3-(4,5-Dimethylthiazol-2-yl)-2,5-diphe-nyltetrazolium Bromide (MTT), and the cells were incubated at 37℃ for another 4 h. Formazan crystals were dissolved in 100 μL of dimethyl sulfoxide (DMSO) with gentle-agitation for 10 min. Cells treated with FBS-containing media alone and Triton X-100 were used as low- and high-cell death controls, respectively. The absorbance of the supernatant at 560 nm was measured using a TECAN Infinite M200 PRO plate reader.

Live/dead cell staining

Cells (1.5×10^4 cells) were plated into 96 well plate (Costar) with treatment of AIEgen-DNA

conjugates and whit light irradiation. Then, the cells were stained with Calcein AM/PI double stain kit (4 μM) under the instructions of the manufacturer for fluorescence imaging and Flow Cytometry.

Western blot analysis

The western blot (WB) assay was used to detect Bax, GSDME, cleaved GSDME, Caspase 3 and cleaved Caspase 3 levels in HeLa cells after different treatments. First the total protein of HeLa cells was extracted with cell lysis buffer (Cell Signaling Technology) containing protease inhibitor "cocktail" (Calbiochem) and 1 mM phenylmethysulfonyl fluoride (PMSF). The lysis samples (40 μg) were added to the 10% SDS-PAGE gels, allowed to run for 90 min and then transferred to nitrocellulose membranes running for 200 mA constant current 60 min. The membranes were blocked with 5% skim milk in 1× TBST buffer for 1 h at room temperature, and then incubated with BAX Monoclonal antibody, DFNA5/GSDME Polyclonal antibody, Caspase 3/P17/P19 Polyclonal antibody and rabbit anti-β-actin mAbs at 4 °C overnight. The membrane was then washed by 1× TBST buffer for five times and incubated with HRP labeled goat anti-mouse IgG and HRP labeled goat anti-rabbit IgG in 1× TBST buffer containing 5% skim milk at room temperature for 1 h. The membrane was then washed with 1× TBST buffer for five times, and further added pierce ECL western blotting substrate for chemoluminescence measurement. The images of the WB were captured and analyzed using the GE Amersham Imager600 imaging system.

TUNEL and H&E staining

The extracted tissues were fixed in 4% paraformaldehyde, paraffin embedded, and sectioned for Hematoxylin–Eosin (H&E) and TUNEL staining using standard protocols. And they were finally examined using an inverted microscope.

Synthesis of AIEgens

Synthesis of compound **mB-N$_3$** or **dB-N$_3$**. The mixture of (2-(4-(bromomethyl) phenyl) ethene-1,1,2-triyl) tribenzene or (E)-1,2-bis(4-(bromomethyl) phenyl)-1,2-diphenylethene

(0.64 g or 0.78 g, 1.5 mmol) and sodium azide (0.39 g, 6 mmol) in 8 mL DMSO was stirred at room temperature overnight. Then the mixutre was poured into water and extracted with ethyl ether. The organic layer was washed with brine, dried over sodium sulfate and dried over anhydrous sodium sulfate and evaporated to dryness. The residue was subjected to column chromatography with dichloromethane and petroleum (1:3 by volume) as eluent to afford compound **mB-N_3** (0.35 g, 0.9 mmol) or **dB-N_3** (0.38 g, 0.93 mmol) was obtained as white solid in 86% yield. **mB-N_3**: ^{1}H NMR (Bruker Avance, 400 MHz, $CDCl_3$), δ (ppm): 7.12-7.10 (m, 9H), 7.05-7.01 (m, 10H), 4.25 (s, 2H). ^{13}C NMR (Bruker Avance, 101 MHz, $CDCl_3$) δ (ppm): 144.0, 143.7, 143.6, 143.6, 141.6, 140.4, 133.4, 131.9, 131.4, 127.9, 127.9, 127.8, 127.7, 126.7, 126.7, 126.6, 54.7. HRMS (ESI+): calculated for $C_{27}H_{21}N_3$ [M+]: 387.1737; found: 387.1735. **dB-N_3**: ^{1}H NMR (Bruker Avance, 400 MHz, $CDCl_3$), δ (ppm): 7.33-7.31 (t, 7H), 7.26-7.22 (m, 11H), 4.45 (s, 4H). ^{13}C NMR (Bruker Avance, 101 MHz, $CDCl_3$) δ (ppm): 143.7, 143.7, 143.2, 140.8, 140.8, 133.4, 133.4, 131.7, 131.3, 131.3, 127.8, 127.8, 127.7, 127.6, 126.7, 126.7, 54.5. HRMS (ESI+): calculated for $C_{28}H_{22}N_6$ [M+]: 442.1909; found: 442.1906.

Synthesis of compound **1**. Sodium azide (11.66 g, 0.18 mol) was added to a solution of 3-chloropropan-1-ol (5 ml, 59.76 mmol) in DMF (40 mL). The reaction mixture was heated at 60 °C for 24 h. Then it was diluted four times with water and extracted with diethyl ether three times. The organic layer was dried over Na_2SO_4 and concentrated in vacuo. The crude compound (6 g, 99%) was used in to the next step without purification.

To the solution of the crude compound in dichloromethane (60 mL) were added DMAP (1.45 g, 11.88 mmol) and Et_3N (12.42 mL, 89.12 mmol). And then a solution of *p*-toluenesulfonyl chloride (17 g, 89.12 mmol) in dichloromethane (30 mL) was added dropwise at 0 °C. The reaction mixture was stirred at 0 °C for 30 min and warm to room temperature. After 12 h of stirring, it was diluted with dichloromethane (100 mL) and washed with saturated aqueous $NaHCO_3$, 10% aqueous HCl, and 10% brine sequentially. The organic layer was dried over Na_2SO_4 and concentrated in vacuo. The purification by flash column chromatography (3: 1 hexane/ethyl acetate) gave **1** as a colorless oil (12 g, 80.4%).

Synthesis of compound **2-1, 2-2 and 2-3**. To a THF solution (100 mL) contained

4-methoxyphenyl methanone (2.00 g, 9.12 mmol) or bis(4-methoxyphenyl) methanone (2.00 g, 8.26 mmol), (4-bromophenyl) (phenyl)methanone (2.34 g, 9.00 mmol) and zinc powder (3.15 g, 33.04 mmol), $TiCl_4$ (3.63 mL, 33.04 mmol) was dropwise added at 0 ℃ in ice bath. Then the mixture was heated to reflux for 6 h. The reaction was then quenched by 50 mL HCl (5.0 M), and the compound was extracted with DCM. The collected organic layer was dried over anhydrous sodium sulfate and evaporated to dryness. The residue was subjected to column chromatography using DCM and n-hexane mixture (1:3, v/v) as eluent to afford compound **2-1** (1.72, 3.64mol) or **2-2** (1.65 g, 3.51 mmol) or **2-3** (1.62 g, 3.46 mmol) as gray solids in 43.1% yield.

Synthesis of compound **3-1**, **3-2 and 3-3**. To a THF solution (60 mL) contained compound **2-1** or **2-2** or **2-3** (1.00 g, 2.13 mmol), n-BuLi (2.00 mL, 4.00 mmol) was dropwise added at -78 ℃ in dry ice-acetone bath. After reacted for 1.0 h, N, N-dimethylbenzamide (724 μL, 5.00 mmol) was swiftly injected. Then the temperature was slowly warmed to room temperature and the reaction was lasted for 10 h. The reaction was then quenched with 10 mL methanol, and the solvent inside was evaporated to dryness. The residue was subjected to column chromatography using DCM and n-hexane mixture (1:2, v/v) as eluent to afford compound **3-1** (1.01 g, 1.92 mmol) or **3-2** (0.93 g, 1.87 mmol) or **3-3** (0.96 g, 1.89 mmol) as a yellow solid in 87.8 % yield.

Synthesis of compound **4-1**, **4-2 and 4-3**. To a dry DCM solution (40.0 mL) of compound **3-1, 3-2 or 3-3** (0.30 g, 0.55 mmol) at 0 ℃ in ice bath, BBr_3 (2.21 mL, 2.21 mmol) was slowly added. The mixture was stirred for 12 h at room temperature. The reaction was quenched by methanol and the mixture was extracted with DCM. The collected organic layer was dried over anhydrous sodium sulfate. The crude product was condensed and purified on a silica-gel column using DCM as eluent. Compound **4-1** (0.18 g, 0.24 mmol) or **4-2** (0.19 g, 0.21 mmol) or **4-3** (0.21 g, 0.20 mmol) was obtained as dark red solid in 67.2% yield.

Synthesis of compound **mY-N$_3$**, **dY-N$_3$ and tY-N$_3$**. The mixture of compound **4-1** or **4-2** (0.12 g, 0.23 mmol), compound **1** (0.06 g, 0.25 mmol or 0.14 g, 0.55 mmol) and K_2CO_3 (95 mg, 0.69 mmol) in 8 mL DMF was heated at 80 ℃ overnight. Then the mixutre was poured into water and extracted with ethyl acetate. The organic layer was washed with brine, dried over

sodium sulfate and dried over anhydrous sodium sulfate and evaporated to dryness. The residue was subjected to column chromatography with dichloromethane and petroleum (1:1 by volume) as eluent to afford compound **mY-N$_3$** (0.21 g, 0.22 mmol) or **dY-N$_3$** (0.13 g, 0.19 mmol) or **tY-N$_3$** (0.18 g, 0.20 mmol) was obtained as red oil in 82.1% yield. **mY-N$_3$**: ^{1}H NMR (Bruker Avance, 600 MHz, CD$_3$CN), δ (ppm): 7.68-7.65 (t, 2H), 7.61-7.58 (t, 1H), 7.51-7.46 (m, 4H), 7.14-7.11 (m, 8H), 7.06-7.03 (m, 4H), 6.93-6.91 (d, 2H), 6.68-6.65 (m, 2H), 3.95-3.93 (m, 2H), 3.44-3.42 (t, 2H), 1.93-1.92 (m, 2H). ^{13}C NMR (Bruker Avance, 151 MHz, CD$_3$CN) δ (ppm): 196.8, 158.7, 149.6, 144.5, 144.3, 143.1, 140.3, 138.6, 136.6, 136.2, 133.3, 133.2, 133.2, 132.0, 131.9, 131.87, 130.6, 130.3, 130.2, 129.3, 128.9, 128.8, 128.8, 128.7, 127.7, 127.6, 114.7, 65.6, 48.9, 29.3. HRMS (ESI+): calculated for $C_{36}H_{29}N_3O_2$ [M+]: 535.2260; found: 535.2257. **dY-N$_3$**: ^{1}H NMR (Bruker Avance, 600 MHz, CD$_3$CN), δ (ppm): 7.70-7.68 (d, 2H), 7.63-7.60 (t, 1H), 7.52-7.49 (q, 4H), 7.18-7.13 (m, 5H), 7.07-7.05 (d, 2H), 6.95-6.94 (d, 2H), 6.71-6.67 (q, 4H), 3.99-3.96 (q, 4H), 3.47-3.45 (t, 4H), 1.99-1.96 (q, 4H). ^{13}C NMR (Bruker Avance, 151 MHz, CD$_3$CN) δ (ppm): 196.4, 158.4, 158.3, 149.5, 144.3, 142.5, 139.3, 138.4, 136.6, 136.5, 135.7, 133.0, 132.9, 131.7, 131.6, 130.2, 129.9, 128.9, 128.5, 127.1, 114.4, 114.2, 65.3, 48.6, 29.0. HRMS (ESI+): calculated for $C_{39}H_{34}N_6O_3$ [M+]: 634.2692; found: 634.2698. **tY-N$_3$**: ^{1}H NMR (Bruker Avance, 600 MHz, CD$_3$CN), δ (ppm): 7.70-7.69 (d, 2H), 7.63-7.61 (t, 1H), 7.52-7.49 (t, 4H), 7.15-7.13 (d, 2H), 6.96-6.92 (q, 6H), 6.72-6.69 (m, 6H), 3.99-3.96 (q, 6H), 3.48-3.45 (q, 6H), 1.99-1.96 (q, 4H). ^{13}C NMR (Bruker Avance, 151 MHz, CD$_3$CN) δ (ppm): 196.8, 158.7, 158.6, 158.5, 150.2, 142.1, 139.2, 138.8, 137.3, 137.2, 136.1, 133.4, 133.3, 133.3, 132.0, 130.6, 130.3, 129.3, 114.8, 114.7, 114.7 65.7, 49.0, 29.4. HRMS (ESI+): calculated for $C_{39}H_{34}N_6O_3$ [M+]: 734.3203; found: 734.3202.

Synthesis of compound **5-1** or **5-2**. To a DCM solution (60 mL) contained compound **3-1** or **3-2** (0.60 g, 1.21 mmol) and malononitrile (95.80 mg, 1.45 mmol), $TiCl_4$ (532 μL, 4.84 mmol) was dropwise added at 0 °C in ice bath. After the mixture was stirred for 30 min, pyridine (340 μL, 4.84 mmol) was injected to stir for another 30 min. Then, the mixture was heated at 40 °C for 4 h. The reaction was quenched by water and the mixture was extracted with DCM. The collected organic layer was dried over anhydrous sodium sulfate and evaporated to dryness. The residue was subjected to column chromatography using DCM and n-hexane

mixture (1:1, v/v) as eluent to afford compound **5-1** (0.64 g, 1.12 mmol) or **5-2** (0.59 g, 1.08 mmol) as a red solid in 89.6 % yield.

Synthesis of compound **6-1** or **6-2**. To a dry DCM solution (40.0 mL) of compound **5-1** or **5-2** (0.45 g, 0.61 mmol) at 0 °C in ice bath, BBr_3 (2.32 mL, 2.32 mmol) was slowly added. The mixture was stirred for 12 h at room temperature. The reaction was quenched by methanol and the mixture was extracted with DCM. The collected organic layer was dried over anhydrous sodium sulfate. The crude product was condensed and purified on a silica-gel column using DCM as eluent. Compound **6-1** (0.24 g, 0.23 mmol) or **6-2** (0.21 g, 0.21 mmol) was obtained as dark red solid in 67.9% yield.

Synthesis of compound **mR-N$_3$** or **dR-N$_3$**. The mixture of compound **6-1** or **6-2** (0.18 g, 0.25 mmol), compound **1** (0.06 g, 0.25 mmol or 0.14 g, 0.55 mmol) and K_2CO_3 (95 mg, 0.69 mmol) in 8 mL DMF was heated at 80 °C overnight. Then the mixture was poured into water and extracted with ethyl acetate. The organic layer was washed with brine, dried over sodium sulfate and dried over anhydrous sodium sulfate and evaporated to dryness. The residue was subjected to column chromatography with dichloromethane and petroleum (1:1 by volume) as eluent to afford compound **mR-N$_3$** (0.24 g, 0.23 mmol) or **dR-N$_3$** (0.16 g, 0.22 mmol) was obtained as red oil in 83.4% yield. **mR-N$_3$:** ^{1}H NMR (Bruker Avance, 600 MHz, CD_3CN), 7.71-7.60 (m, 1H), 7.54-7.50 (m, 2H), 7.42-7.37 (q, 2H), 7.23-7.02 (m, 14H), 7.07-7.05 (d, 2H), 6.96-6.90 (m, 2H), 6.72-6.68 (m, 2H), 3.99-3.96 (q, 2H), 3.48-3.45 (q, 2H), 1.99-1.94 (q, 2H). ^{13}C NMR (Bruker Avance, 151 MHz, CD_3CN) δ (ppm): 175.3, 158.8, 149.8, 144.3, 144.0, 143.6, 140.0, 137.4, 136.4, 135.1, 133.3, 132.2, 131.9, 131.2, 130.8, 130.6, 130.3, 129.7, 129.3, 128.9, 128.8, 128.7, 127.7, 115.2, 114.7, 79.1, 78.7, 65.6, 48.9, 29.3. HRMS (ESI+): calculated for $C_{39}H_{29}N_5O$ [M+]: 583.2372; found: 583.2375. **dR-N$_3$**: ^{1}H NMR (Bruker Avance, 600 MHz, CD_3CN), δ (ppm): 7.63-7.60 (t, 1H), 7.53-7.51 (t, 2H), 7.41-7.39 (d, 2H), 7.21-7.12 (m, 7H), 7.07-7.05 (d, 2H), 6.95-6.90 (q, 4H), 6.71-6.67 (q, 4H), 3.99-3.96 (q, 4H), 3.48-3.45 (q, 4H), 1.99-1.95 (q, 4H). ^{13}C NMR (Bruker Avance, 151 MHz, CD_3CN) δ (ppm): 174.9, 158.5, 158.3, 149.8, 144.0, 143.0, 138.9, 137.1, 136.4, 136.3, 134.6, 133.1, 133.0, 132.9, 131.9, 131.7, 130.8, 130.5, 129.3, 128.6, 127.2, 114.8, 114.2, 82.0, 65.3, 48.6, 28.9. HRMS (ESI+): calculated for $C_{42}H_{34}N_8NaO_2$ [M+]: 705.2702; found: 705.2708.

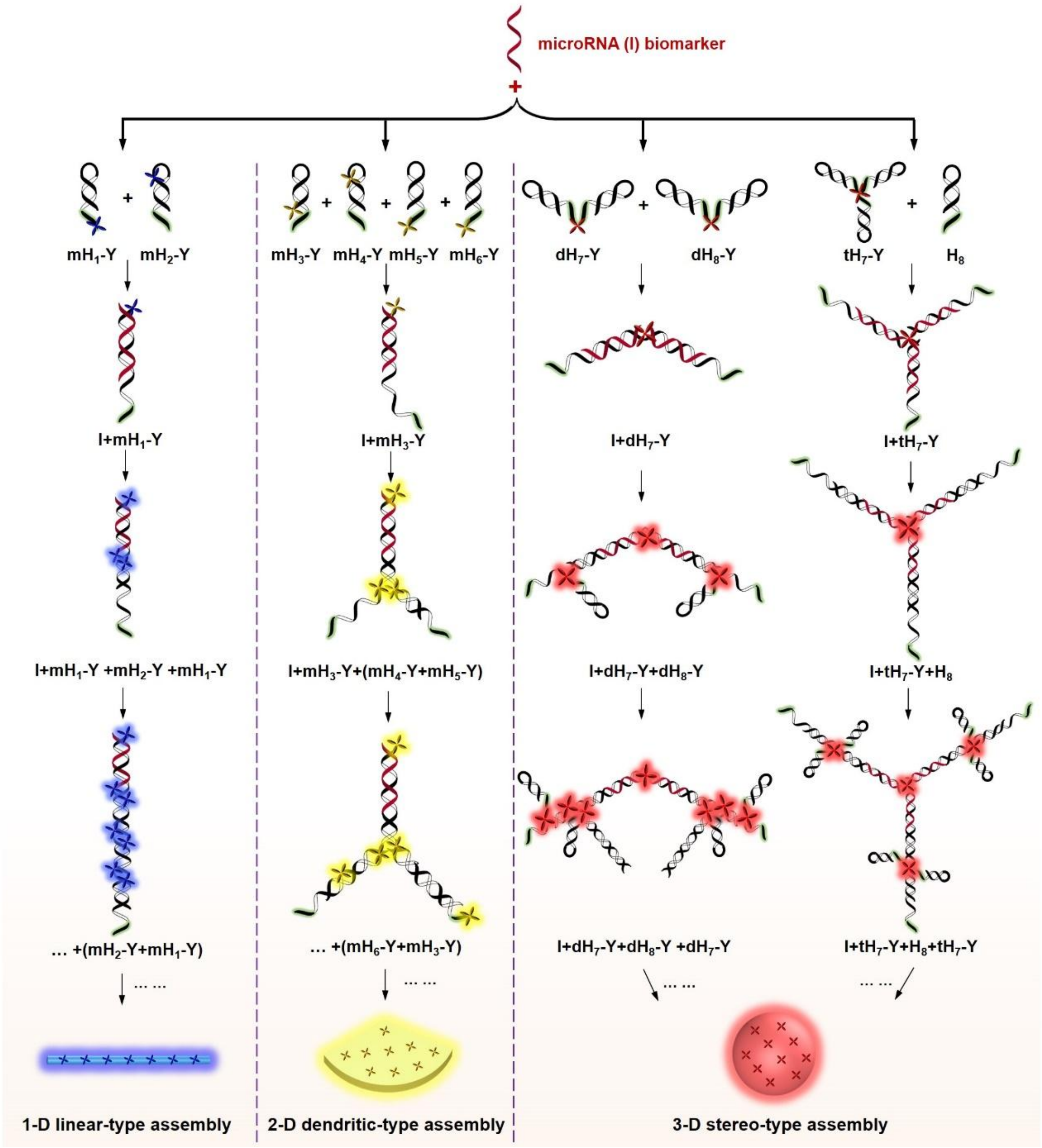

Figure S1. Illustration of the HCR assembly process of AIEgen-DNA conjugates. 1-D linear, 2-D dendritic and 3-D stereo-type assemblies were produced upon response to microRNA biomarker with turn-on AIE fluorescence.

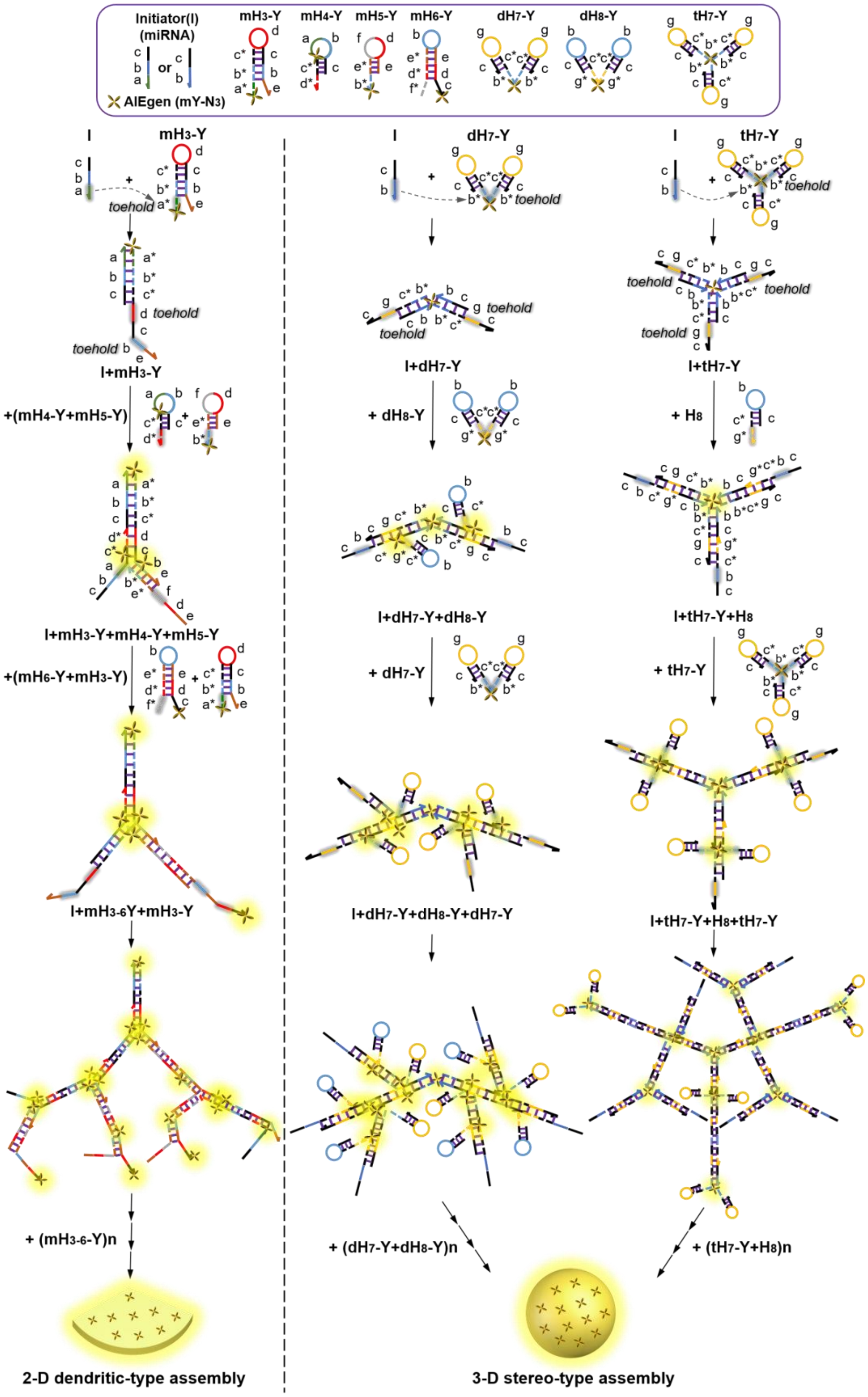

Figure S2. Detailed illustration of the HCR assembly process of AIEgen-DNA conjugates. 2-D dendritic- and 3-D stereo-type assemblies were produced upon response to miRNA biomarker with extending of DNA strand guided by HCR reaction, showing a gradually turn-on AIE fluorescence.

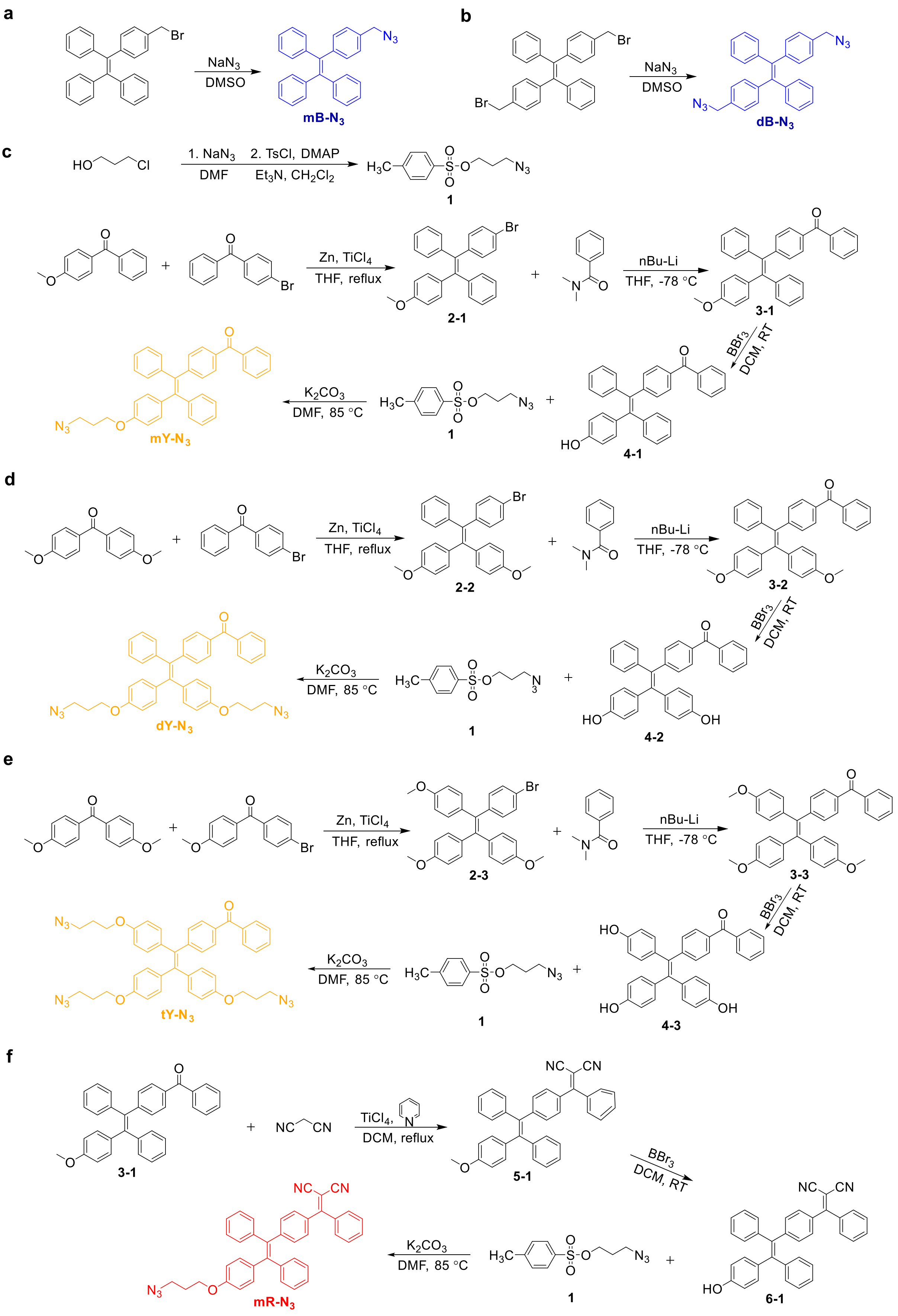
a
Br
NaN3
DMSO
N3
mB-N3
b
Br
Br
NaN3
DMSO
N3
N3
dB-N3
c
HO
Cl
1. NaN3
DMF
2. TsCl, DMAP
Et3N, CH2Cl2
H3C
S
O
O
O
N3
1
O
O
+
O
Br
Zn, TiCl4
THF, reflux
Br
O
2-1
+
N
O
nBu-Li
THF, -78 °C
O
O
3-1
BBr3
DCM, RT
O
N3
O
mY-N3
K2CO3
DMF, 85 °C
H3C
S
O
O
O
N3
1
+
O
HO
4-1
d
O
O
O
+
O
Br
Zn, TiCl4
THF, reflux
Br
O
O
2-2
+
N
O
nBu-Li
THF, -78 °C
O
O
O
3-2
BBr3
DCM, RT
O
N3
O
O
N3
dY-N3
K2CO3
DMF, 85 °C
H3C
S
O
O
O
N
3
1
+
O
HO
OH
4-2
e
O
O
O
+
O
O
Br
Zn, TiCl4
THF, reflux
O
Br
O
O
2-3
+
N
O
nBu-Li
THF, -78 °C
O
O
O
O
3-3
BBr3
DCM, RT
N3
O
O
N3
O
O
N3
tY-N3
K2CO3
DMF, 85 °C
H3C
S
O
O
O
N3
1
+
HO
O
HO
OH
4-3
f
O
O
3-1
+
NC
CN
TiCl4,
N
DCM, reflux
NC
CN
O
5-1
BBr3
DCM, RT
NC
CN
N3
O
mR-N3
K2CO3
DMF, 85 °C
H3C
S
O
O
O
N3
1
+
NC
CN
HO
6-1

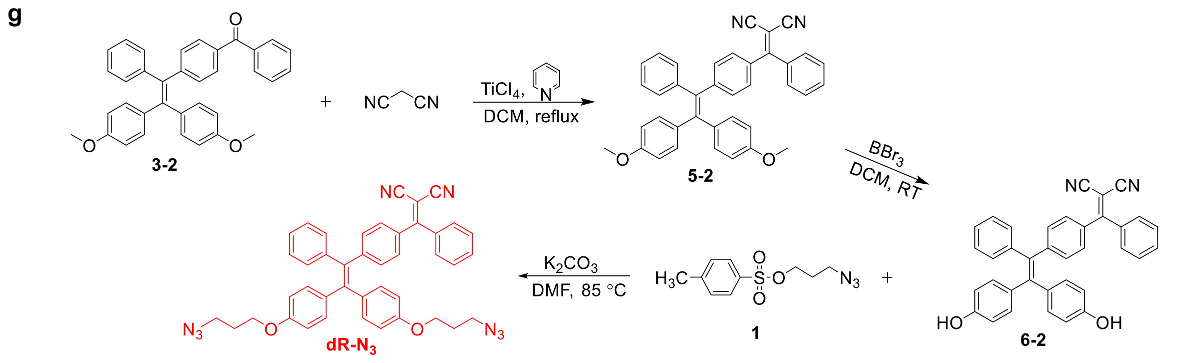

Figure S3. Synthesis routes for (a) **mB-N$_3$,** (b) **dB-N$_3$,** (c) **mY-N$_3$,** (d) **dY-N$_3$,** (e) **tY-N$_3$,** (f) **mR-N$_3$,** and (g) **dR-N$_3$**.

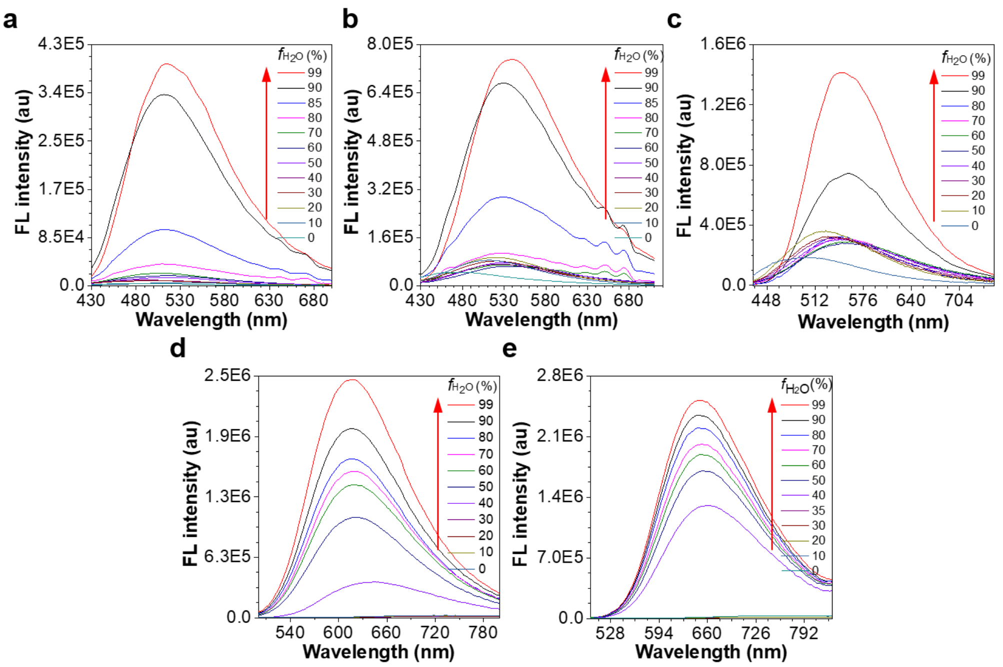

Figure S4. AIE curve of (a) mY-N$_3$, (b) dY-N$_3$, (c) tY-N$_3$, (d) mR-N$_3$ and (e) dR-N$_3$ in the mixture of THF and H_2O with varying volume fractions of H_2O.

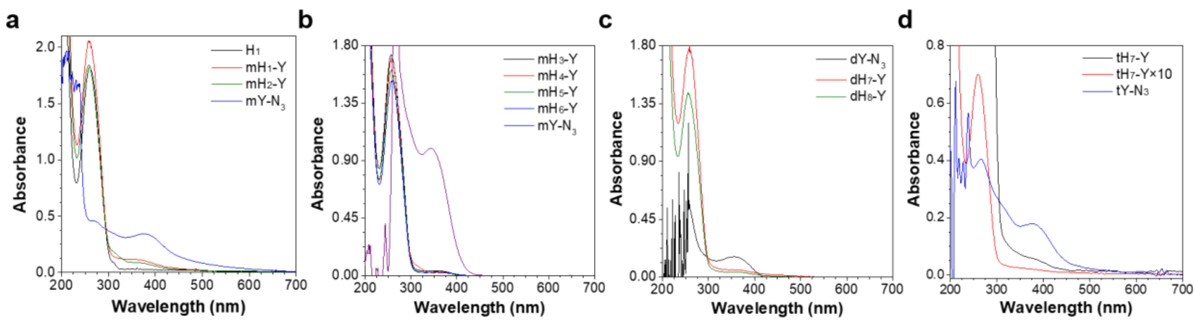

Figure S5. Absorption spectra of (a) H_1, mH_1-Y, mH_2-Y and mY-N$_3$, (b) mH_3-Y, mH_4-Y, mH_5-Y, mH_6-Y and mY-N$_3$, (c) dH_7-Y, dH_8-Y and dY-N$_3$, (d) tH_7-Y and tY-N$_3$.

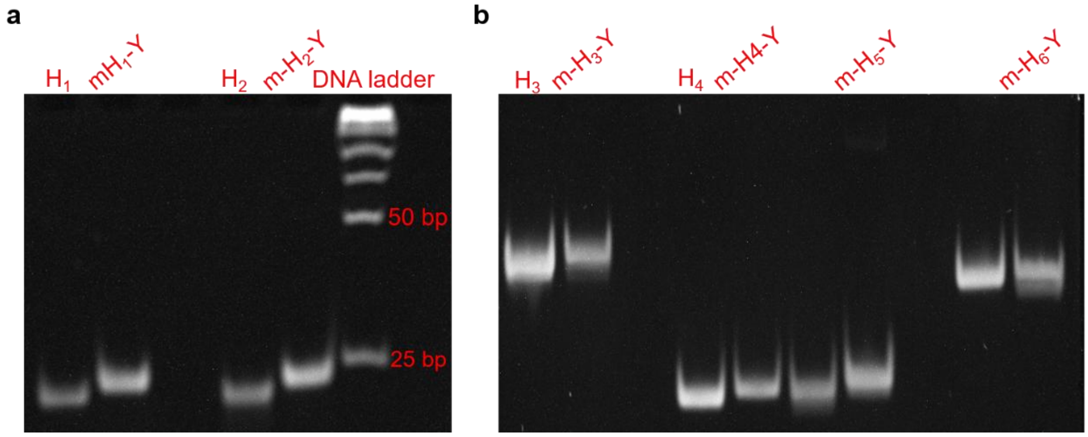

Figure S6. Non-denature PAGE gel electrophoresis of AIEgen-DNA conjugates for (a) 1-D and (b) 2-D assembly.

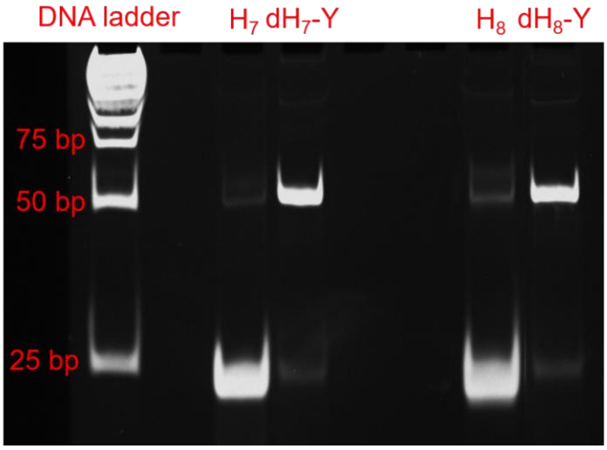

Figure S7. Non-denature PAGE gel electrophoresis of AIEgen-DNA conjugates for 3-D assembly.

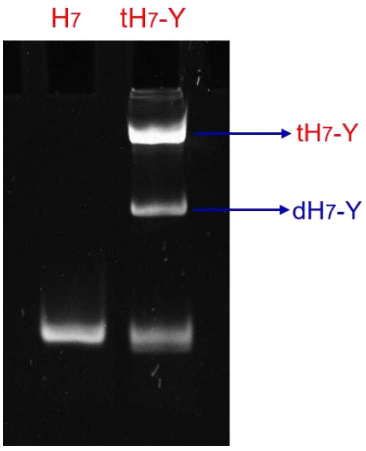

Figure S8. Non-denature PAGE gel electrophoresis of the triple AIEgen-DNA conjugates.

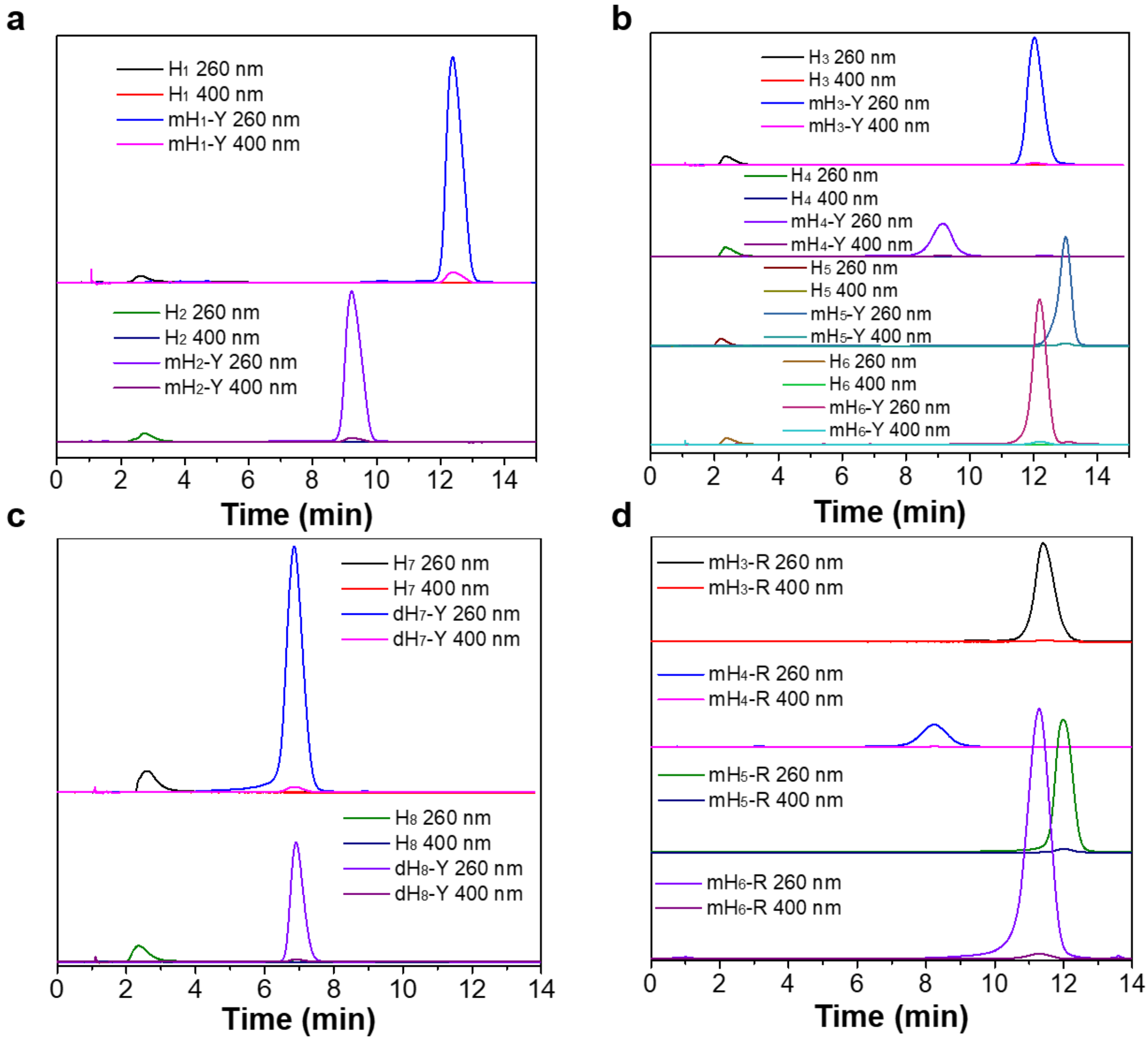

Figure S9. Reverse phase-HPLC spectra of (a) 1-D (mY-N_3)-DNA conjugates, (b) 2-D (mY-N_3)-DNA conjugates, (c) 3-D (dY-N_3)-DNA conjugates and (d) 2-D (mR-N_3)-DNA conjugates with absorbance at both 260 nm and 400 nm.

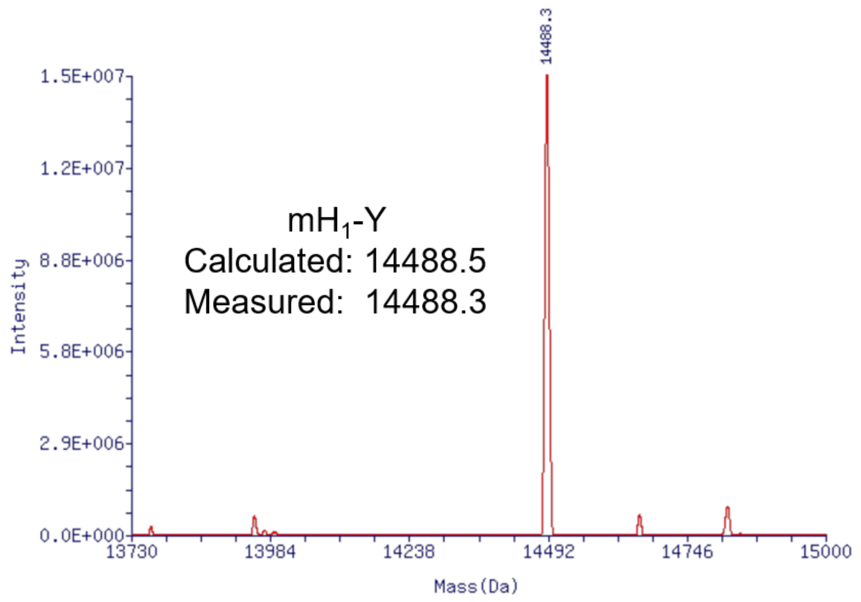
14488.3
1.5E+007
1.2E+007
8.8E+006
5.8E+006
2.9E+006
0.0E+000
Intensity
mH1-Y
Calculated: 14488.5
Measured: 14488.3
13730
13984
14238
14492
14746
15000
Mass(Da)

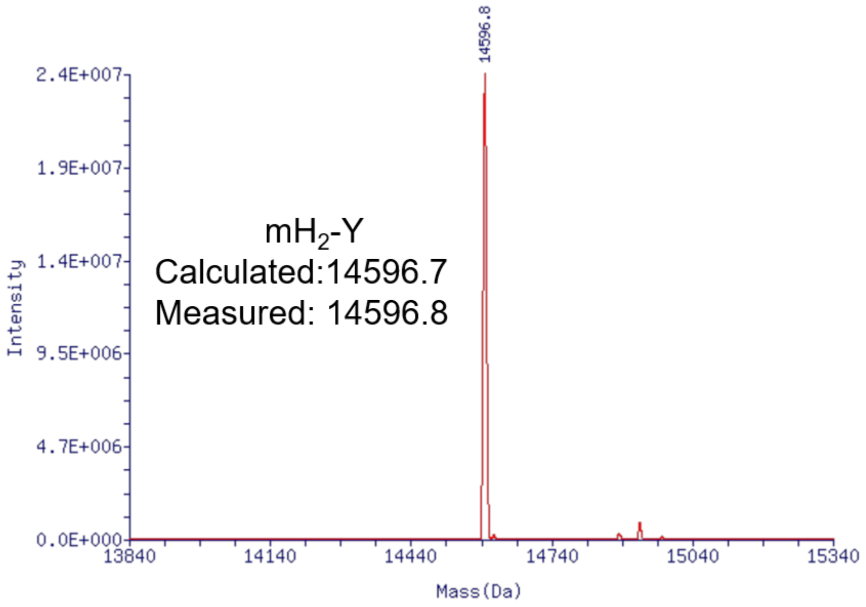
14596.8
2.4E+007
1.9E+007
1.4E+007
9.5E+006
4.7E+006
0.0E+000
Intensity
mH2-Y
Calculated:14596.7
Measured: 14596.8
13840
14140
14440
14740
15040
15340
Mass(Da)

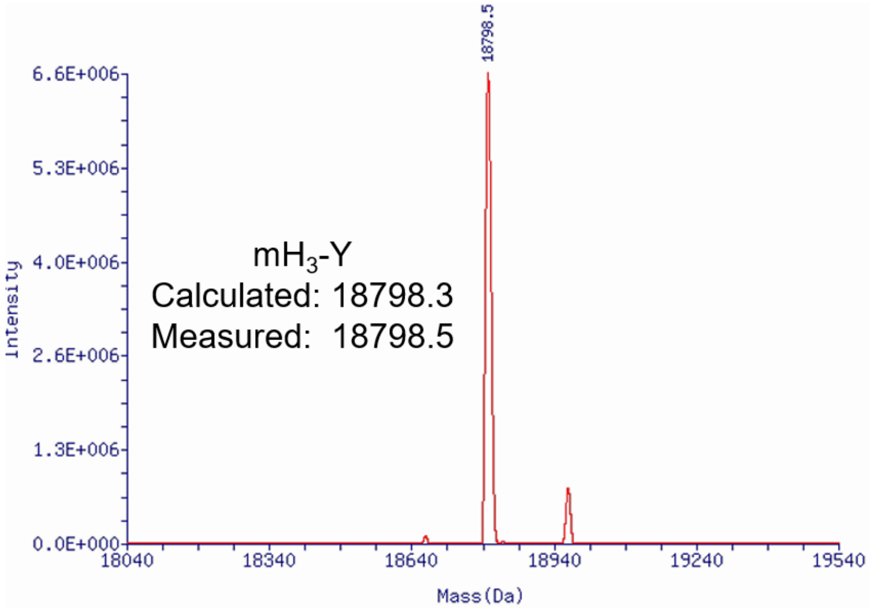
18798.5
6.6E+006
5.3E+006
4.0E+006
2.6E+006
1.3E+006
0.0E+000
Intensity
mH3-Y
Calculated: 18798.3
Measured: 18798.5
18040
18340
18640
18940
19240
19540
Mass(Da)

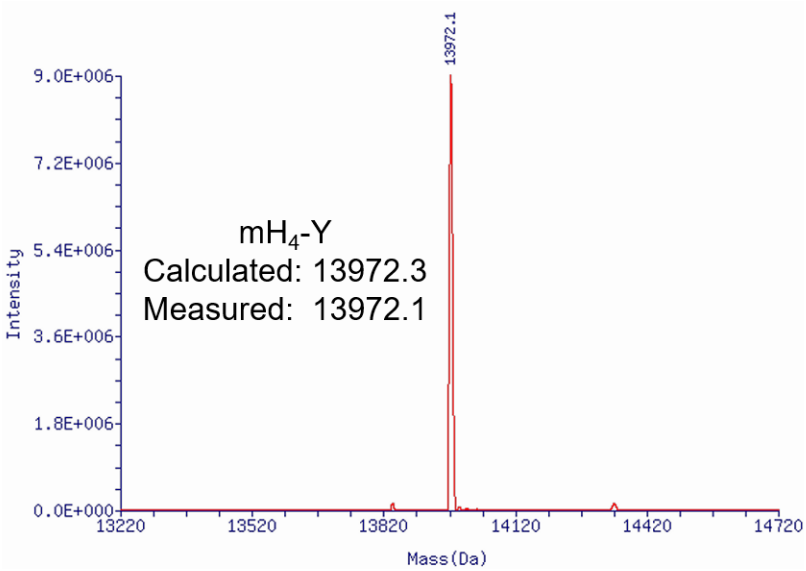
13972.1
9.0E+006
7.2E+006
5.4E+006
3.6E+006
1.8E+006
0.0E+000
Intensity
mH4-Y
Calculated: 13972.3
Measured: 13972.1
13220
13520
13820
14120
14420
14720
Mass(Da)

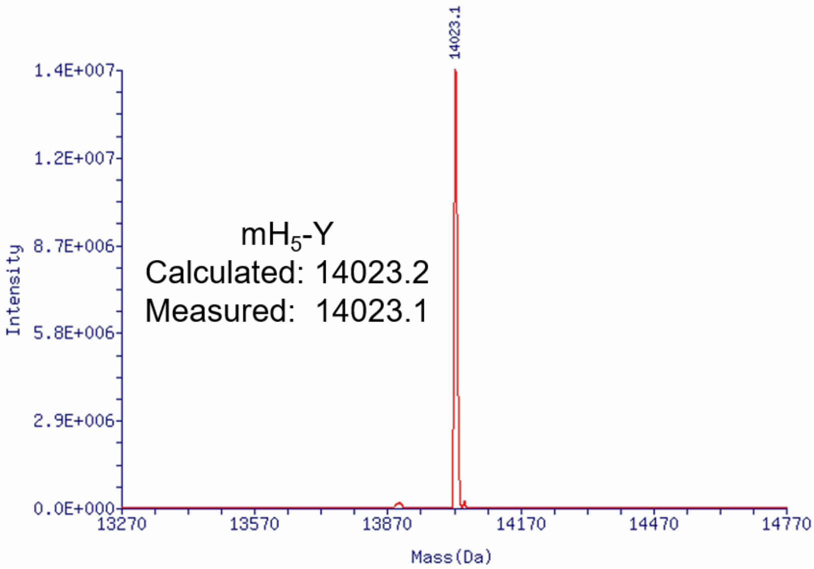
14023.1
1.4E+007
1.2E+007
8.7E+006
5.8E+006
2.9E+006
0.0E+000
Intensity
mH5-Y
Calculated: 14023.2
Measured: 14023.1
13270
13570
13870
14170
14470
14770
Mass(Da)

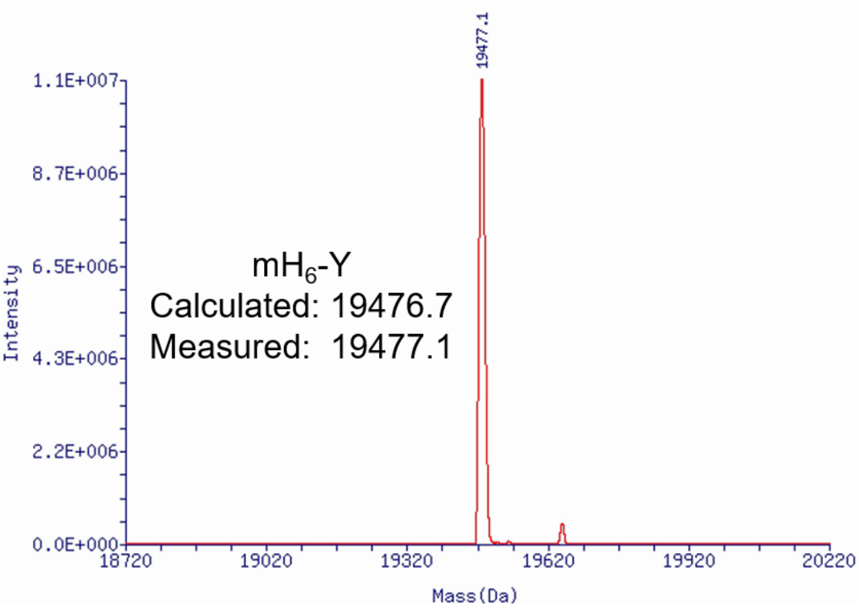
19477.1
1.1E+007
8.7E+006
6.5E+006
4.3E+006
2.2E+006
0.0E+000
Intensity
mH6-Y
Calculated: 19476.7
Measured: 19477.1
18720
19020
19320
19620
19920
20220
Mass(Da)

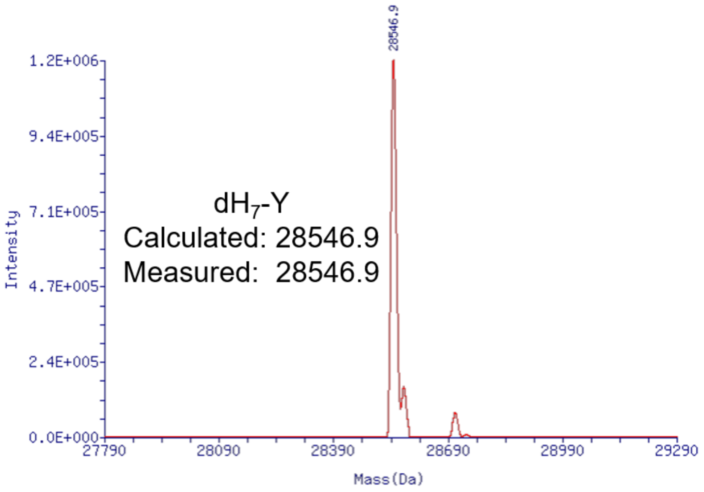
28546.9
1.2E+006
9.4E+005
7.1E+005
4.7E+005
2.4E+005
0.0E+000
Intensity
dH$_7$-Y
Calculated: 28546.9
Measured: 28546.9
27790
28090
28390
28690
28990
29290
Mass(Da)

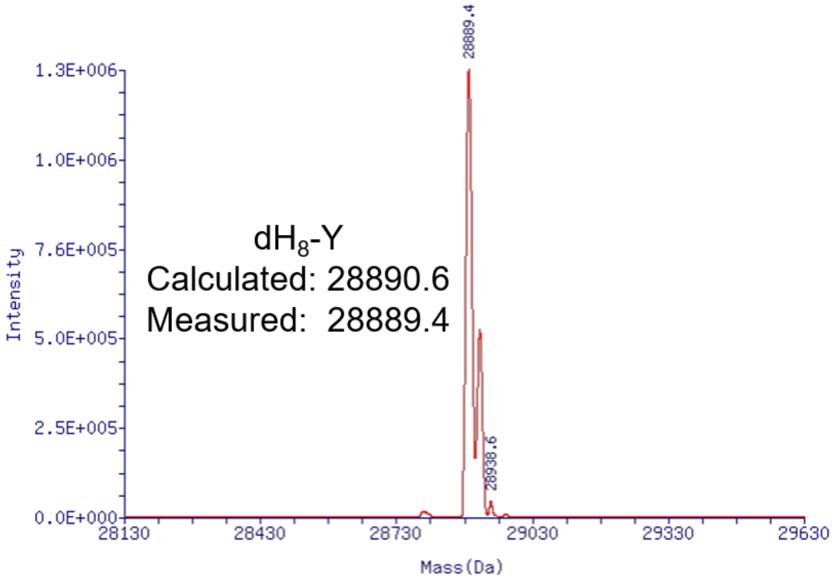
28889.4
1.3E+006
1.0E+006
7.6E+005
5.0E+005
2.5E+005
0.0E+000
Intensity
dH$_8$-Y
Calculated: 28890.6
Measured: 28889.4
28938.6
28130
28430
28730
29030
29330
29630
Mass(Da)

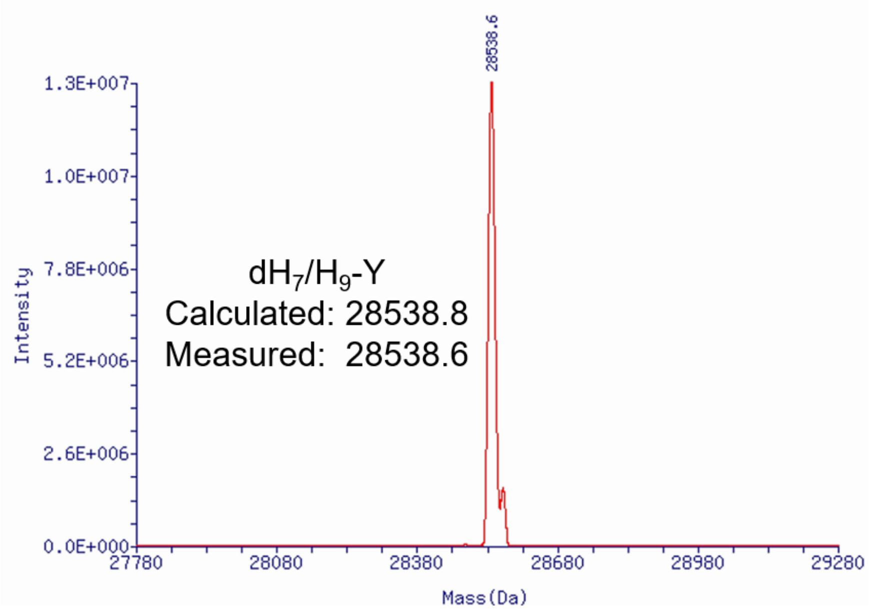
28538.6
1.3E+007
1.0E+007
7.8E+006
5.2E+006
2.6E+006
0.0E+000
Intensity
dH$_7$/H$_9$-Y
Calculated: 28538.8
Measured: 28538.6
27780
28080
28380
28680
28980
29280
Mass(Da)

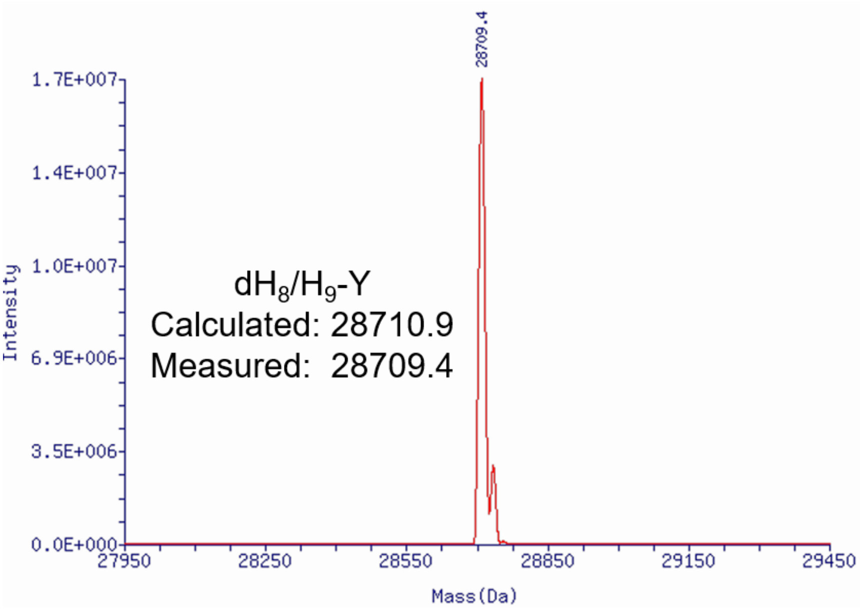
28709.4
1.7E+007
1.4E+007
1.0E+007
6.9E+006
3.5E+006
0.0E+000
Intensity
dH_8/H_9-Y
Calculated: 28710.9
Measured: 28709.4
27950
28250
28550
28850
29150
29450
Mass(Da)

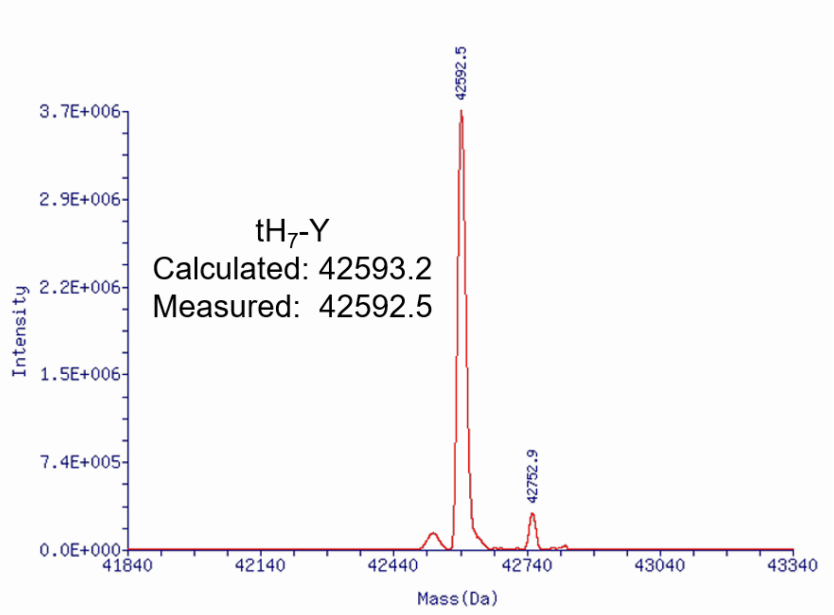
42592.5
42752.9
3.7E+006
2.9E+006
2.2E+006
1.5E+006
7.4E+005
0.0E+000
Intensity
tH_7-Y
Calculated: 42593.2
Measured: 42592.5
41840
42140
42440
42740
43040
43340
Mass(Da)

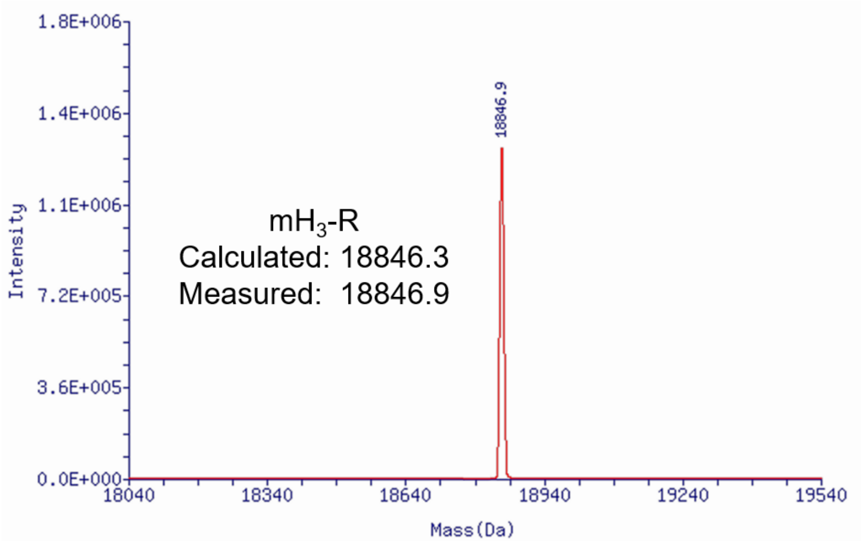
18846.9
1.8E+006
1.4E+006
1.1E+006
7.2E+005
3.6E+005
0.0E+000
Intensity
mH_3-R
Calculated: 18846.3
Measured: 18846.9
18040
18340
18640
18940
19240
19540
Mass(Da)

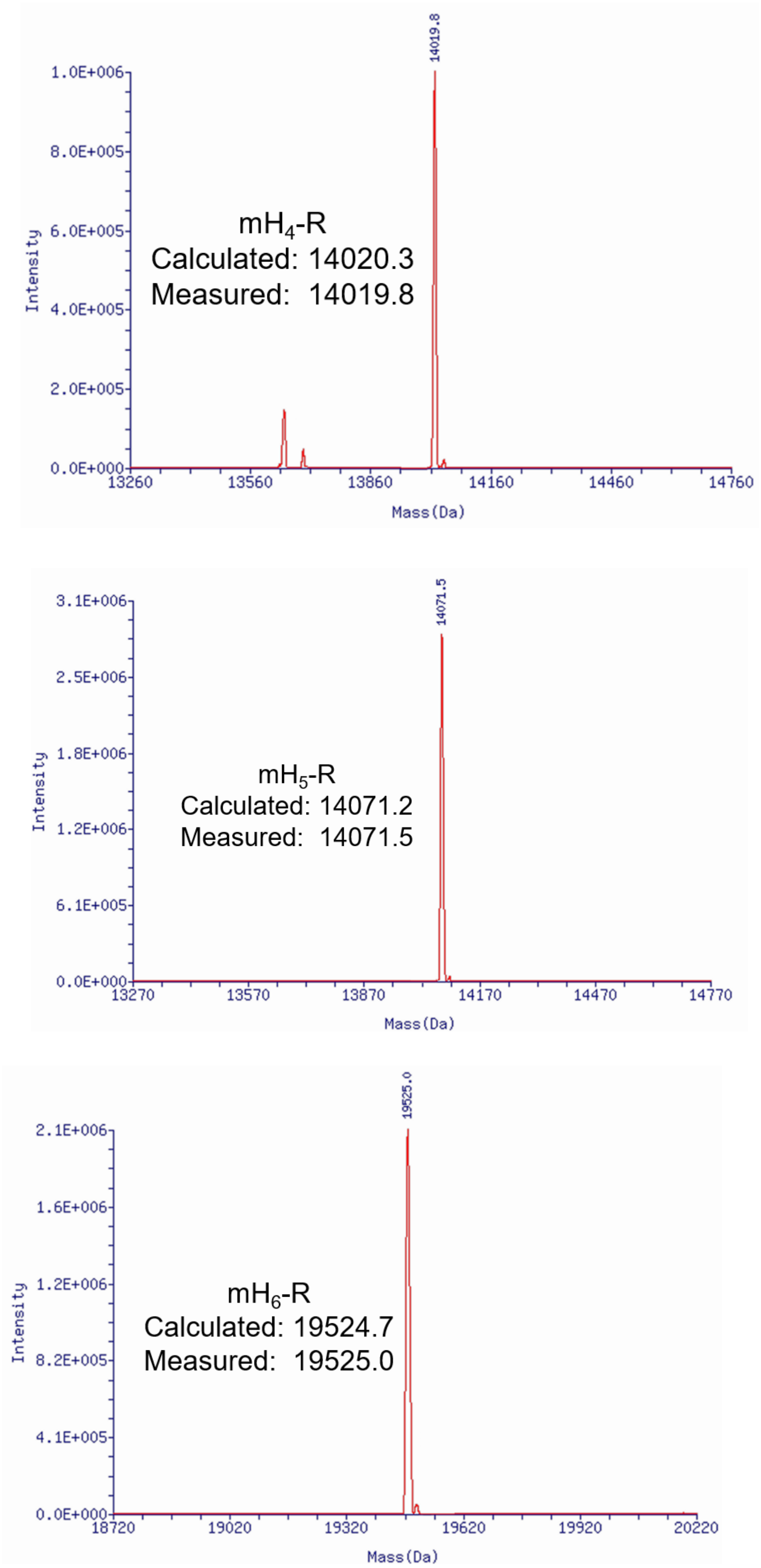

Figure S10. Mass spectrums of various AIEgen-DNA conjugates by Thermo LTQ (model).

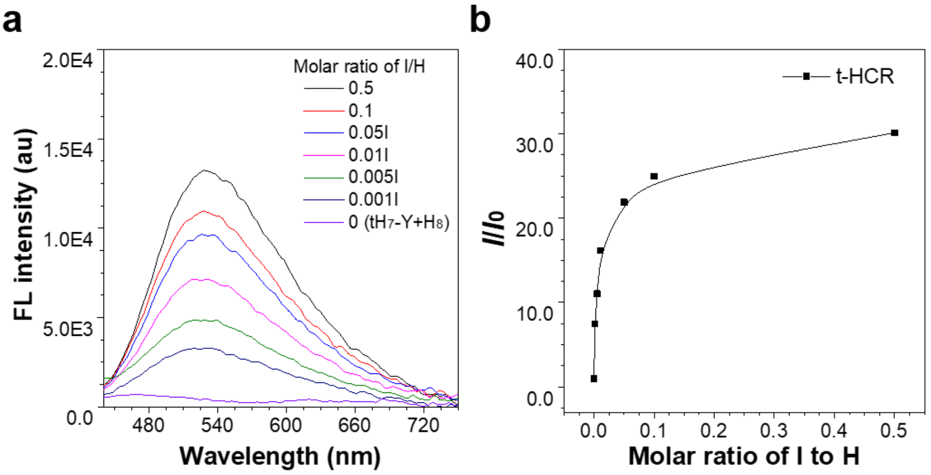

Figure S11. (a) Fluorescnece spectra of the 3-D assembly were from AIEgen-conjugated DNA tH_7-Y or nude DNA hairpins H_8 via HCR reactions. (b) Plotting of the fluorescence intensity at maximum of the produced 3-D assemblies versus the molar ratio of I/H.

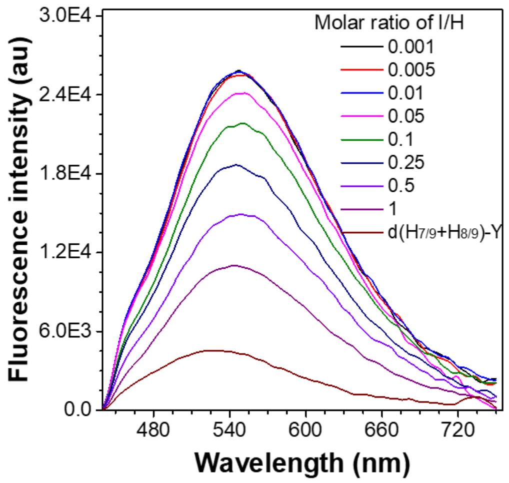

Figure S12. Fluorescnece spectra of the HCR assembly of $d(H_7/H_9)$-Y and $d(H_8/H_9)$-Y conjugates.

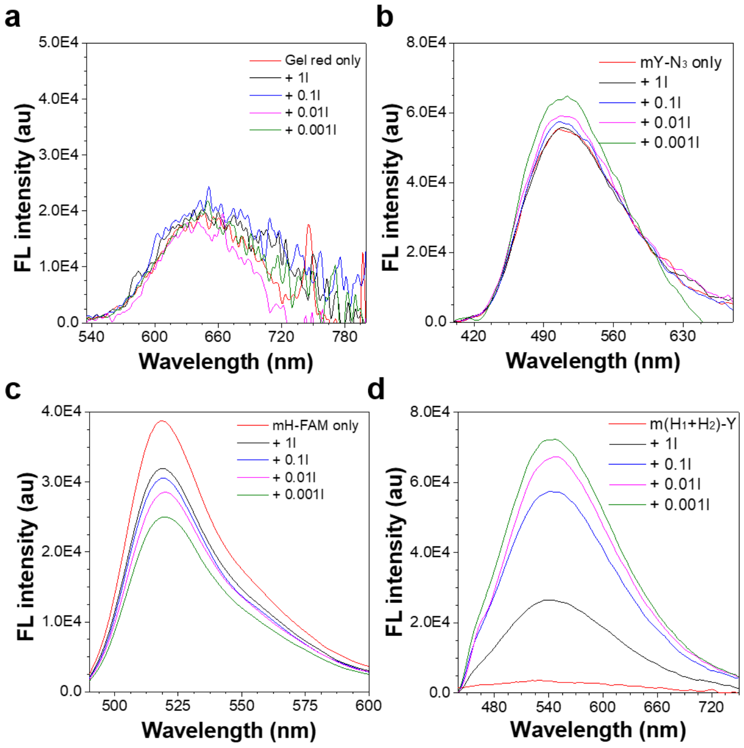

Figure S13. Fluorescnece spectra of (a) Gel red, (b) mY-N_3, (c) H_1 and H_2 modified FAM and (d) mH-Y before and after adding 1-fold, 0.1-fold, 0.01-fold, 0.001-fold Initiator.

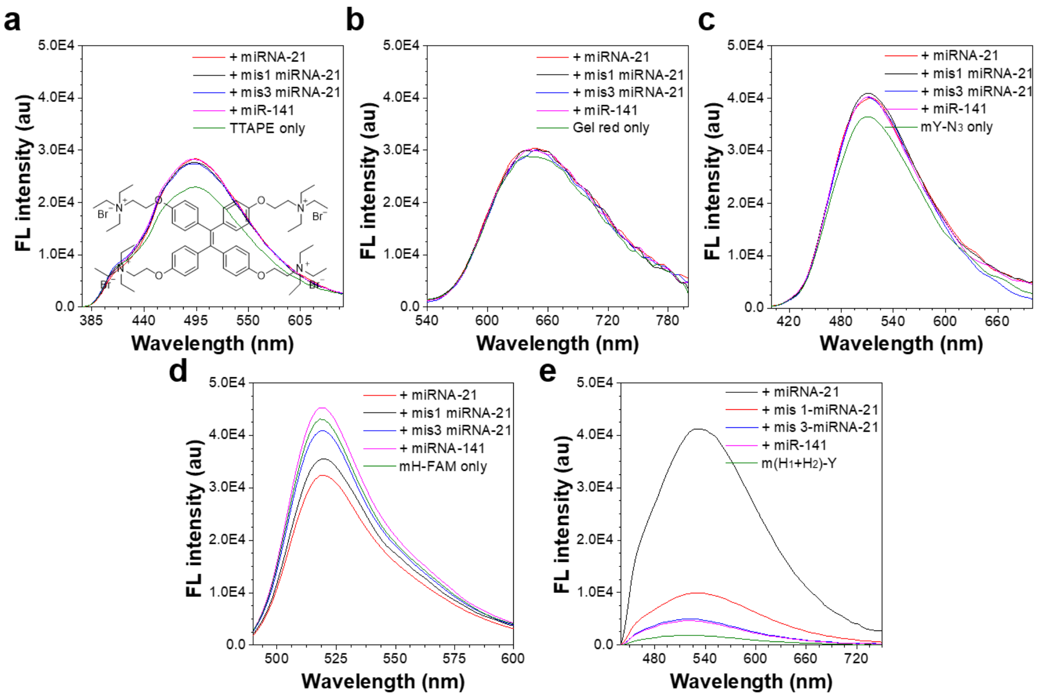

Figure S14. Fluorescnece spectra of the selectivity of (a) TTAPE which can recognize nucleic acid, (b) Gel red, (c) mY-N_3, (d) H_1 and H_2 modified FAM, and mH-Y with non-complementary target (miR-141), target RNA with one or three mismatched bases, and target miRNA-21 before and after adding 0.001-fold Initiator.

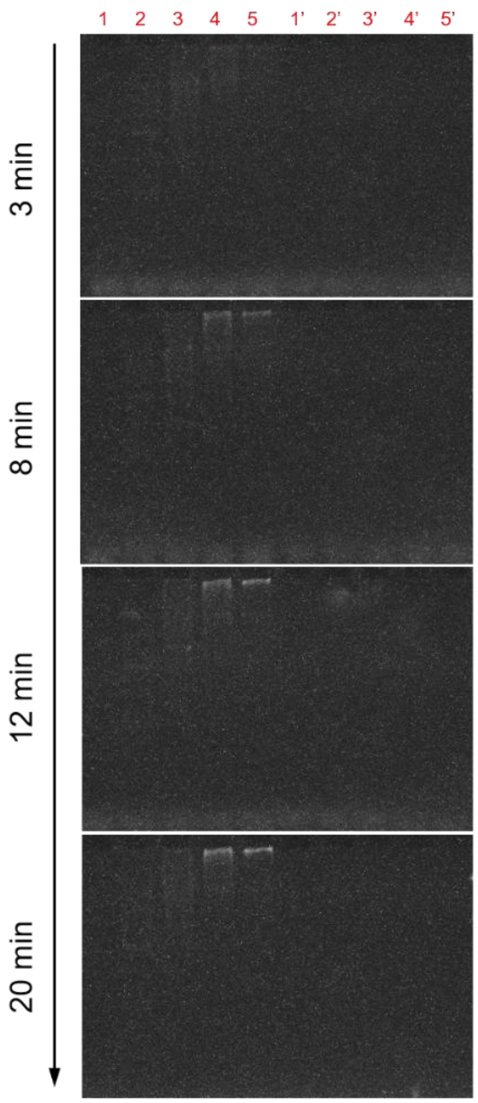

1-5 (DNA-AIEgen) : mH_1-Y、1I、0.5I、0.1I、0.01I; 1’-5’ (DNA only) : H_1、1I、0.5I、0.1I、0.01I

Figure S15. Monitoring of the HCR reaction process via non-denature PAGE gel electrophoresis. The produced 1-D assemblies were from AIEgen-DNA conjugates or nude DNA hairpins after reaction for 3 min to 20 min. The FL signal was originated from the assembled AIEgens with HCR reaction.

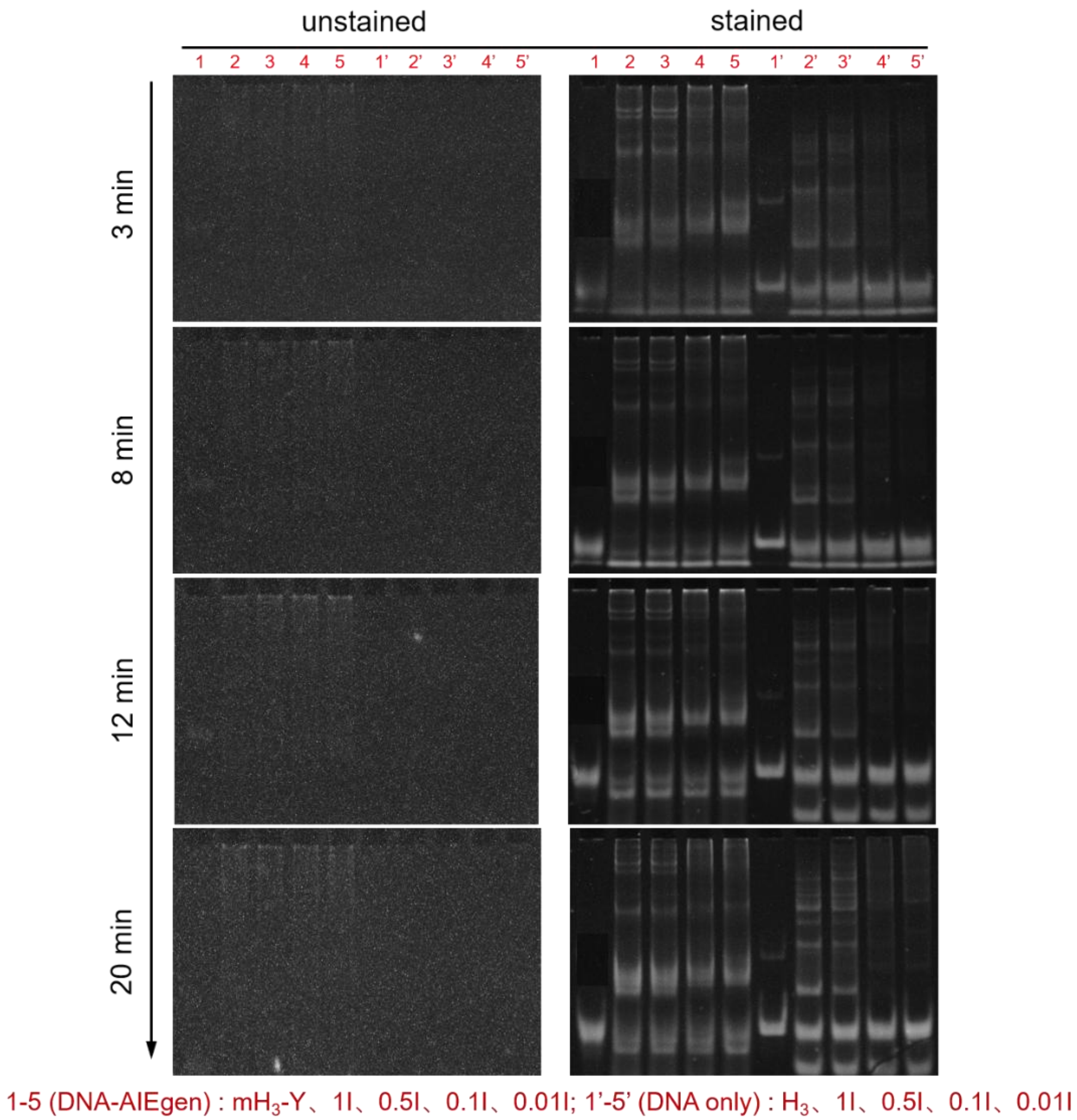

Figure S16. Monitoring of the HCR reaction process via non-denature PAGE gel electrophoresis before and after 4SGelred dye staining. The produced 2-D assemblies were from AIEgen-DNA conjugates or nude DNA hairpins after reaction for 3 min to 20 min.

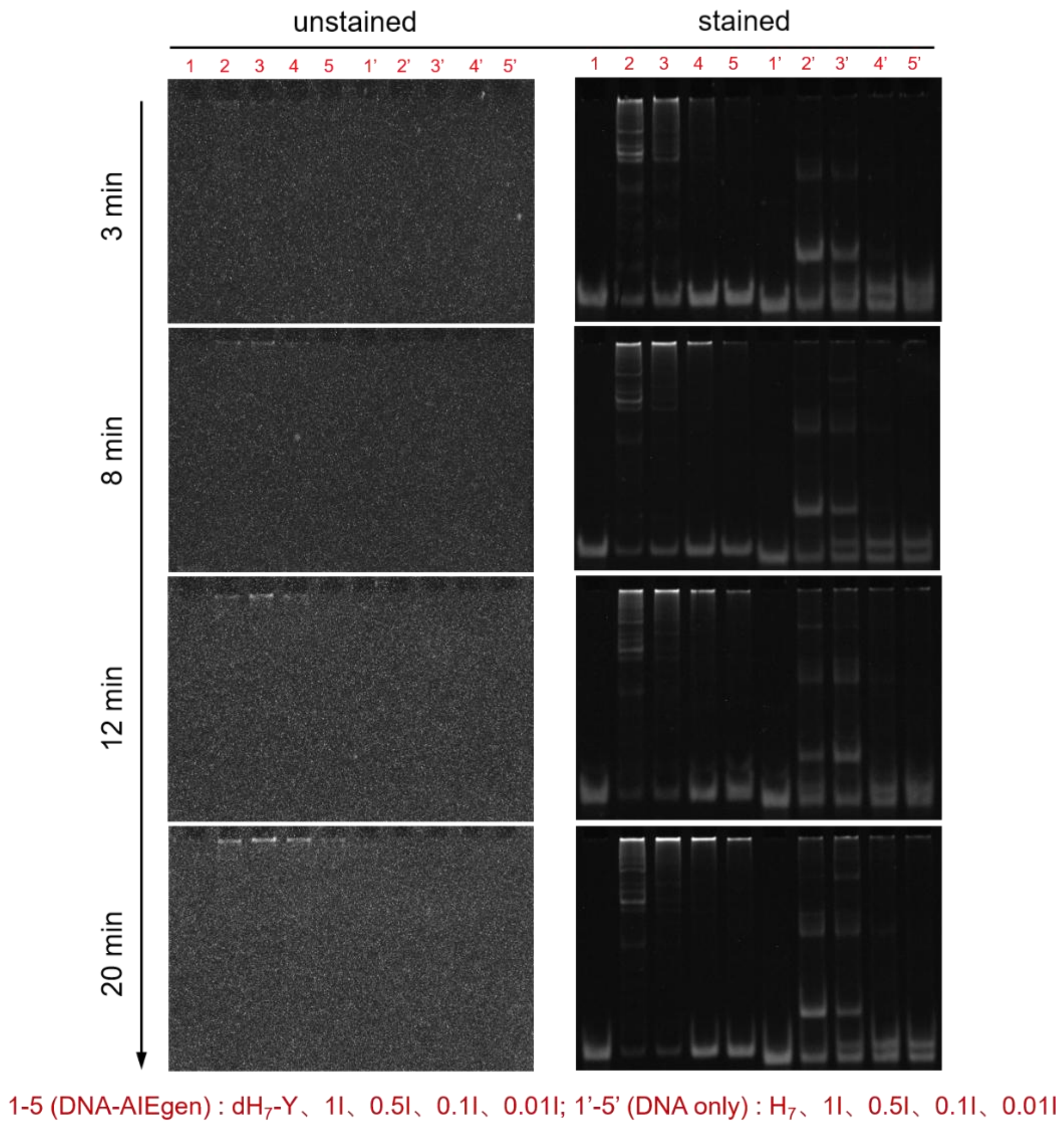

Figure S17. Monitoring of the HCR reaction process via non-denature PAGE gel electrophoresis before and after 4SGelred dye staining. The produced 3-D assemblies were from AIEgen-DNA conjugates or nude DNA hairpins after reaction time for 3 min to 20 min.

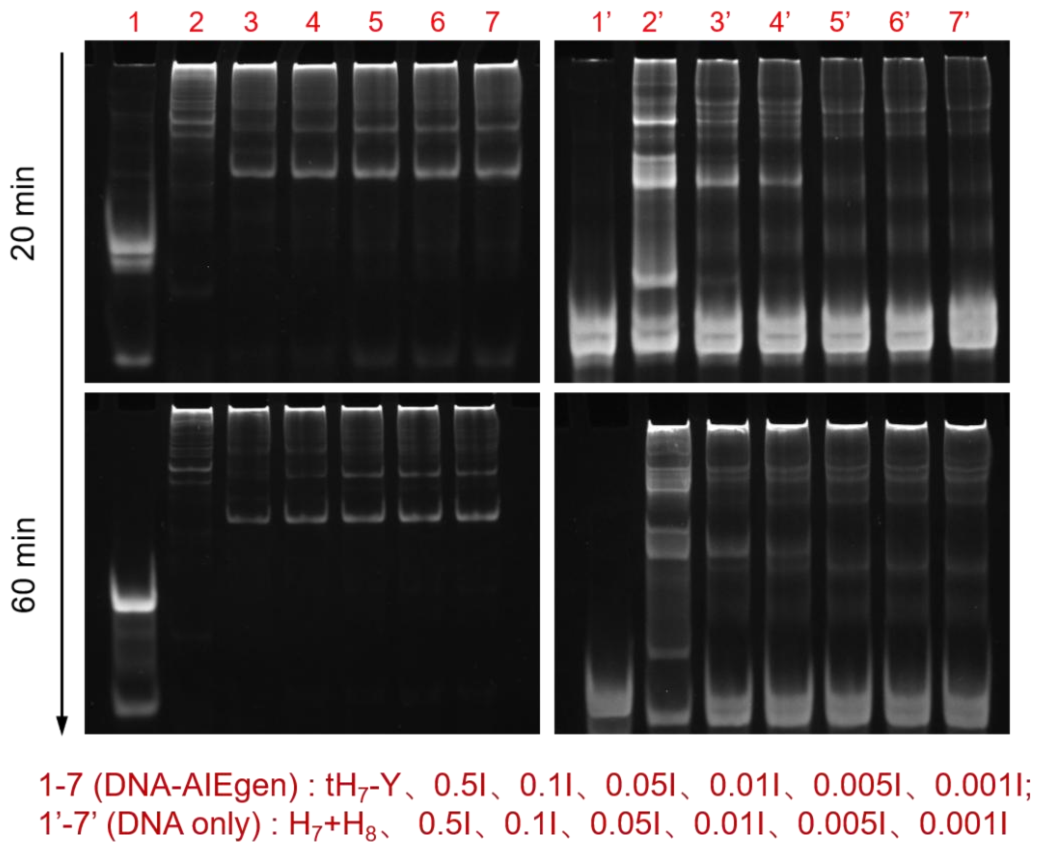

Figure S18. Monitoring of the HCR reaction process via non-denature PAGE gel electrophoresis after 4SGelred dye staining. The produced assemblies were from AIEgen-DNA conjugate tH_7-Y or nude DNA hairpins H_8 after reaction time for 20 min to 60 min.

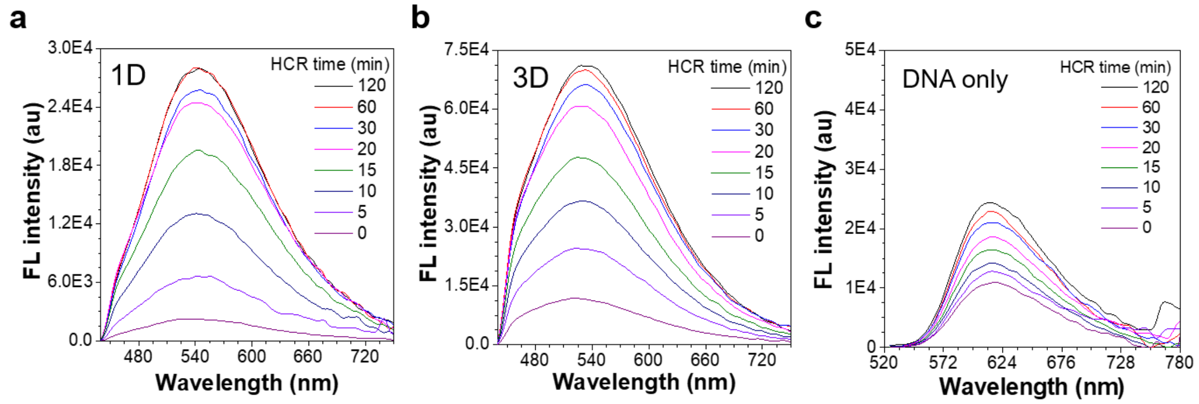

Figure S19. Fluorescnece spectra of the (a) 1-D, (b) 3-D assembly of AIEgen-DNA conjugates and (c) 1D assembly of nude DNA after 4SGelred staining via HCR reactions with various time excited by 365 nm laser.

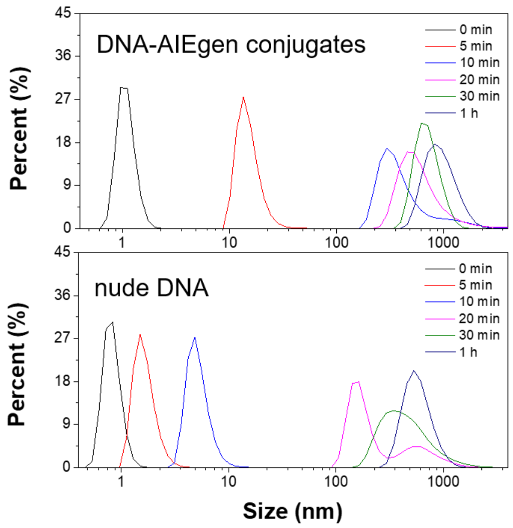

Figure S20. Monitoring of the HCR reaction process via dynamic light scattering measurements of the hydrodynamic sizes of produced 1-D assemblies from free DNA hairpins or AIEgen-DNA conjugates along with the reaction time from 3 min to 60 min.

Table 1 Comparison of reaction rates among different HCR systems

HCR assembly systems	Reaction time	Reference
AIEgens-DNA conjugate HCR assembly	20 min	**This work**
Nude HCR	1440 min	[S1]
DNA hairpins with mismatch	240 min	[S2]
DNA-templated magnetic nanoparticle-quantum dot -aptamer copolymers	720 min	[S3]
Antibody-HCR conjugation	30-60 min	[S4]
Electrostatic DNA nanoassembly	180 min	[S5]
Ligation-mediated branched HCR	240 min	[S6]
Self-assembly of patterned DNA hydrogels	720 min	[S7]
Triangular DNA origami nanostructure-HCR	30 min	[S8]
Computer-aided design of reversible HCR	30 min	[S9]
Proximity-induced HCR	1440 min	[S10]
Branched HCR	180 min	[S11]
Switch-engineered spherical nucleic acid-templated hydrogel	10-60 min	[S12]

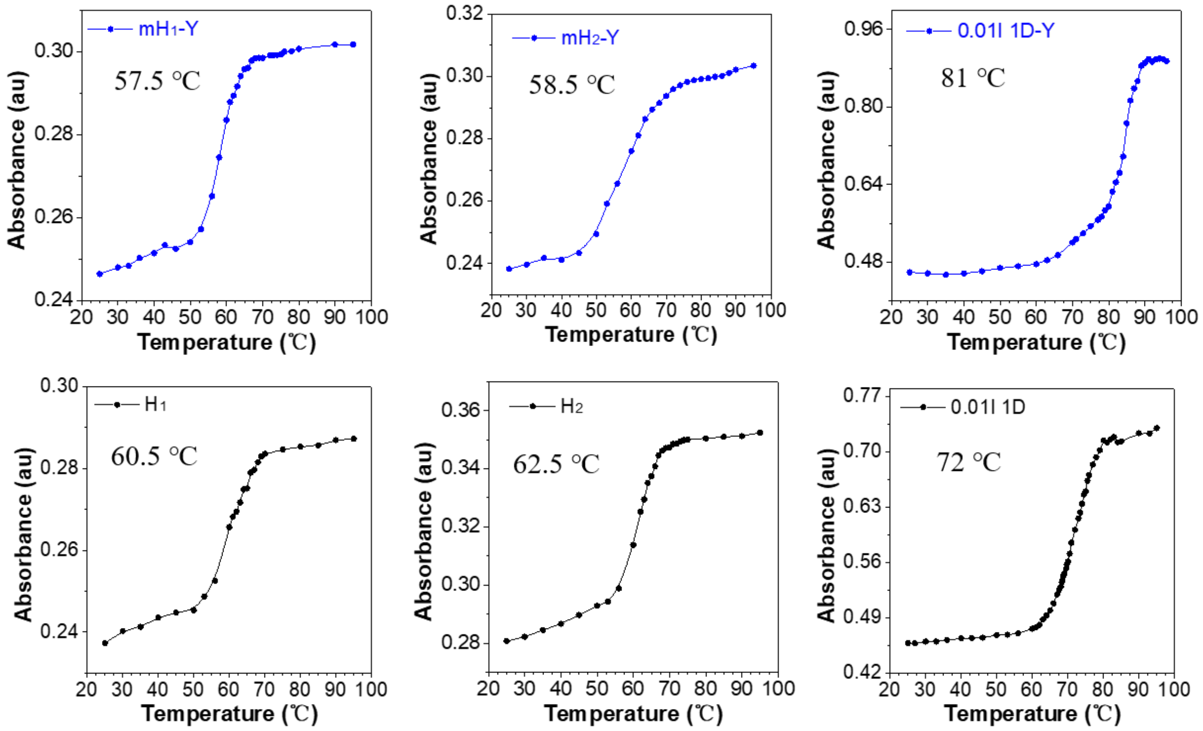

Figure S21. Melting temperature testing of H_1, H_2, and 1-D HCR assemblies (triggered by 0.01-fold Initiator) with or without AIEgen conjugation via UV—Vis spectra.

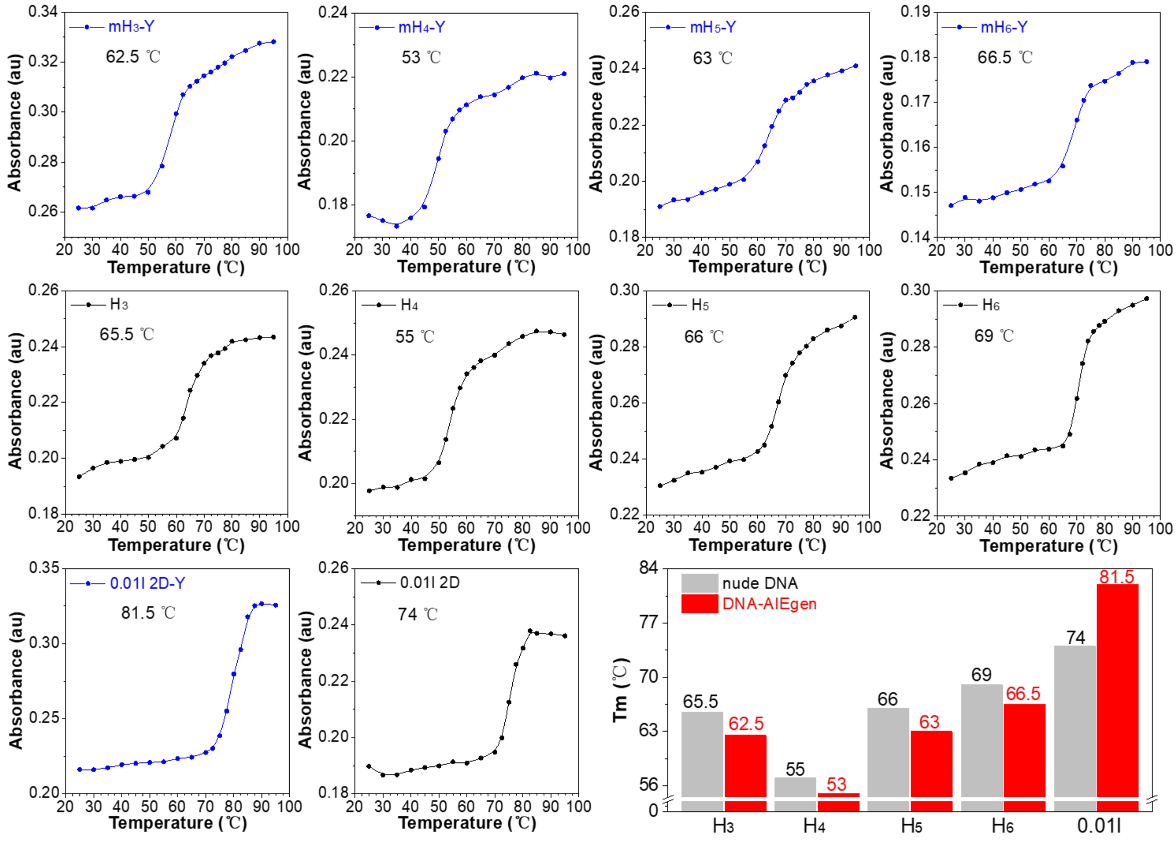

Figure S22. Melting temperature testing of H_3, H_4, H_5, H_6, and 2-D HCR assemblies (triggered by 0.001-fold Initiator) with or without AIEgen conjugation via UV—Vis spectra.

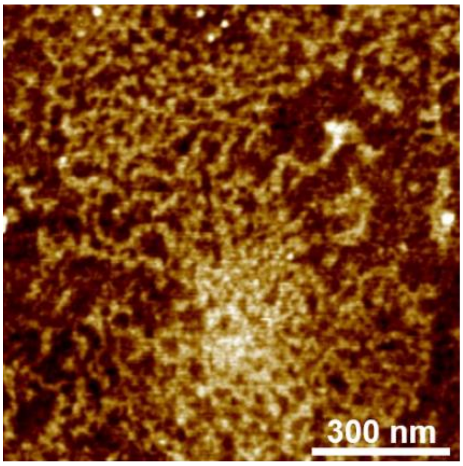

Figure S23. AFM imaging of the controlled assembly via AIEgen-DNA conjugate (tH_7-Y) and nude DNA H_8. The 0.1-fold Initiator was used as the trigger of HCR reaction.

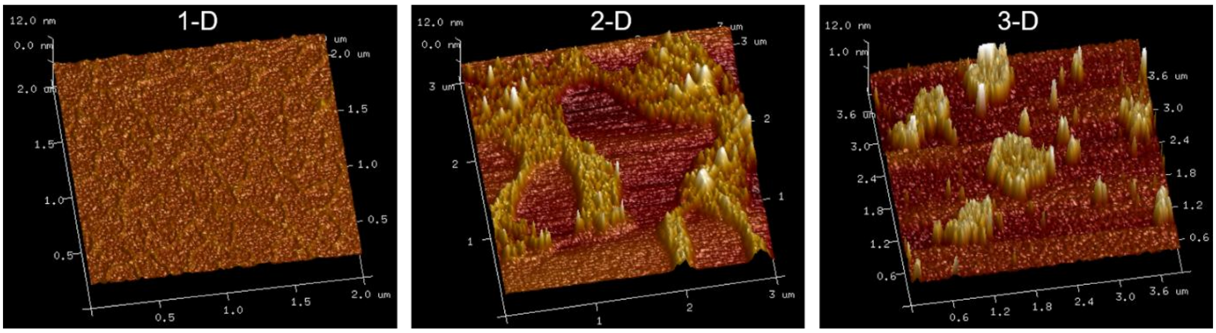

Figure S24. Three-dimensional AFM imaging of the 1-D, 2-D and 3-D controlled assembly via AIEgen-DNA conjugates. The 0.1-fold Initiator was used as the trigger of HCR reaction. The depth of the 1-D, 2-D and 3-D assembly was 0 nm, 15 nm and 50 nm, respectively.

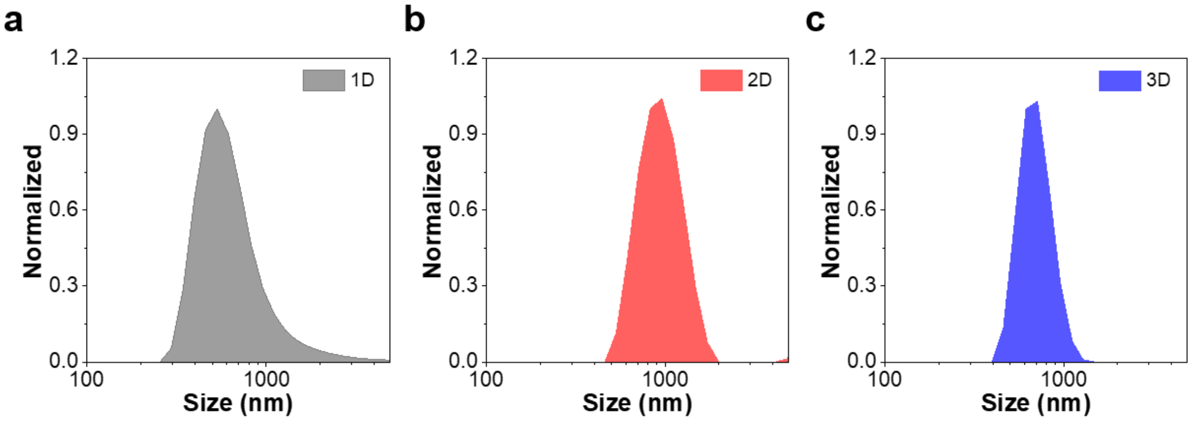

Figure S25. Dynamic light scattering measurements of the hydrodynamic sizes of (a-c) produced 1-D, 2-D and 3-D assemblies, respectively.

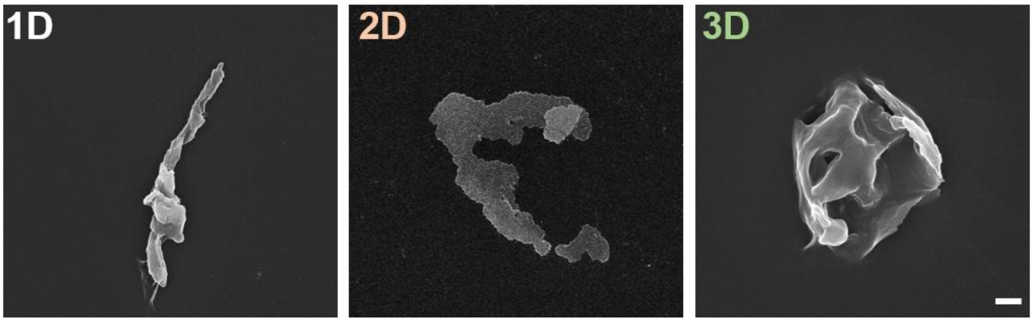

Figure S26. SEM imaging of the controlled assembly AIEgen-DNA conjugates for linear 1-D, dendritic 2-D and stereo 3-D structures. The 0.001-fold (1-D and 2-D) and 0.1-fold (3-D) Initiator was used as the trigger of HCR reaction. The scale bars were 500 nm.

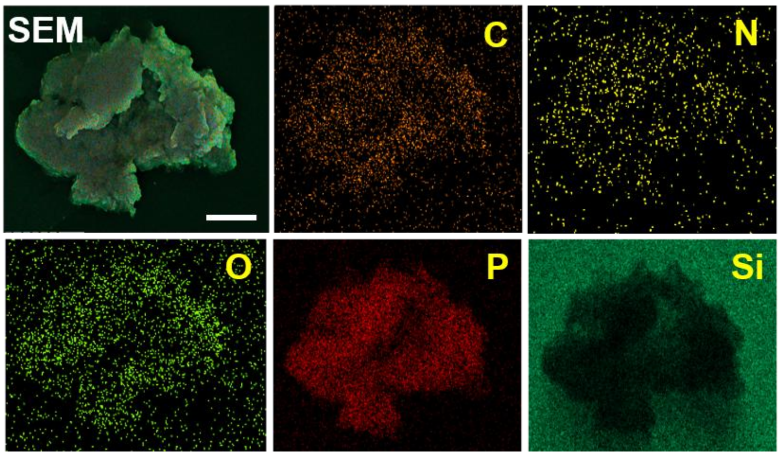

Figure S27. SEM image and EDS mapping of C, N, O, P and Si elements of 3-D HCR assembly. The scale bars were 500 nm.

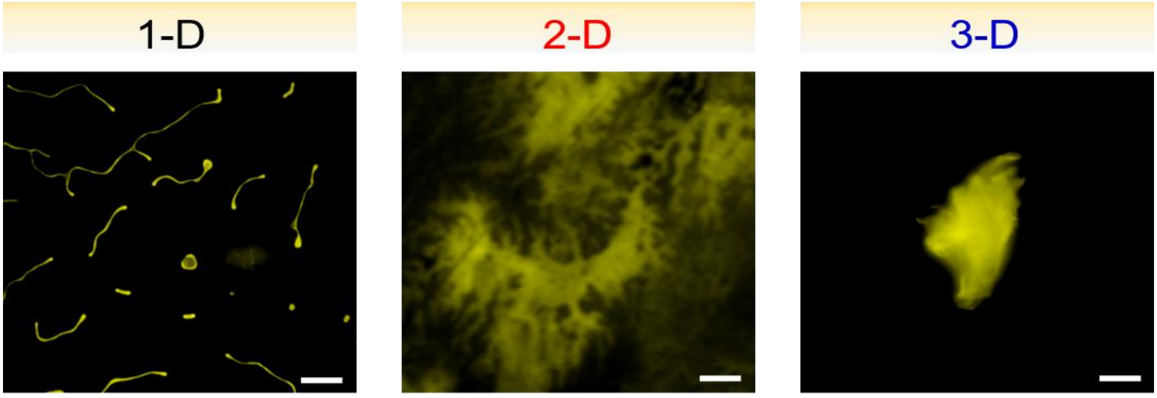

Figure S28. Morphological characterization of the controlled assembly AIEgen-DNA- for linear 1-D, dendritic 2-D and stereo 3-D structures via fluorescence microscopic imaging. The 0.001-fold (1-D and 2-D) and 0.1-fold (3-D) Initiator was used as the trigger of the HCR reaction. All the scale bars were 500 nm.

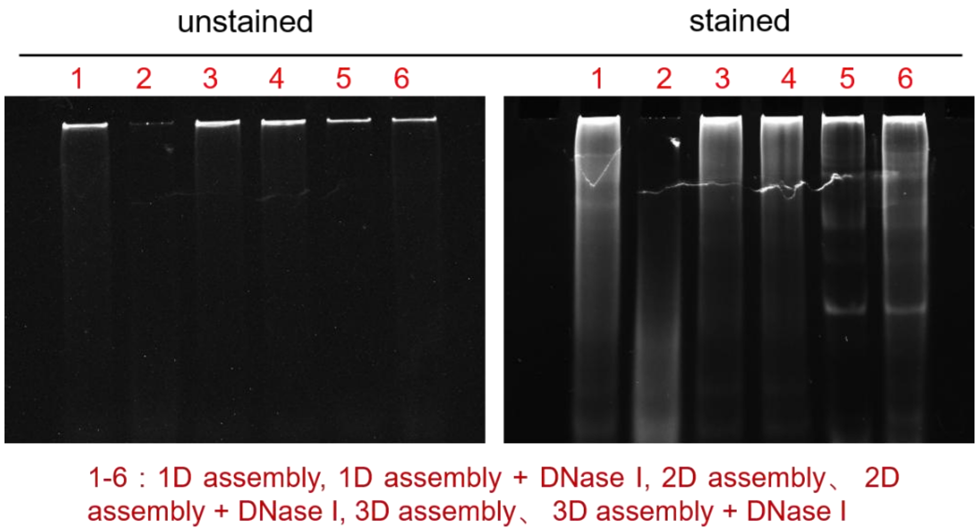

Figure S29. Non-denature PAGE gel electrophoresis of produced 1-D, 2-D and 3-D AIEgen-DNA conjugates assemblies via HCR reactions before and after incubation with 50 U/L DNase I for 12 h.

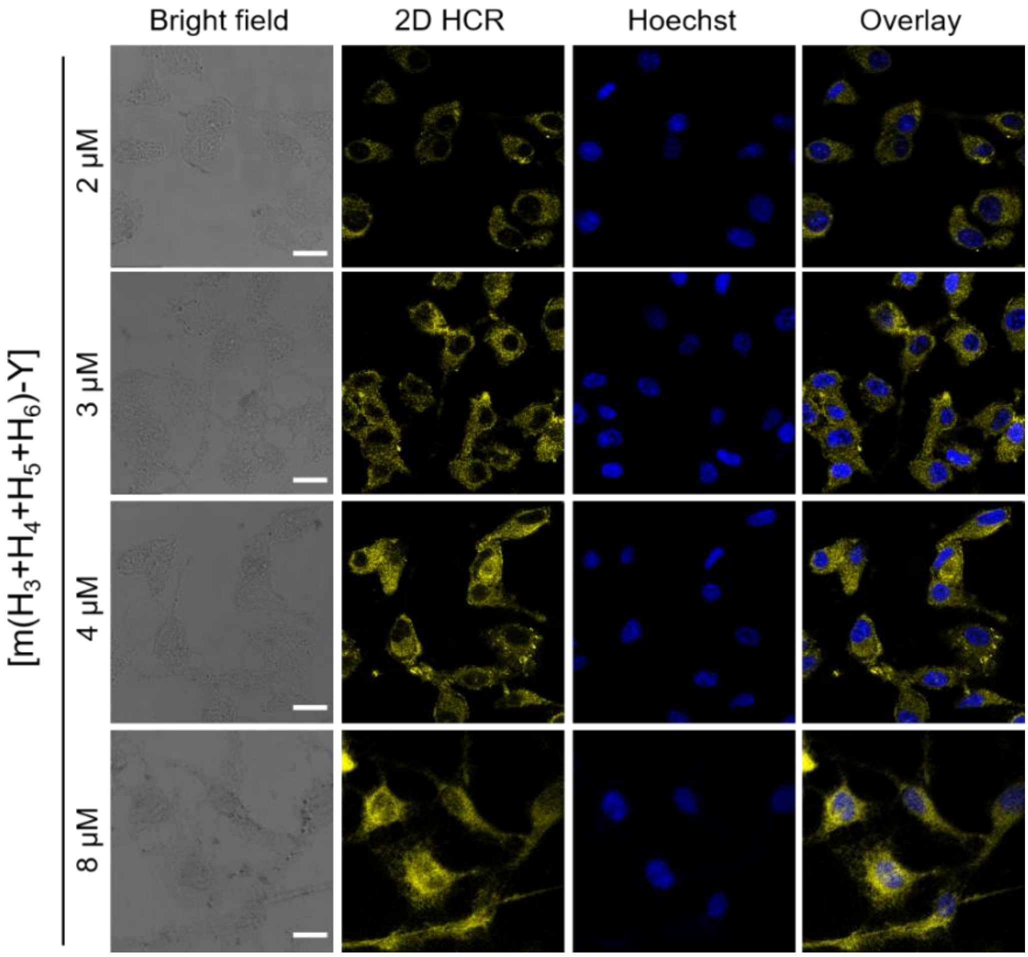

Figure S30. Confocal laser scanning imaging of HeLa cells after incubation with various

concentration of 2-D AIEgen-DNA hairpin conjugates for 4 h. Yellow channel for mY-N_3: ex 405 nm, em 530-600 nm; Blue channel for Hoechst: ex 405 nm, em 430-470 nm. All the scale bars were 20 μm.

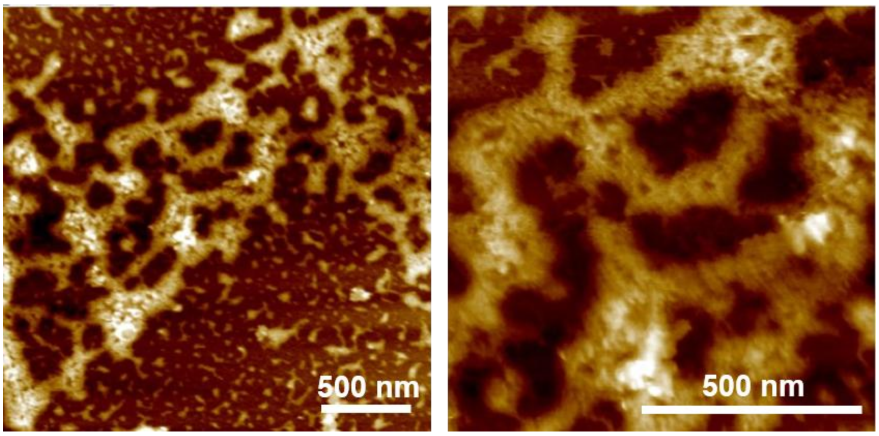

Figure S31. AFM imaging of HCR assembly AIEgen-DNA for 2-D dendritic structures extracted from cell lysate with different magnification.

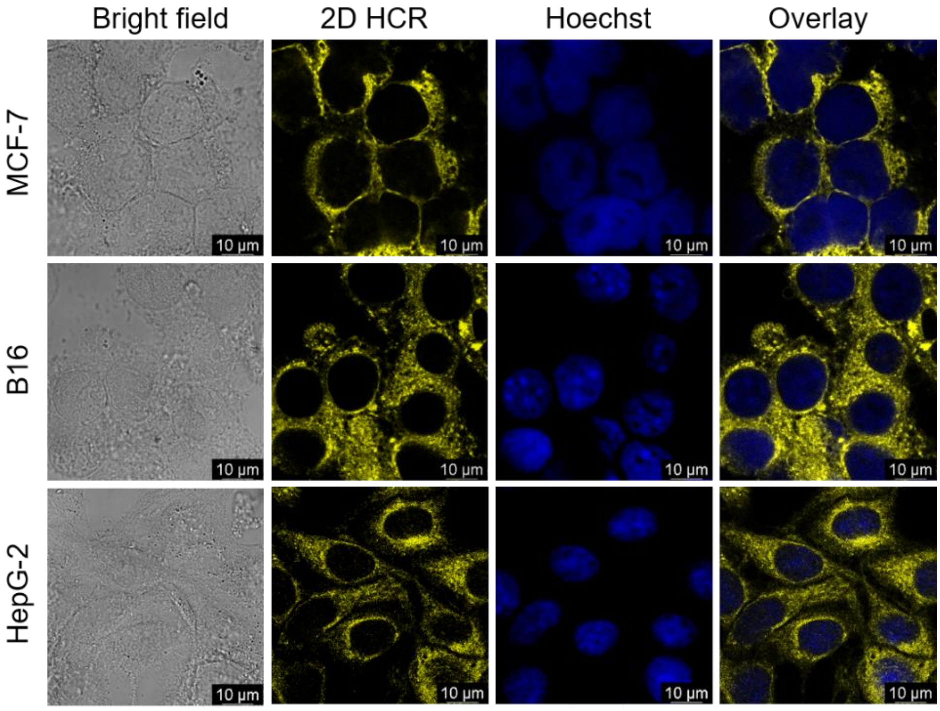

Figure S32. Confocal laser scanning imaging of MCF-7, B16 and HepG-2 cells after incubation with 2-D AIEgen-DNA hairpin conjugates for 4 h. Yellow channel for mY-N_3: ex 405 nm, em 530-600 nm; Blue channel for Hoechst: ex 405 nm, em 430-470 nm.

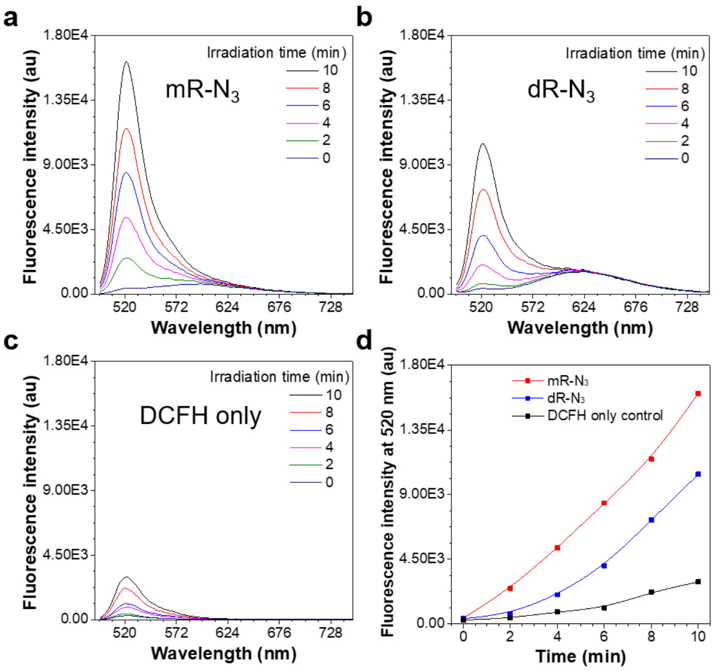

Figure S33. Chemical trapping of total ROS generation. Photoactivation of DCFH mixed with (a) mR-N_3 aggregates, (b) dR-N_3 aggregates and (c) DCFH control under white light irradiation, respectively. (d) Plotting of the fluorescence intensity of the DCFH indicator versus the irradiation time in different groups. The power of white light power was 2 mW•cm^{-2}.

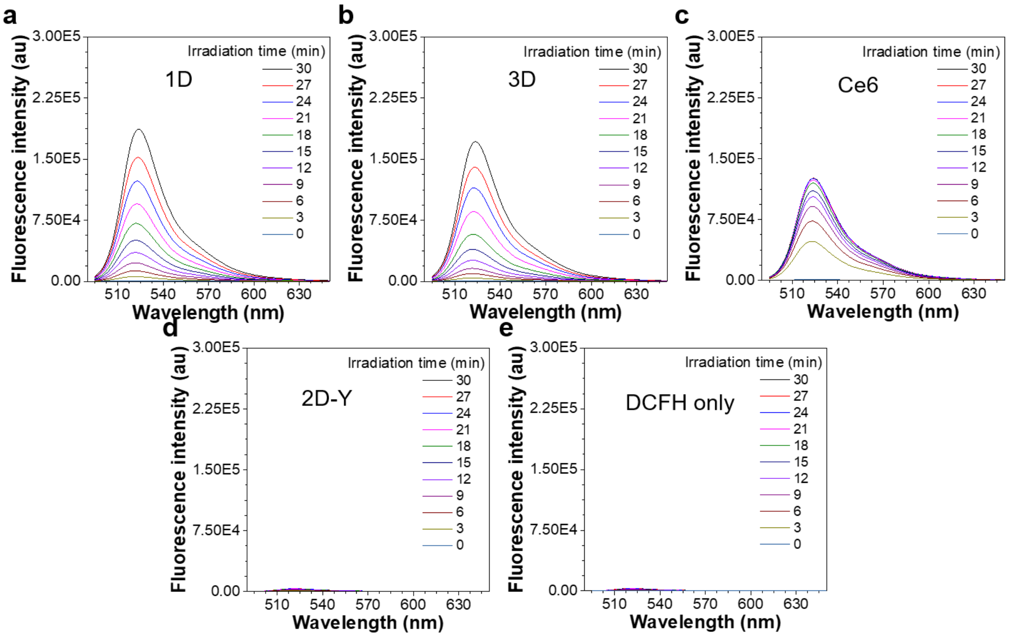

Figure S34. ROS generation efficiencies of (a) 1-D assemblies via Initiator + mH_1-R + mH_2-R, (b) 3-D assemblies via Initiator + dH_7-R + dH_8-R, (c) Ce6, (d) 2-D assemblies via Initiator + mH_3-Y + mH_4-Y + mH_5-Y + mH_6-Y and (e) DCFH control under white light irradiation, respectively. (f) Plotting of the fluorescence intensity of DCFH indicator versus

the irradiation time in different groups. The power of white light power was 2 mW•cm^{-2}.

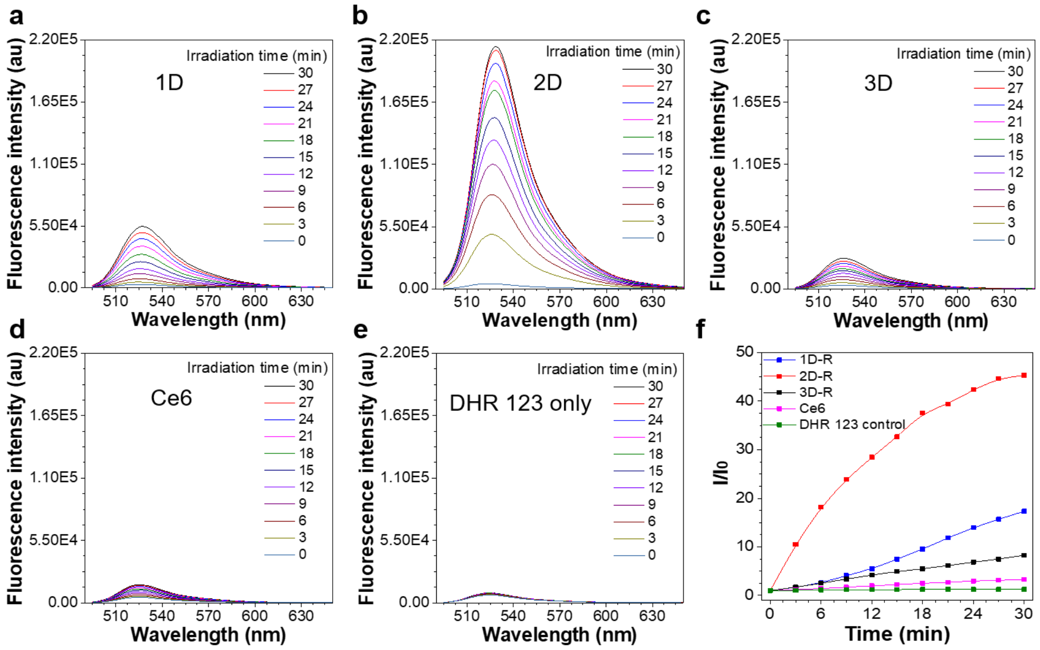

Figure S35. Chemical trapping of the $O_2^{\bullet-}$ generation. Photoactivation of DHR with (a) 1-D assemblies via Initiator + mH$_1$-R + mH$_2$-R, (b) 2-D assemblies via Initiator + mH$_3$-R + mH$_4$-R + mH$_5$-R + mH$_6$-R, (c) 3-D assemblies via Initiator + dH$_7$-R + dH$_8$-R, (d) Ce6 and (e) DHR 123 control under white light irradiation, respectively. (f) Activation rates of DHR 123 monitoring at 524 nm emission. [DHR] = 1 $\times 10^{-5}$ M, [Ce6] = 0.5 $\times 10^{-5}$ M.

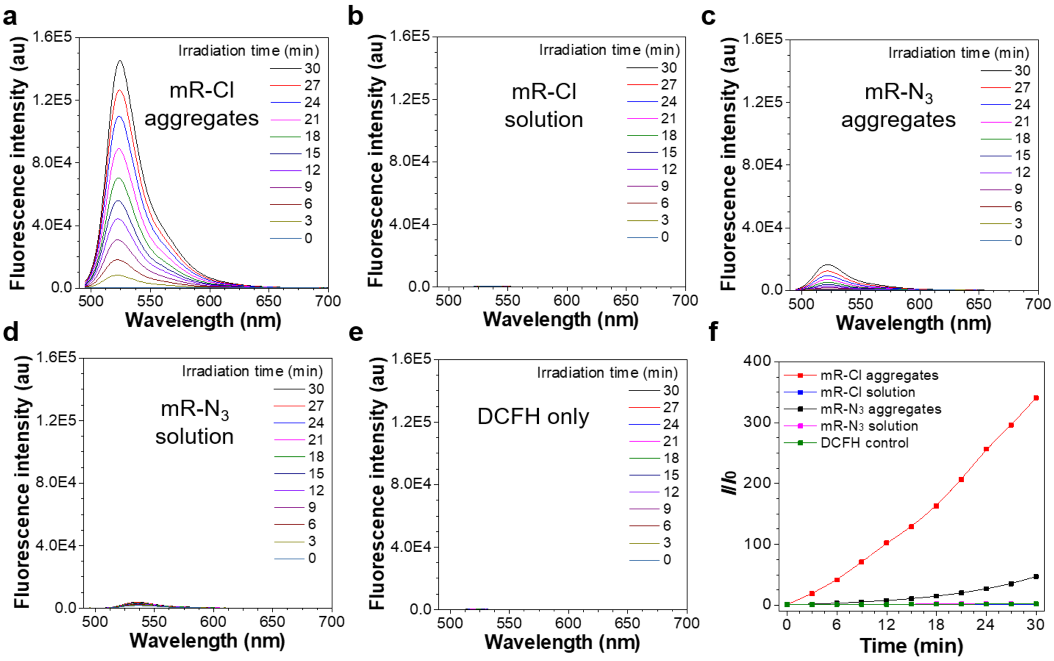

Figure S36. Chemical trapping of total ROS generation. Photoactivation of DCFH mixed with (a) mR-Cl aggregates, (b) mR-Cl solution, (c) mR-N$_3$ aggregates, (d) mR-N$_3$ solution and (e) DCFH control under white light irradiation, respectively. (f) Plotting of the

fluorescence intensity of the DCFH indicator versus the irradiation time in different groups.

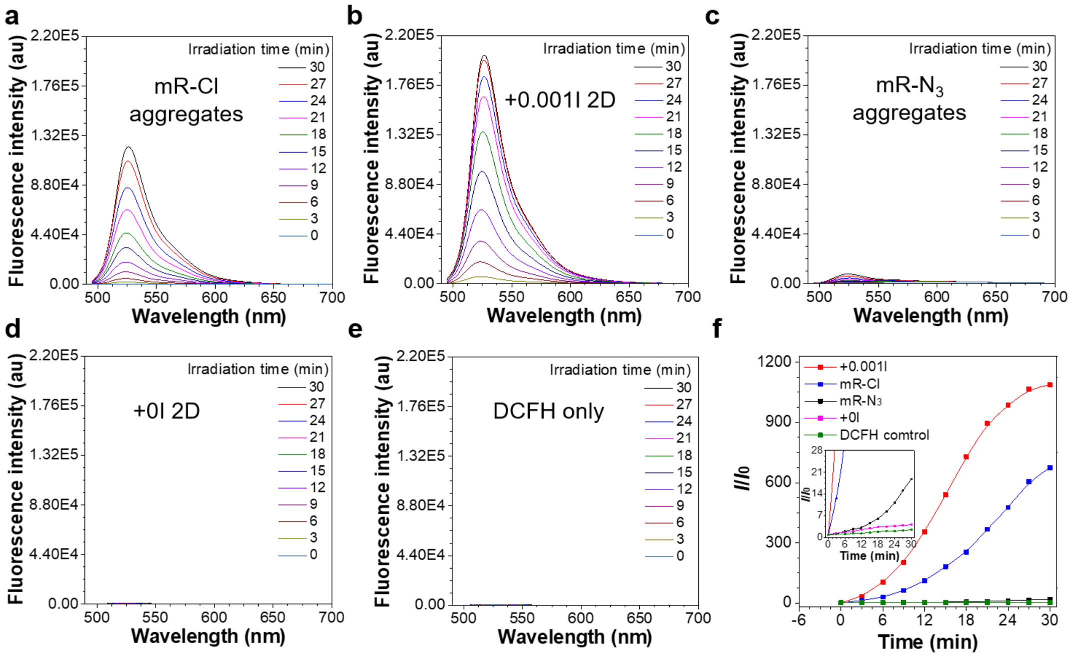

Figure S37. Chemical trapping of total ROS generation. Photoactivation of DCFH with (a) mR-Cl aggregates, (b) 2-D AIEgen-conjugated DNA assembly, (c) mR-N_3 aggregates, (d) 2-D AIE photosensitizer-DNA conjugates without Initiator and (e) DCFH control under white light irradiation, respectively. (f) Plotting of the fluorescence intensity of the DCFH indicator versus the irradiation time in different groups.

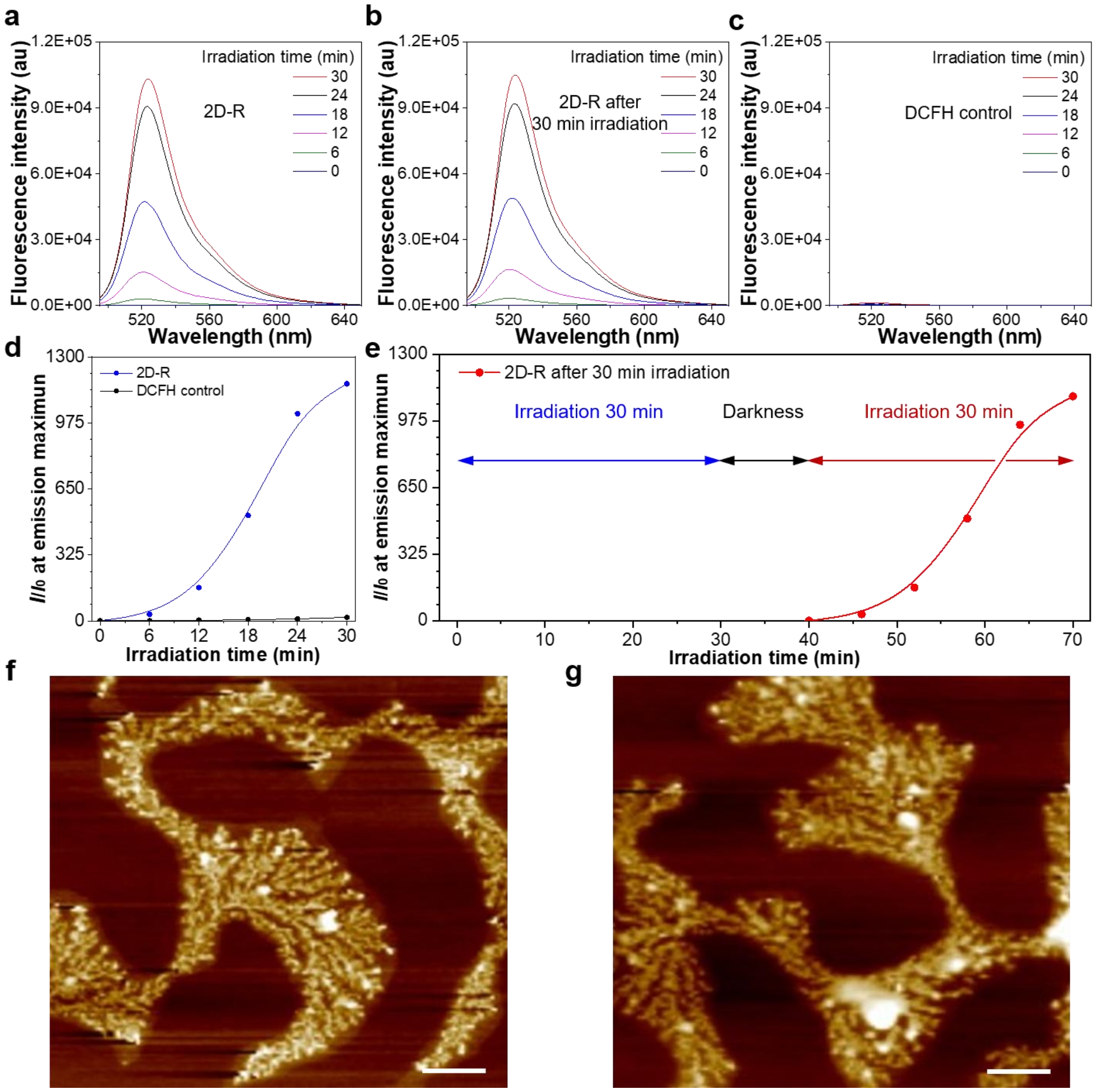

Figure S38. Photoactivation of DCFH with (a) 2-D assemblies via Initiator + $mH_{3\text{-}6}$-R after one-round 30 min white light irradiation, (b) 2-D assemblies via Initiator + $mH_{3\text{-}6}$-R after two-round 30 min white light irradiation, (c) DCFH control in the second round 30 min white light irradiation, respectively. (d) Activation rates of DCFH coincubation with Initiator + $mH_{3\text{-}6}$-R monitoring at 524 nm emission for one-cycle PDT. (e) Activation rates of DCFH coincubation with Initiator + $mH_{3\text{-}6}$-R monitoring at 524 nm emission after two-cycle PDT. [mH-R] = 0.5 $\times 10^{-5}$ M, [DCFH] = 1 $\times 10^{-5}$ M. AFM imaging of the 2-D assembly via $mH_{3\text{-}6}$-R conjugates before (f) and after (g) 30 min white light irradiation. The 0.001-fold Initiator was used as the trigger of HCR reaction. The power of white light was 2 mW•cm^{-2}. All the scale bars were 500 nm.

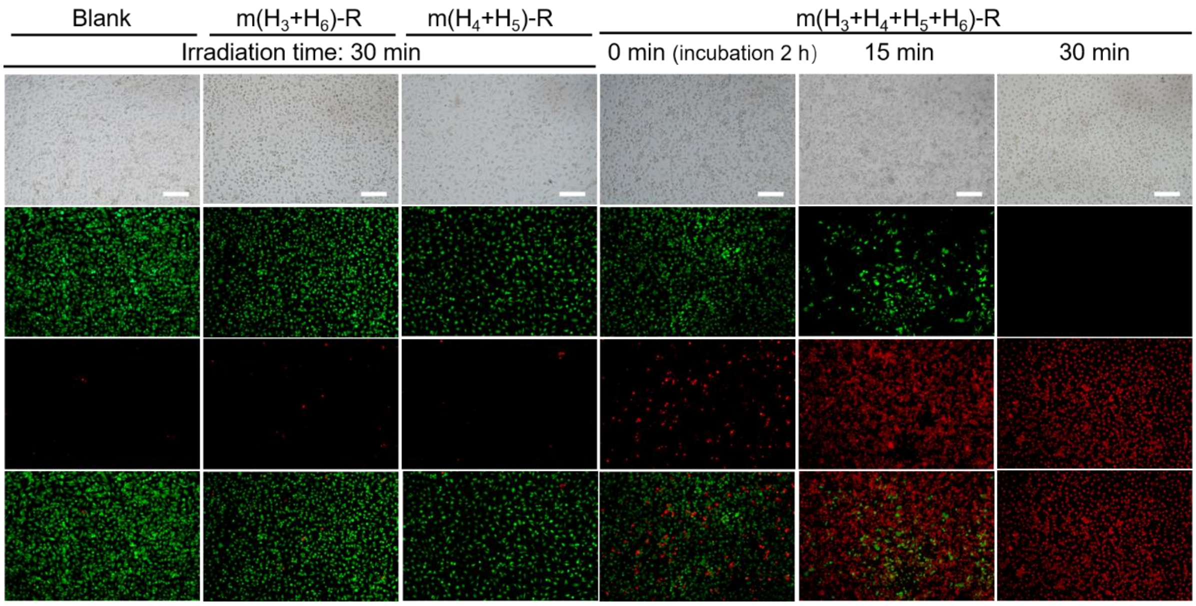

Figure S39. Live/dead HeLa cell staining by Calcein AM/ PI along with the white light irradiation time. All the scale bars were 200 μm.

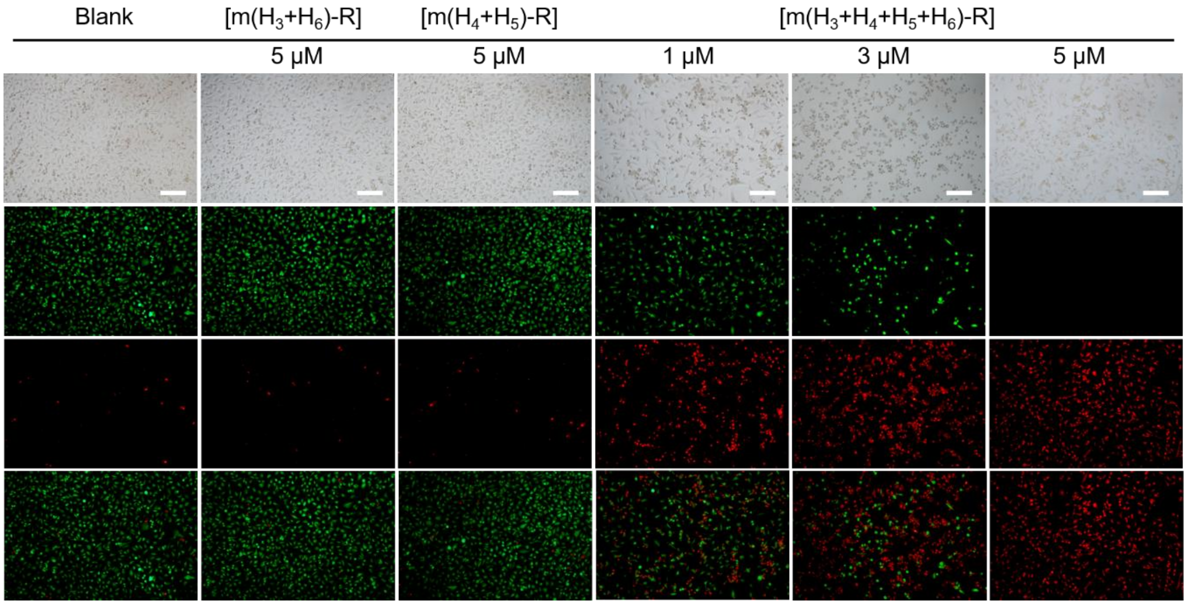

Figure S40. Live/dead HeLa cell staining by Calcein AM/ PI along with the concentration of AIE photosensitizer-DNA conjugates under white light irradiation. All the scale bars were 200 μm.

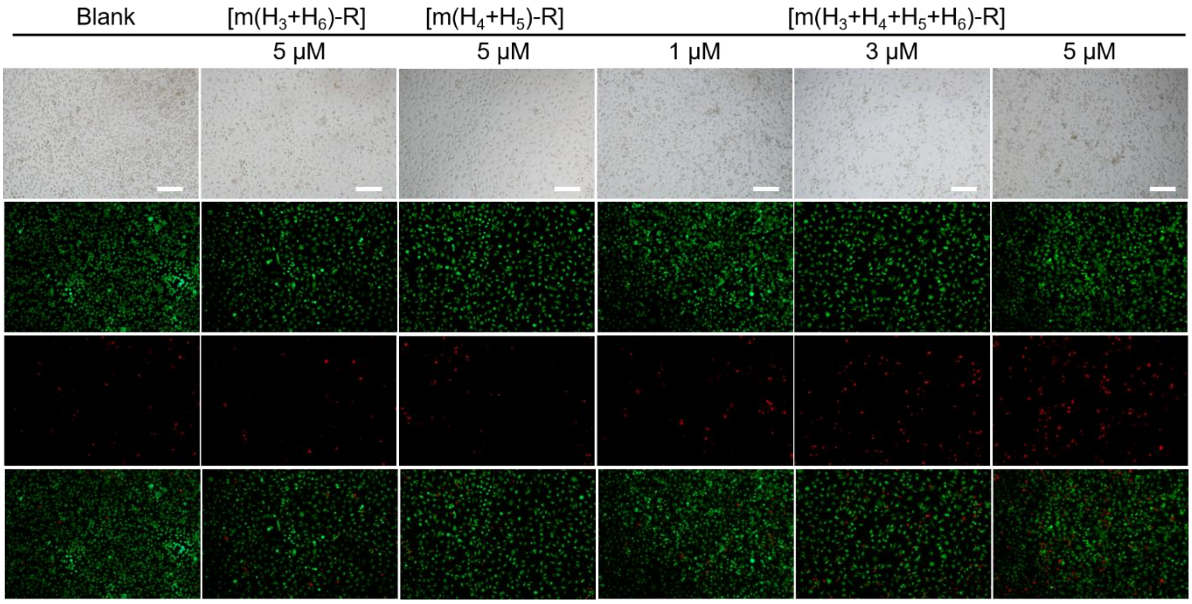

Figure S41. Live/dead HeLa cell staining by Calcein AM/ PI along the concentration of AIE photosensitizer-DNA conjugates with darkness. All the scale bars were 200 μm.

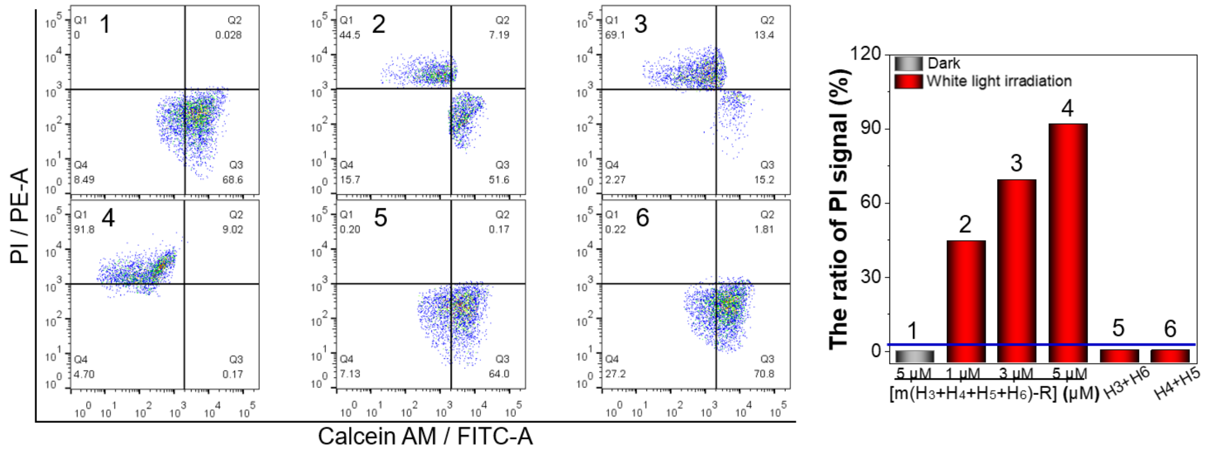

Figure S42. Flow cytometric tests of HeLa cells via detecting the stained Calcein AM-FITC and propidium iodide (PI) after treatment with 2-D AIE photosensitizer-DNA conjugates and 30 min's white light irradiation. The statistic ratio of dead cells number indicated by the PI signal was presented as histogram.

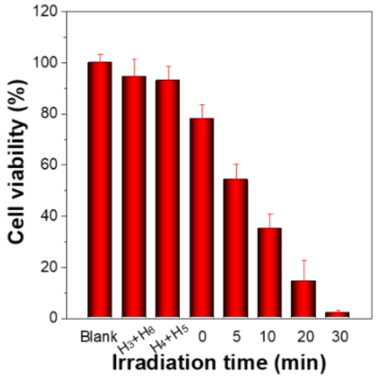

Figure S43. The corresponding HeLa cell viability test of 2-D AIE photosensitizer-DNA conjugates assembly after treatment by different white light irradiation times.

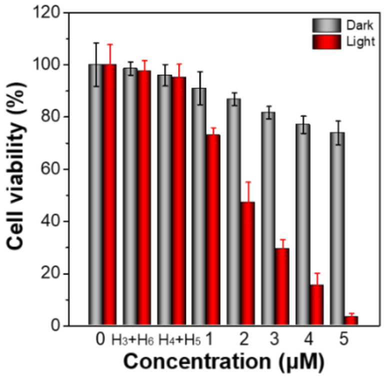

Figure S44. The corresponding HeLa cell viability test after treatment by different concentrations of 2-D AIE photosensitizer-DNA conjugate assembly under white light irradiation or darkness, respectively.

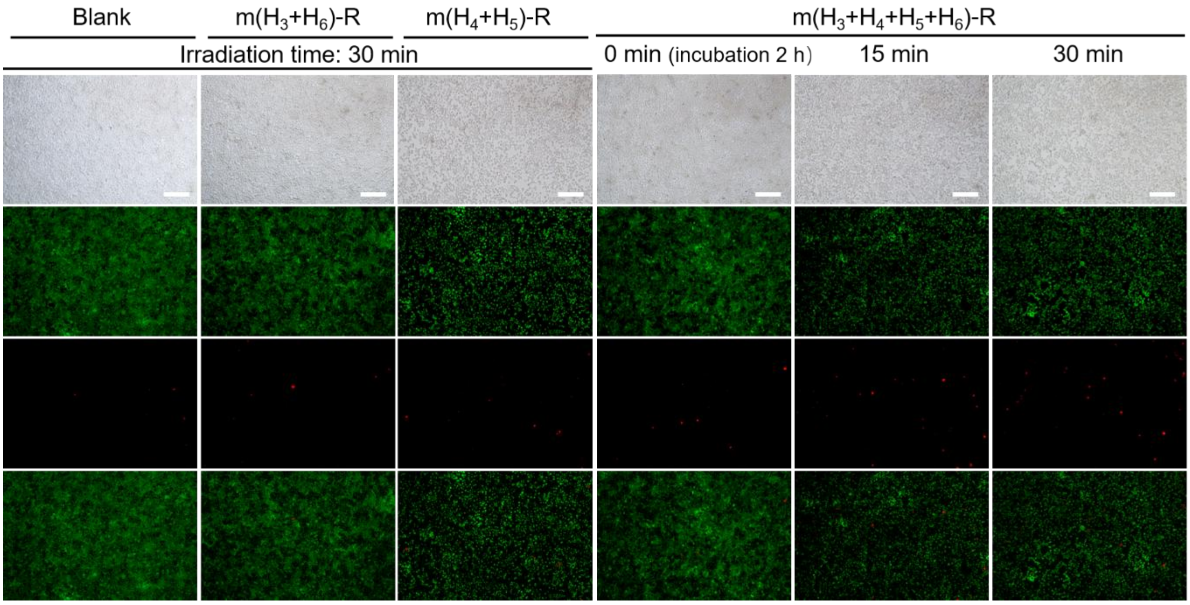

Figure S45. Live/dead HEK-293 cell staining by Calcein AM/ PI along with the white light irradiation time. All the scale bars were 200 μm.

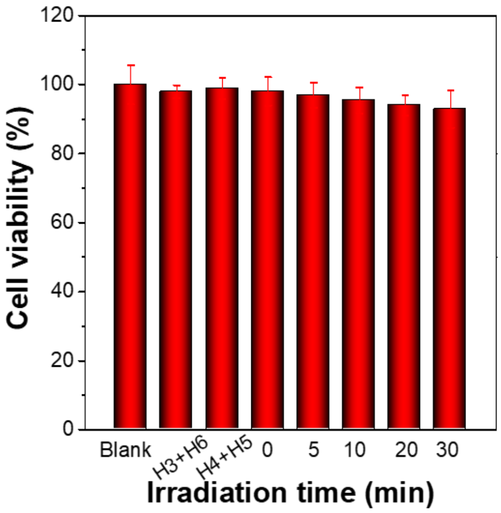

Figure S46. The corresponding HEK-293 cell viability test of 2-D AIE photosensitizer-DNA conjugate assembly after treatment by different white light irradiation times.

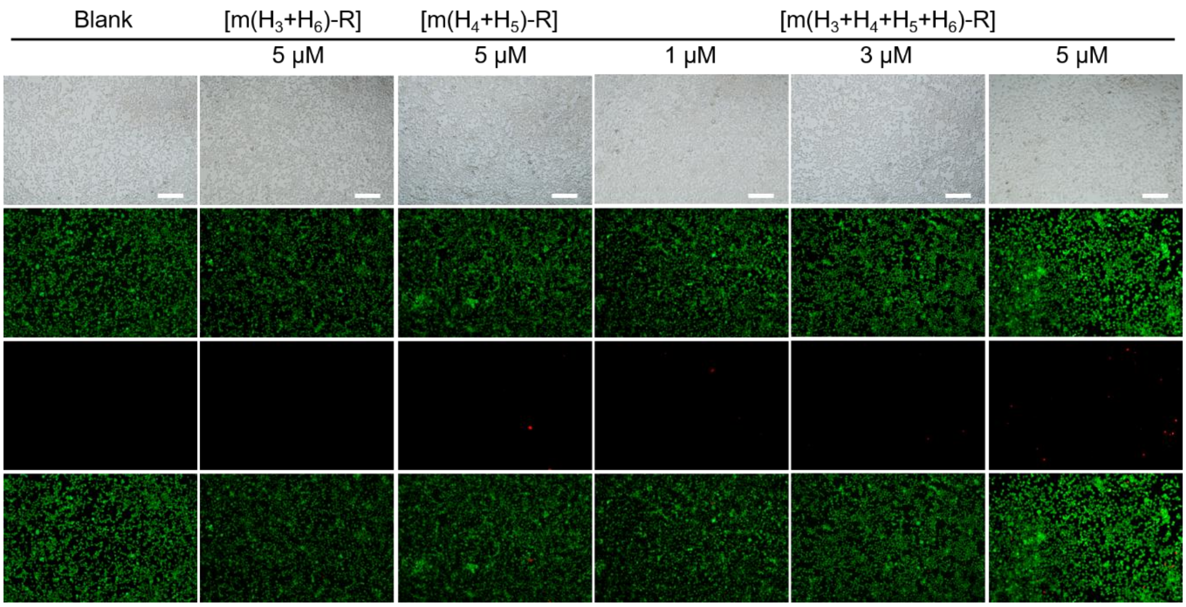

Figure S47. Live/dead HEK-293 cell staining by Calcein AM/ PI along the concentration of AIE photosensitizer-DNA conjugates under white light irradiation. All the scale bars were 200 μm.

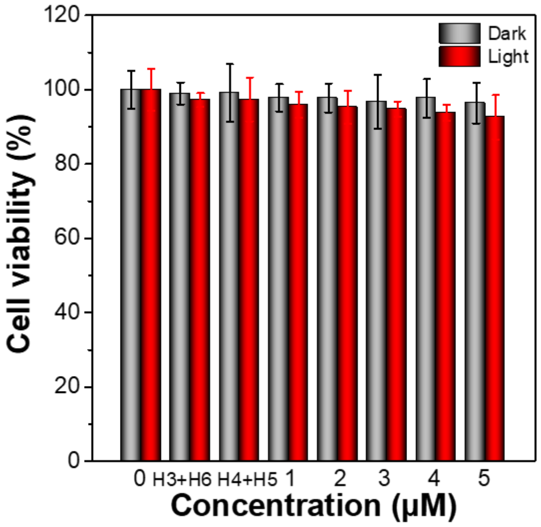

Figure S48. The corresponding HEK-293 cell viability test after treatment by different concentrations of 2-D AIE photosensitizer-DNA conjugates assembly under white light irradiation or darkness, respectively.

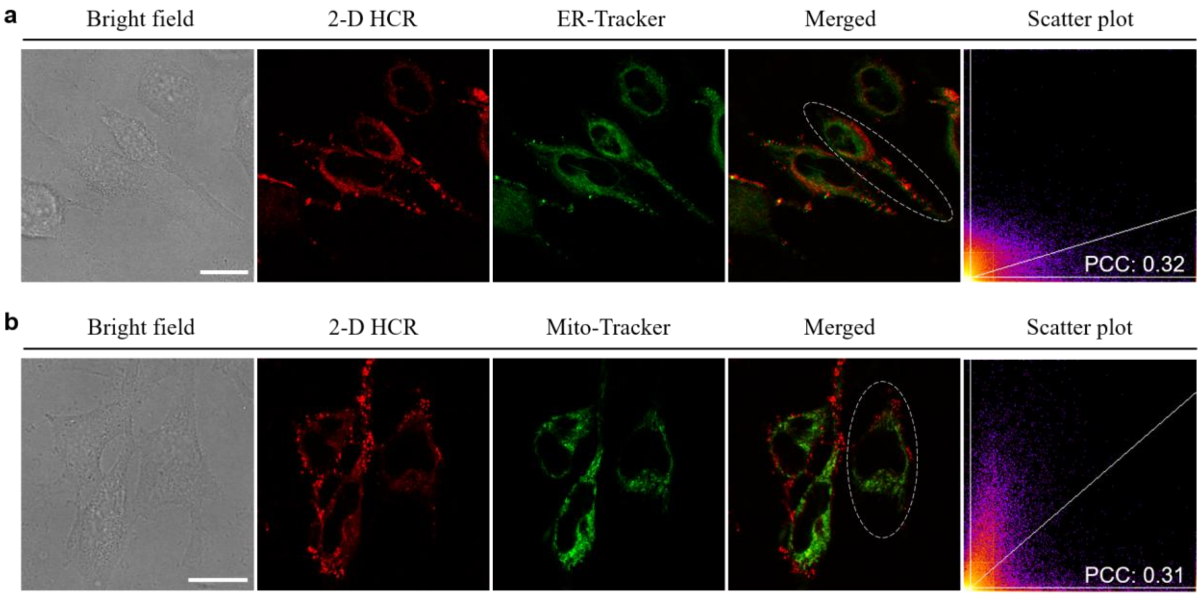

Figure S49. Confocal laser scanning imaging of 2D AIEgen-DNA assembly in HeLa cells to determine their co-localization with the endoplasmic reticulum or mitochondria. (a) Colocalization staining with endoplasmic reticulum-tracker red. The red channel for mR-N_3: ex 455 nm, em 530-650 nm; Green channel for ER-Tracker red: ex 561 nm, em 580-700 nm. (b) Colocalization staining with mitochondria-tracker deep red. The Red channel for mR-N_3: ex 455 nm, em 530-650 nm; Green channel for Mito-Tracker deep red: ex 633 nm, em 650-750 nm. The scatter plots were Pearson's correlation coefficient (PCC). Scale bar: 20 μm.

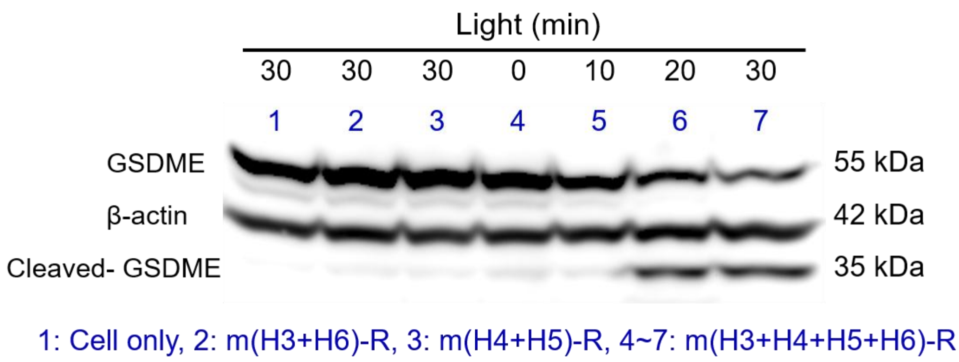

Figure S50. GSDME and cleaved GSDME expression levels within HeLa cells as detected by western blotting. Actin was used as a control for protein loading.

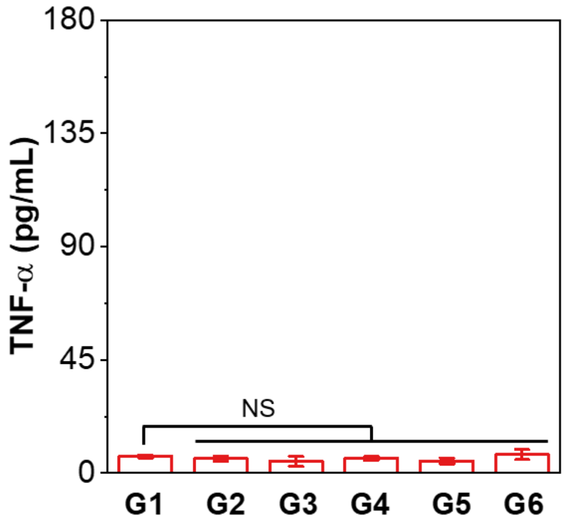

Figure S51. ELISA quantitation of TNF-α release from HeLa cells after different treatments: G1: HeLa cells only + irradiation 30 min, G2: mH_3-R + mH_6-R + irradiation 30 min, G3: mH_4-R + mH_5-R + irradiation 30 min, G4: $mH_{3\text{-}6}$-R - darkness, G5: $mH_{3\text{-}6}$-R + irradiation 10 min, G6: $mH_{3\text{-}6}$-R + irradiation 30 min. The power of white light power was 2 mW•cm^{-2}. NS: no significance.

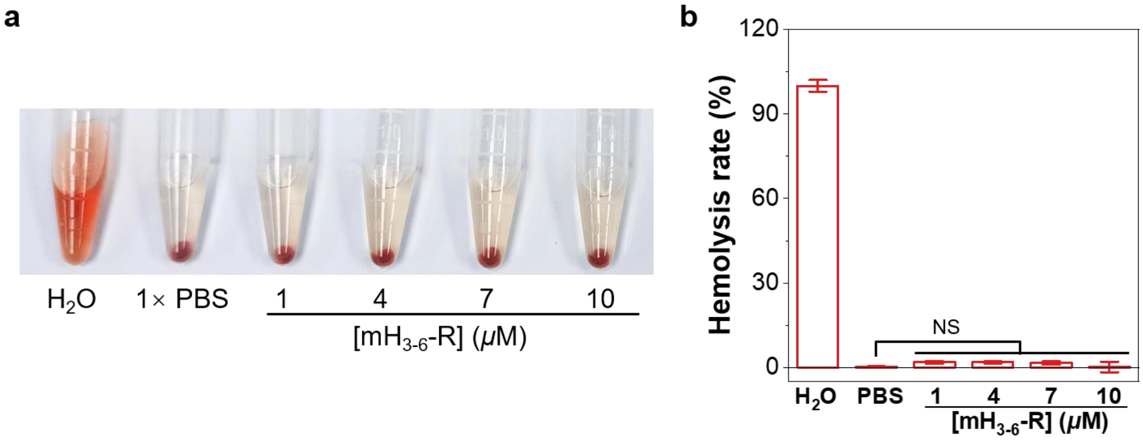

Figure S52. Hemolytic activity of $mH_{3\text{-}6}$-R conjugates. (a) Photographs of red blood cells after treatment with different concentrations of $mH_{3\text{-}6}$-R. (b) Quantitative analysis of the hemolysis rate of red blood cells after treatment with different concentrations of different $mH_{3\text{-}6}$-R. The hemolysis rate of red blood cells treated with PBS was set as 0%, and that treated with H_2O was set as 100%. NS: no significance.

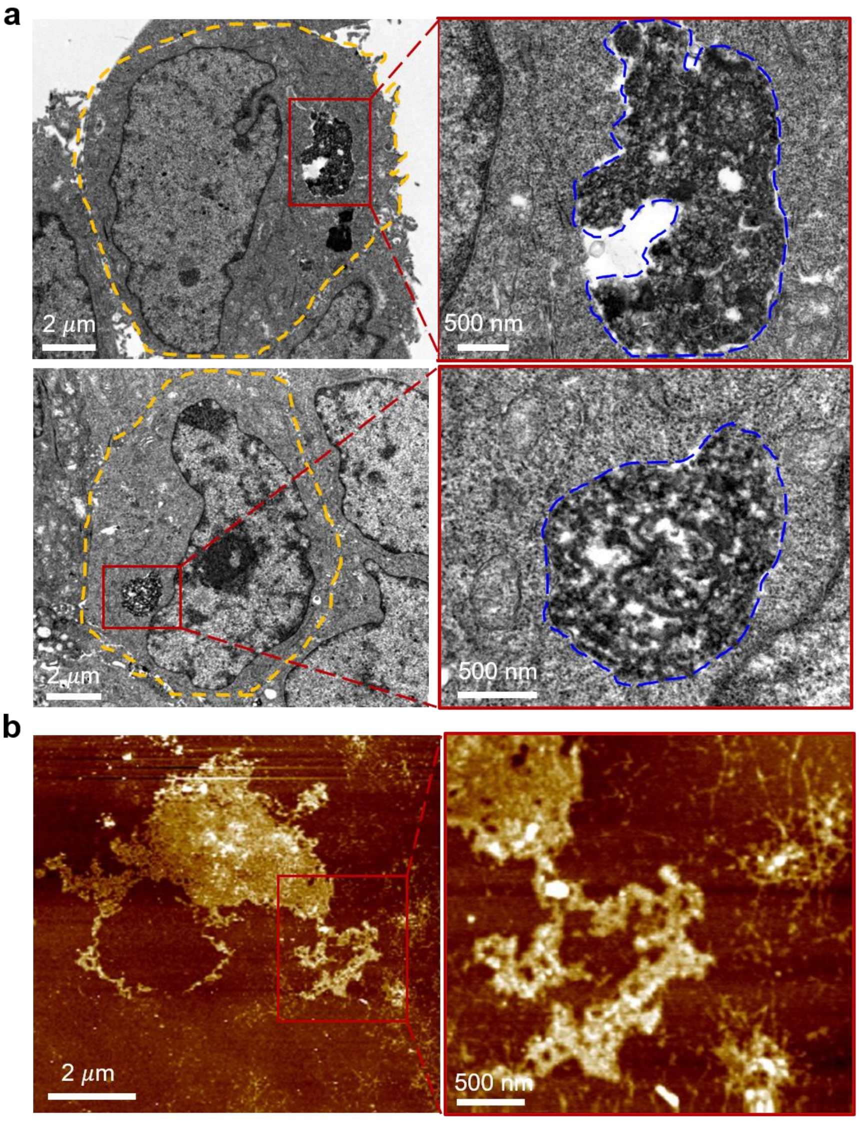

Figure S53. (a) TEM imaging of the 2-D assembly via tumor slices collected from tumor-bearing mice after treatment by mH_{3-6}-R conjugates. The cells were profiled by the yellow-color dash line, and the assembly formed zones were marked by the red-color solid line box with details showing in the magnified pictures. (b) AFM imaging of the 2-D assembly via tumor tissue lysis supernatant after treatment by mH_{3-6}-R conjugates.

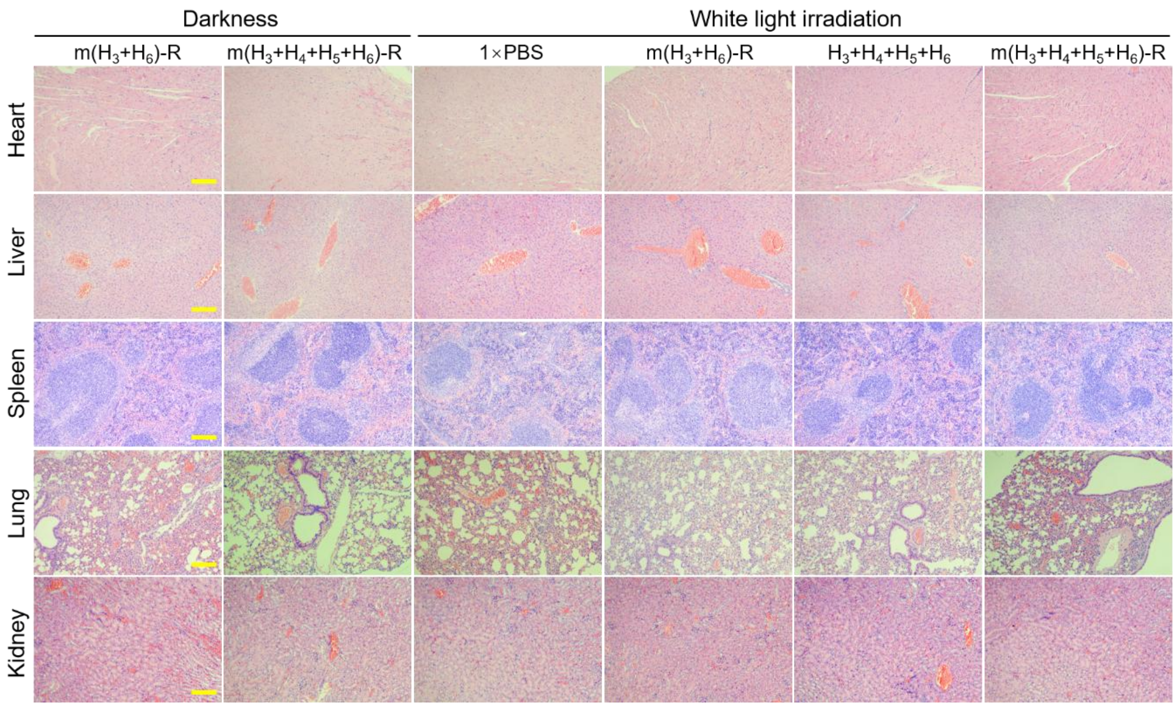

Figure S54. H&E-staining of heart, liver, spleen, lung, and kidney retrieved from tumor bearing mice after various treatment with white light irradiation or darkness, respectively. All the scale bars were 200 μm.

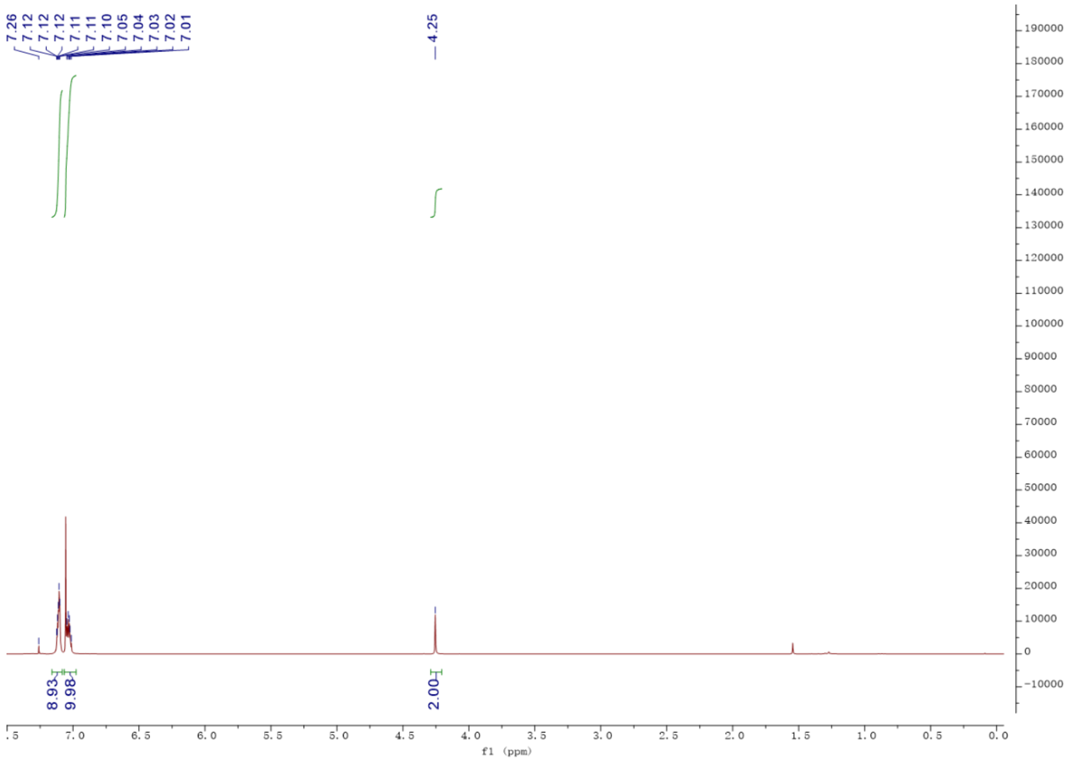

^{1}H NMR spectrum of **mB-N$_3$** in CDCl$_3$.

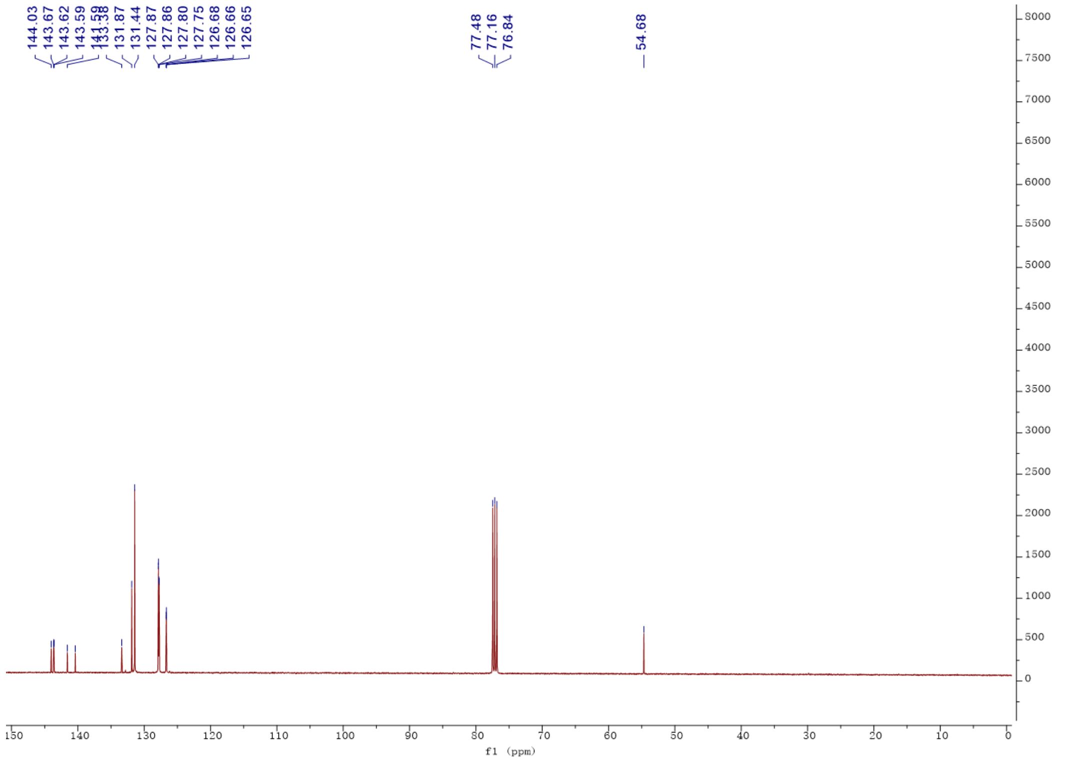

^{13}C NMR spectrum of **mB-N$_3$** in $CDCl_3$.

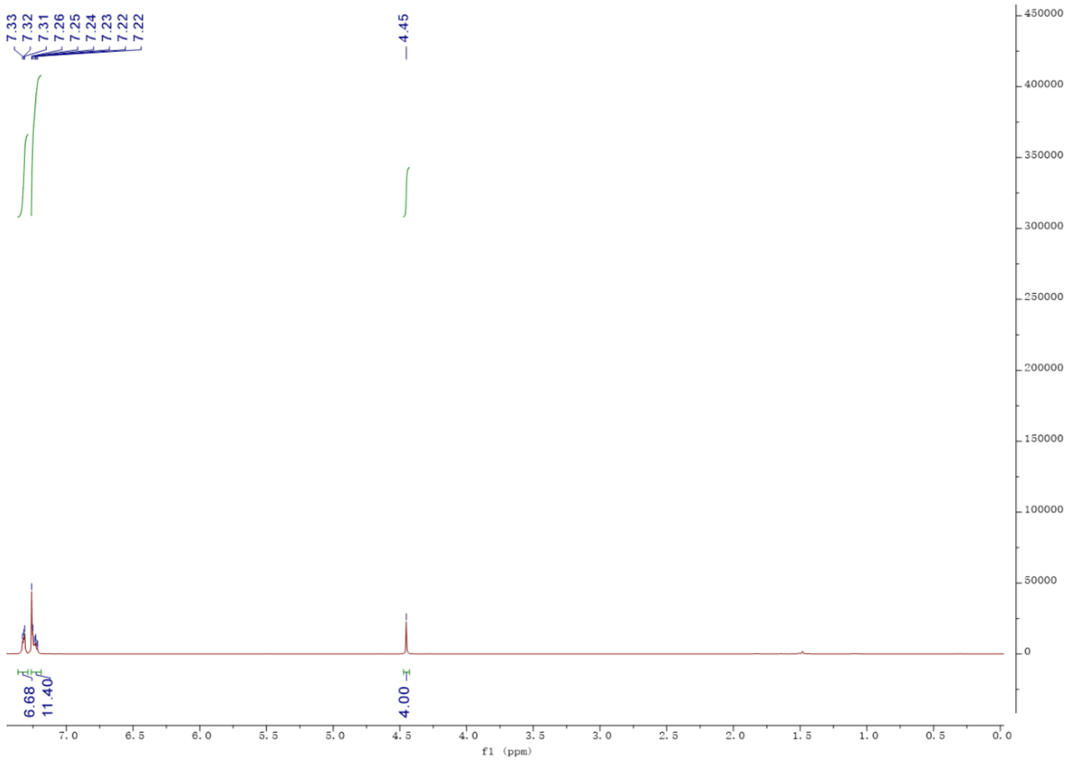

^{1}H NMR spectrum of **dB-N$_3$** in $CDCl_3$.

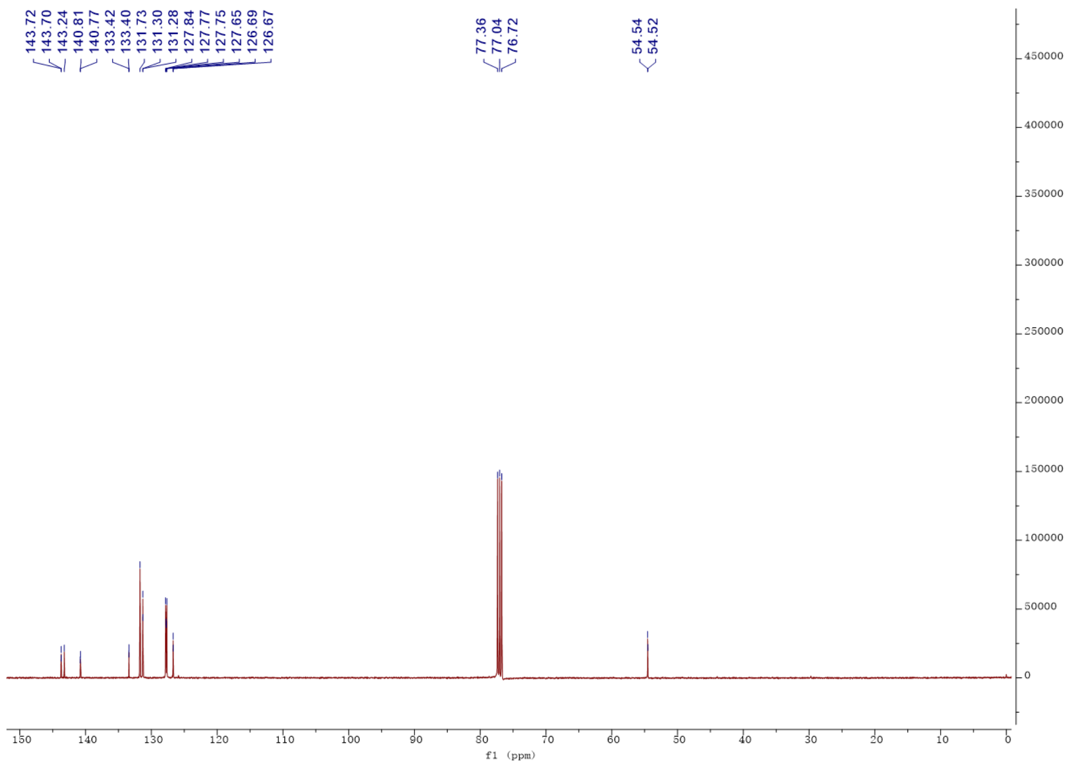

^{13}C NMR spectrum of **dB-N$_3$** in $CDCl_3$.

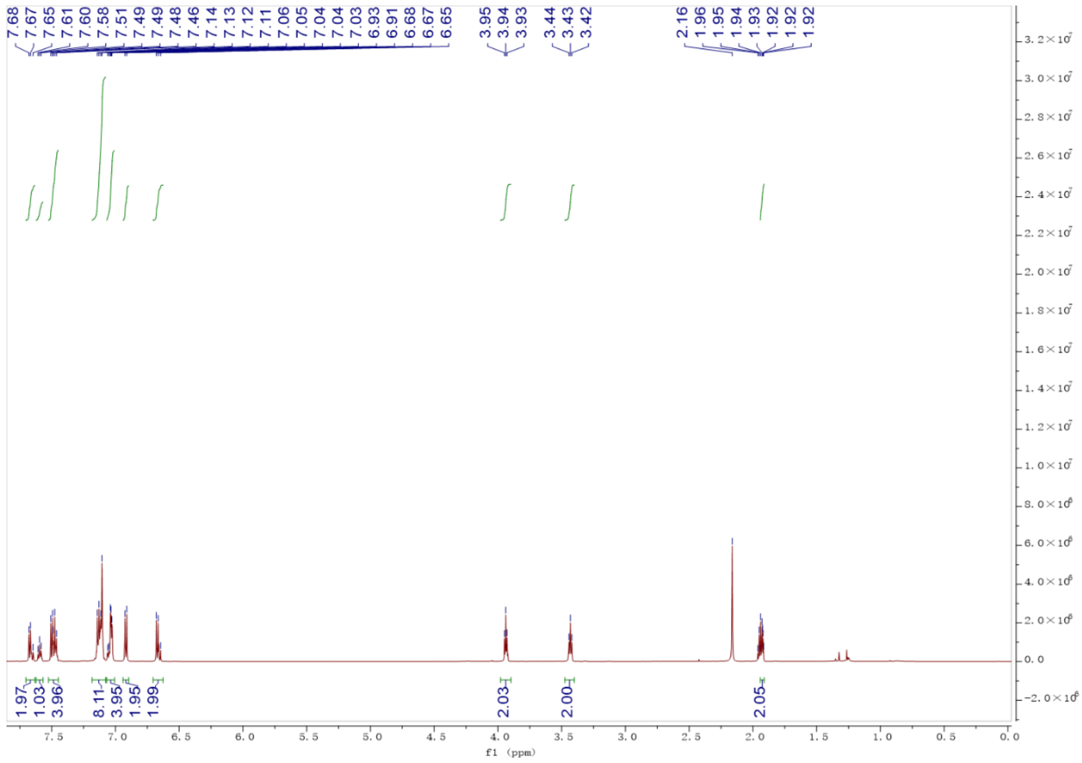

^{1}H NMR spectrum of **mY-N$_3$** in CD_3CN.

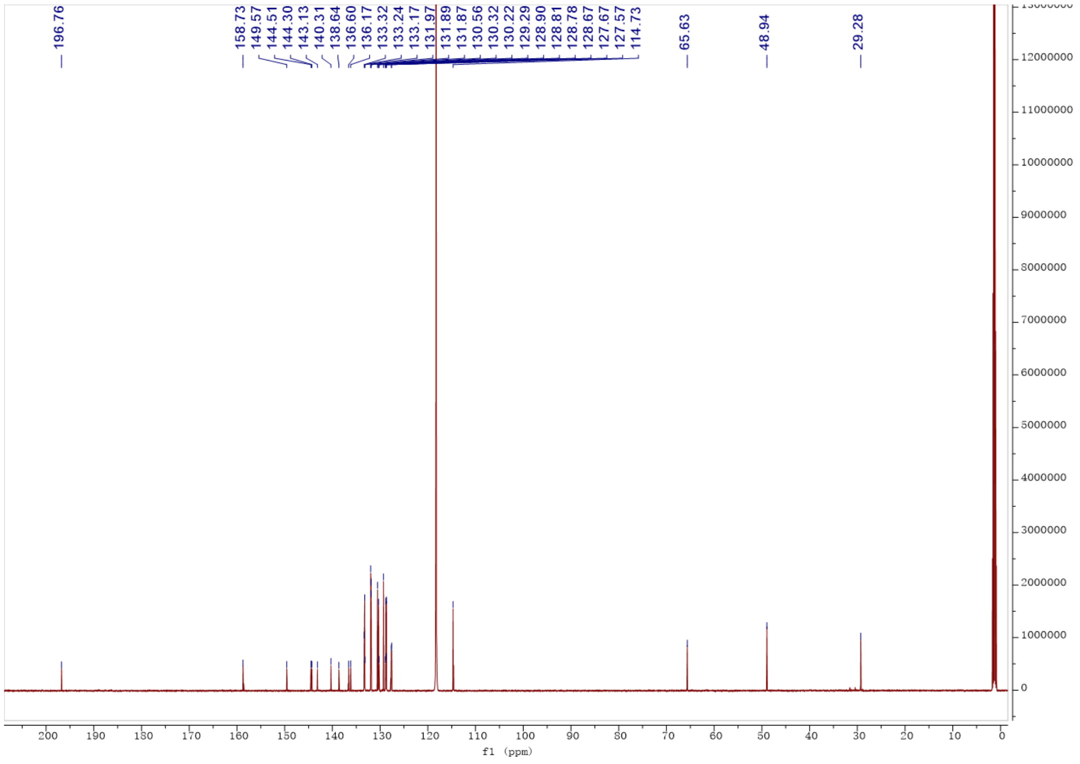

^{13}C NMR spectrum of **mY-N$_3$** in CD_3CN.

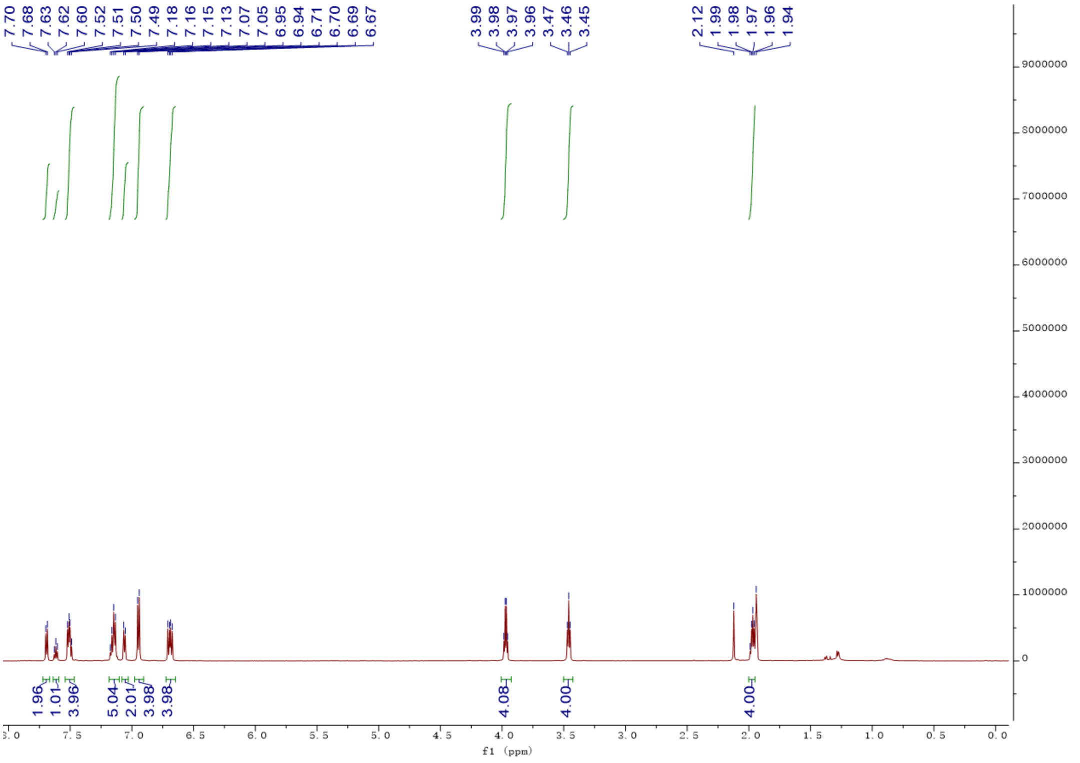

^{1}H NMR spectrum of **dY-N$_3$** in CD_3CN.

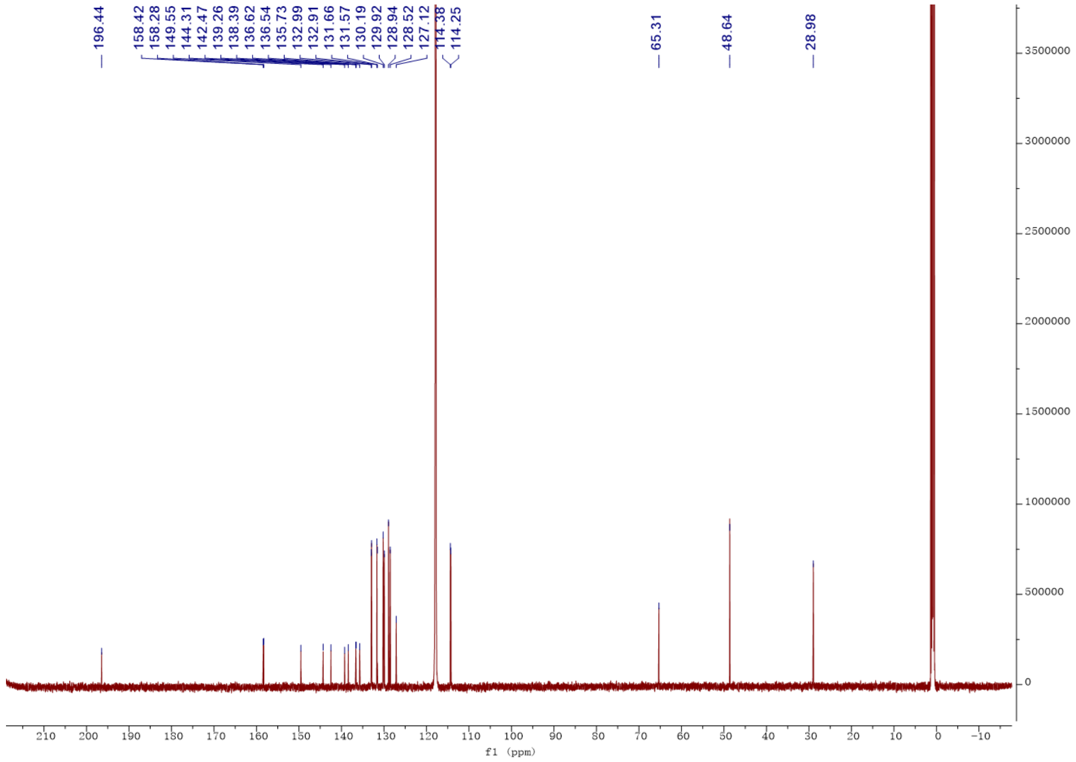

^{13}C NMR spectrum of **mY-N$_3$** in CD_3CN.

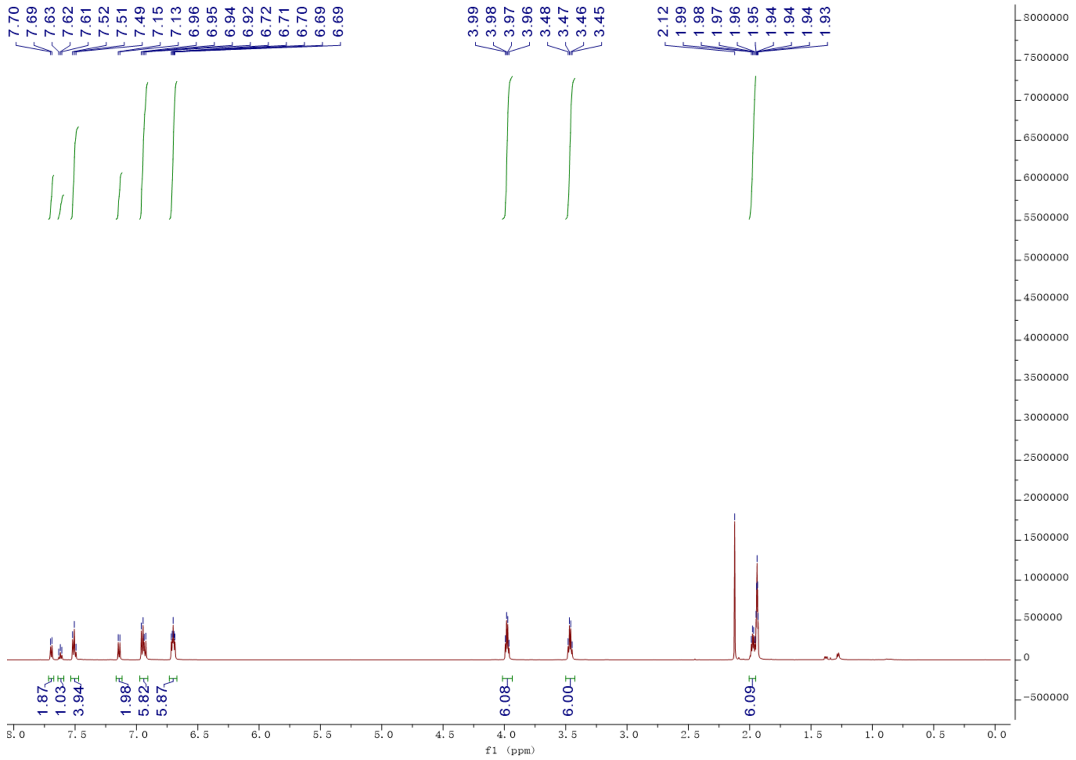

^{1}H NMR spectrum of **tY-N$_3$** in CD_3CN.

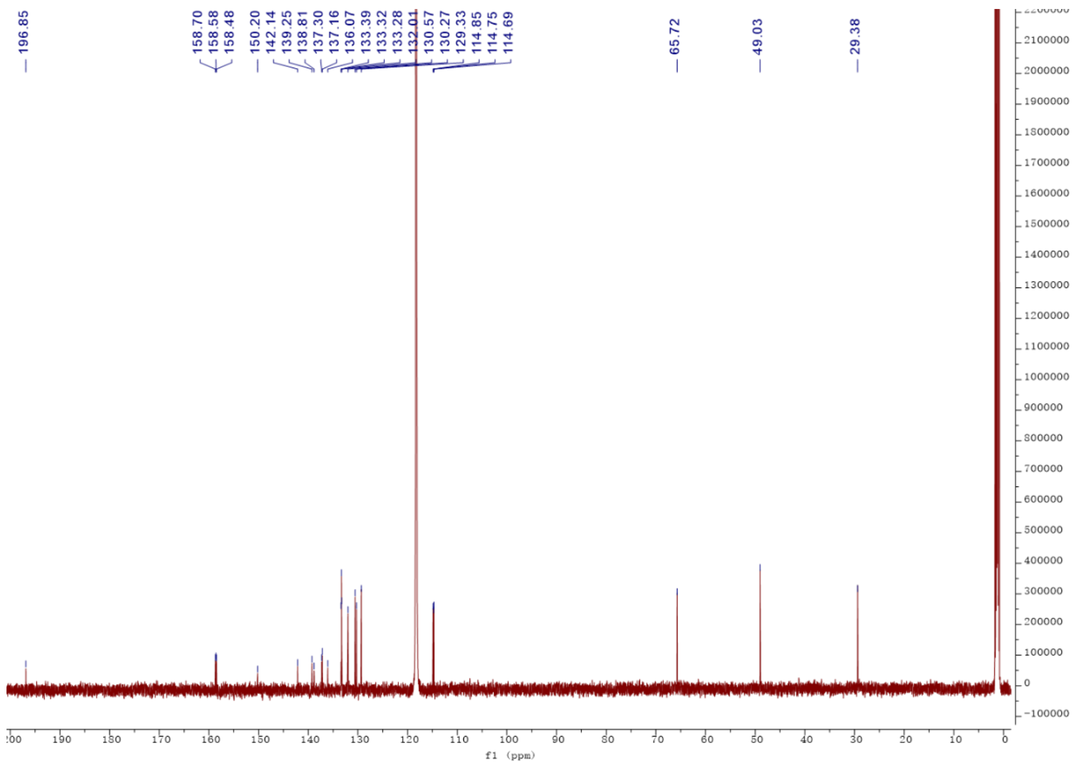

^{13}C NMR spectrum of **tY-N$_3$** in CD_3CN.

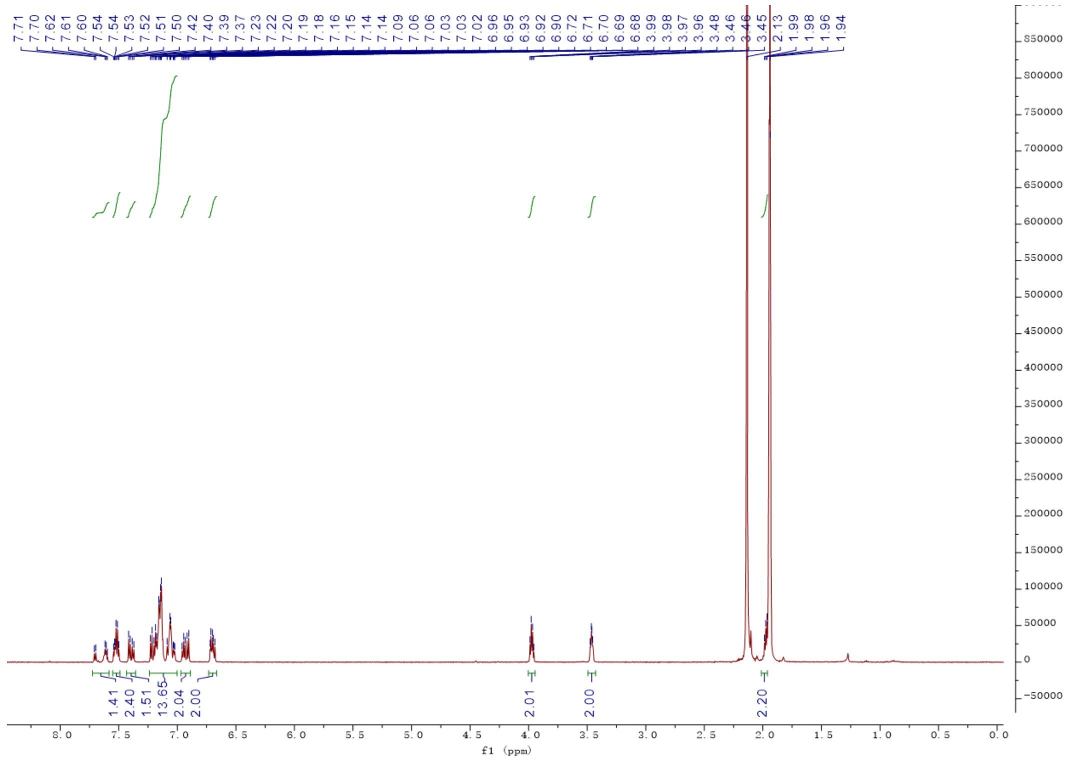

^{1}H NMR spectrum of **mR-N$_3$** in CD_3CN.

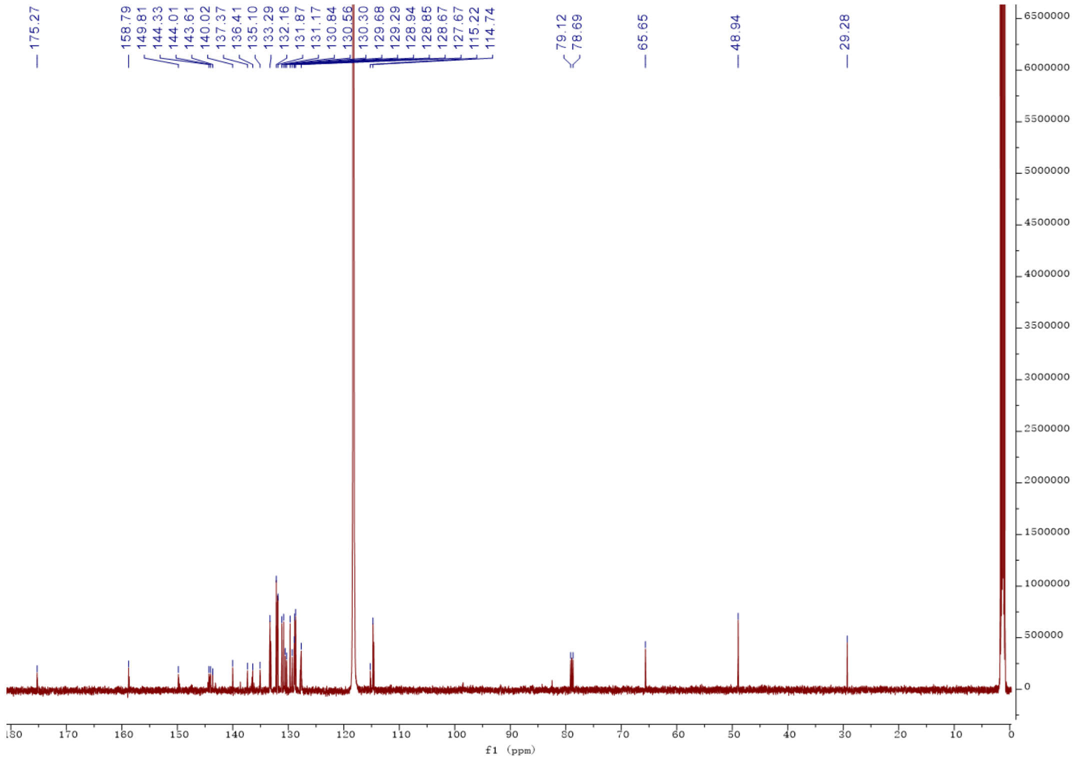

^{13}C NMR spectrum of **mR-N$_3$** in CD_3CN.

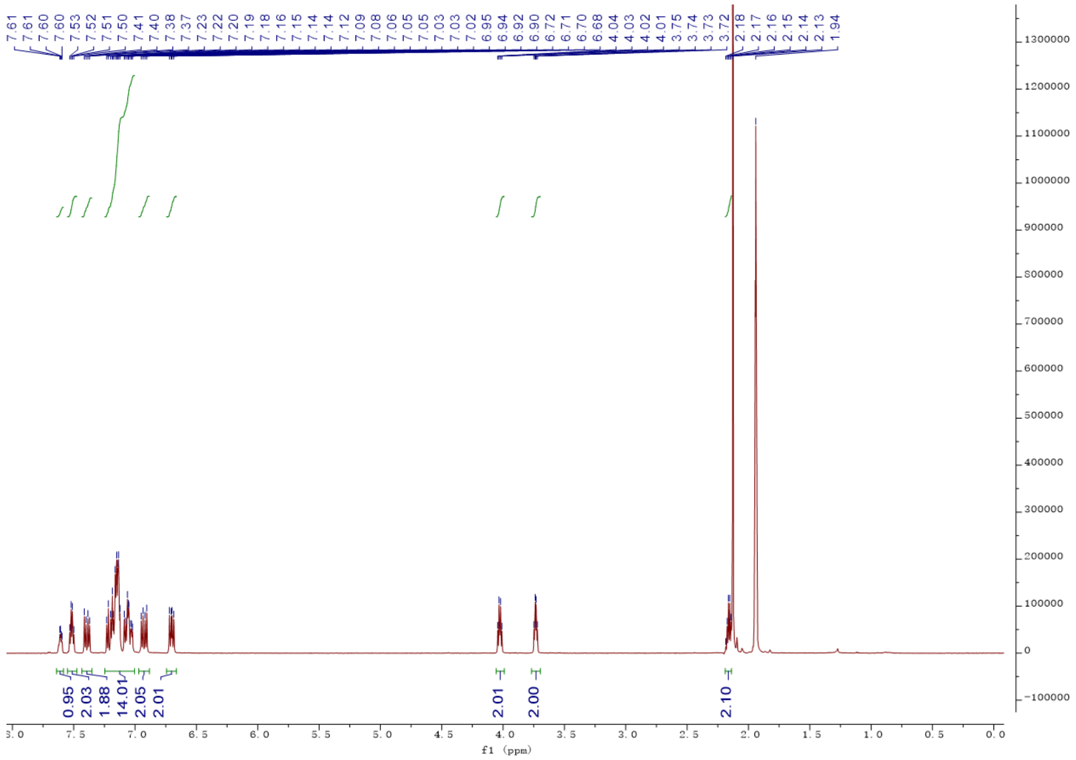

^{1}H NMR spectrum of **mR-Cl** in CD_3CN.

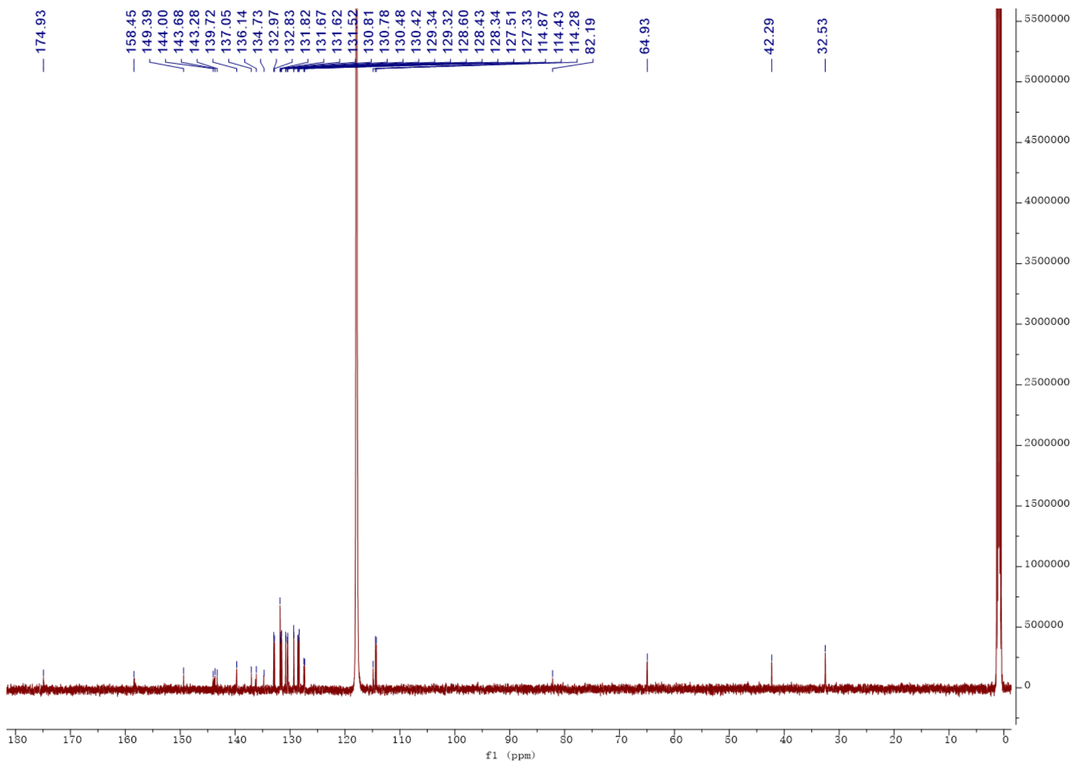

^{13}C NMR spectrum of **mR-Cl** in CD_3CN.

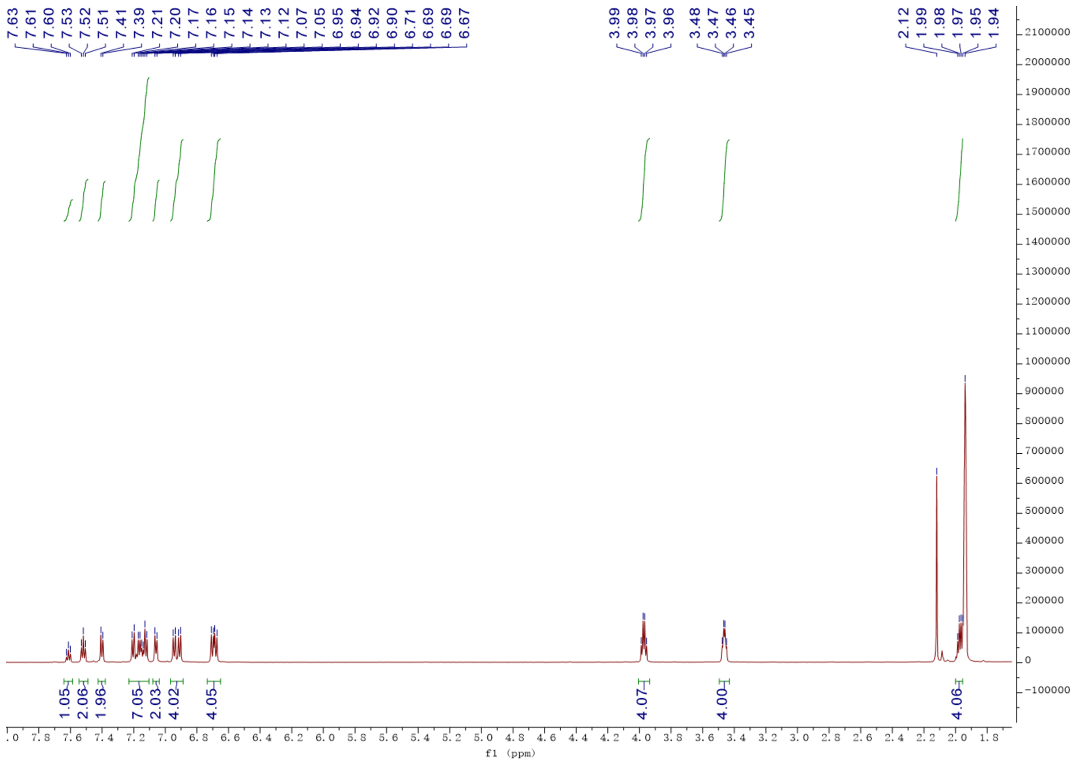

^{1}H NMR spectrum of **dR-N$_3$** in CD_3CN.

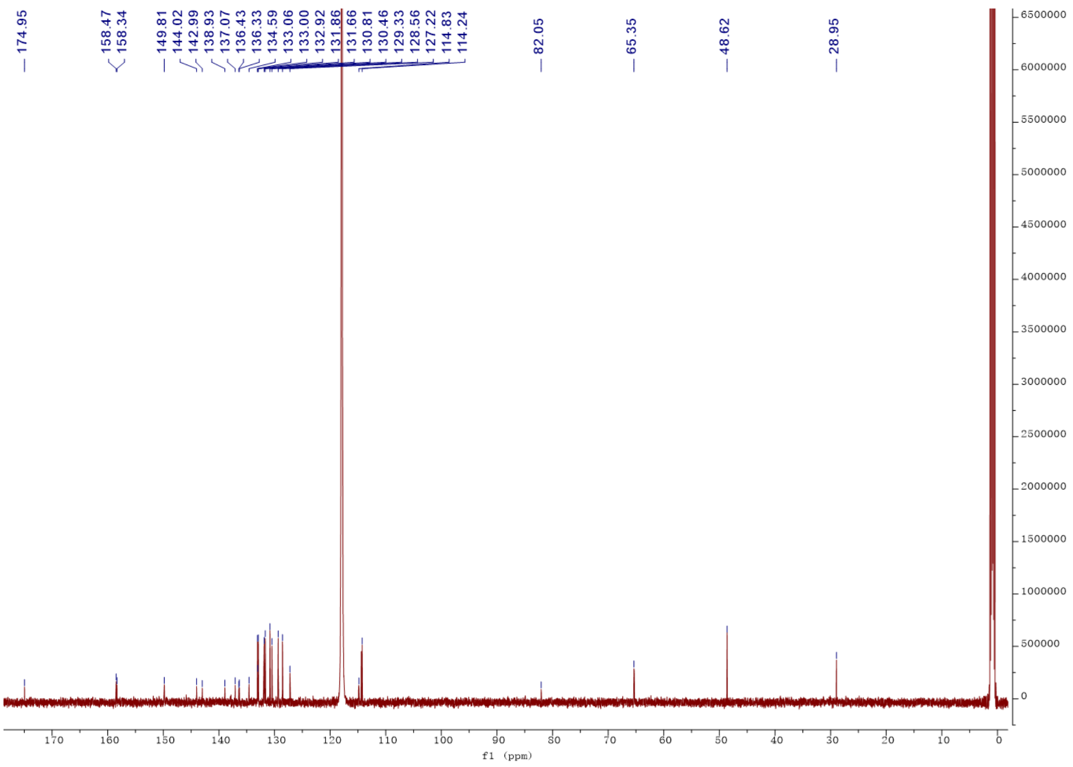

^{13}C NMR spectrum of **dR-N$_3$** in CD_3CN.

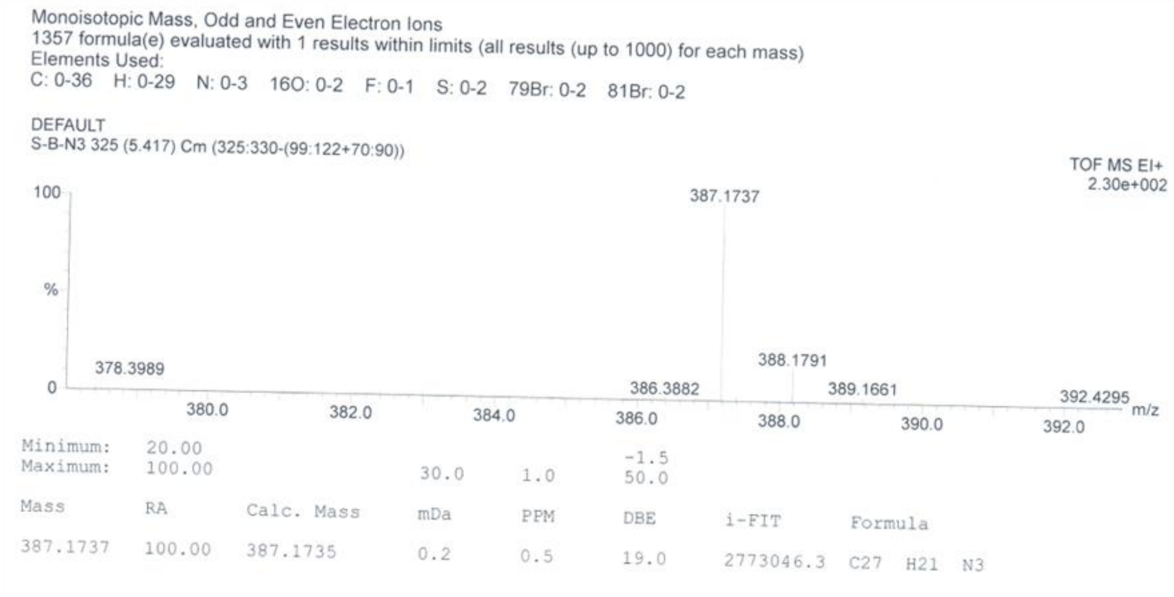

High resolution mass spectrum of **mB-N$_3$.**

Monoisotopic Mass, Odd and Even Electron Ions
812 formula(e) evaluated with 1 results within limits (all results (up to 1000) for each mass)
Elements Used:
C: 0-28 H: 0-22 N: 0-6 28Si: 0-1 79Br: 0-4 81Br: 0-4

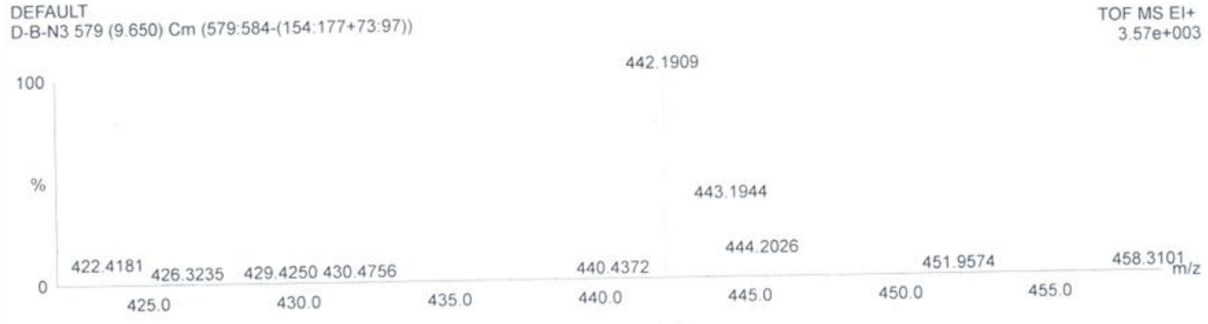

Minimum:	20.00				-1.5		
Maximum:	100.00		30.0	10.0	50.0		
Mass	RA	Calc. Mass	mDa	PPM	DBE	i-FIT	Formula
442.1909	100.00	442.1906	0.3	0.7	21.0	7.8	C28 H22 N6

High resolution mass spectrum of **dB-N$_3$.**

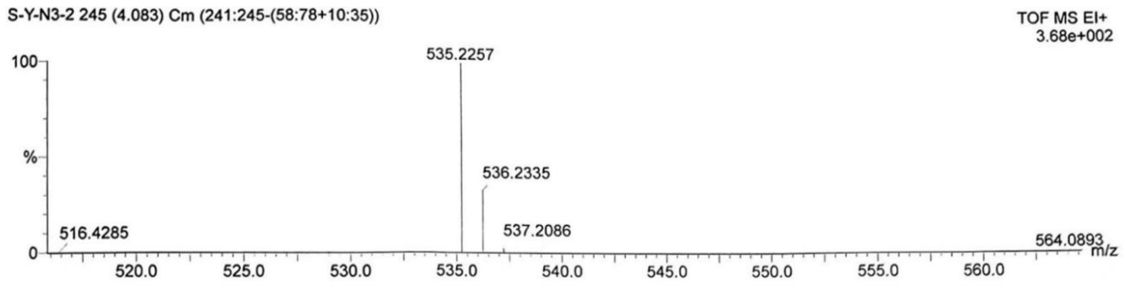

Minimum:	10.00				-1.5		
Maximum:	100.00		30.0	10.0	50.0		
Mass	RA	Calc. Mass	mDa	PPM	DBE	i-FIT	Formula
535.2257	100.00	535.2260	-0.3	-0.6	24.0	60.3	C36 H29 N3 16O2

High resolution mass spectrum of **mY-N$_3$.**

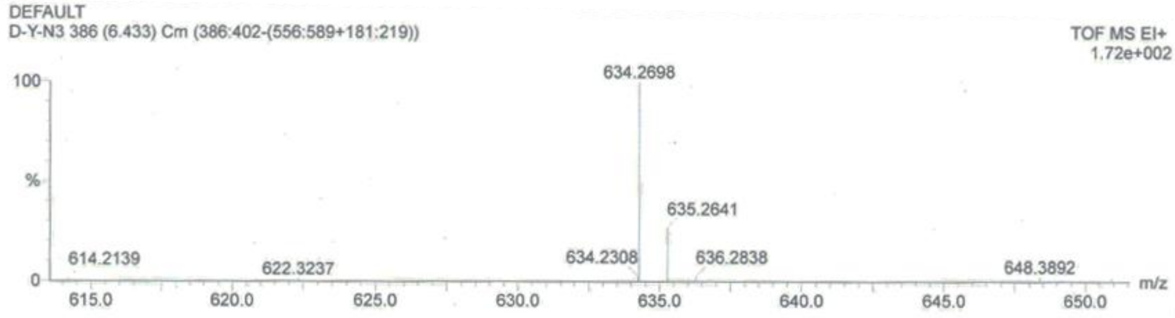

Minimum:	20.00				-1.5		
Maximum:	100.00		30.0	5.0	50.0		
Mass	RA	Calc. Mass	mDa	PPM	DBE	i-FIT	Formula
634.2698	100.00	634.2692	0.6	0.9	26.0	19.3	C39 H34 N6 16O3

High resolution mass spectrum of **dY-N$_3$.**

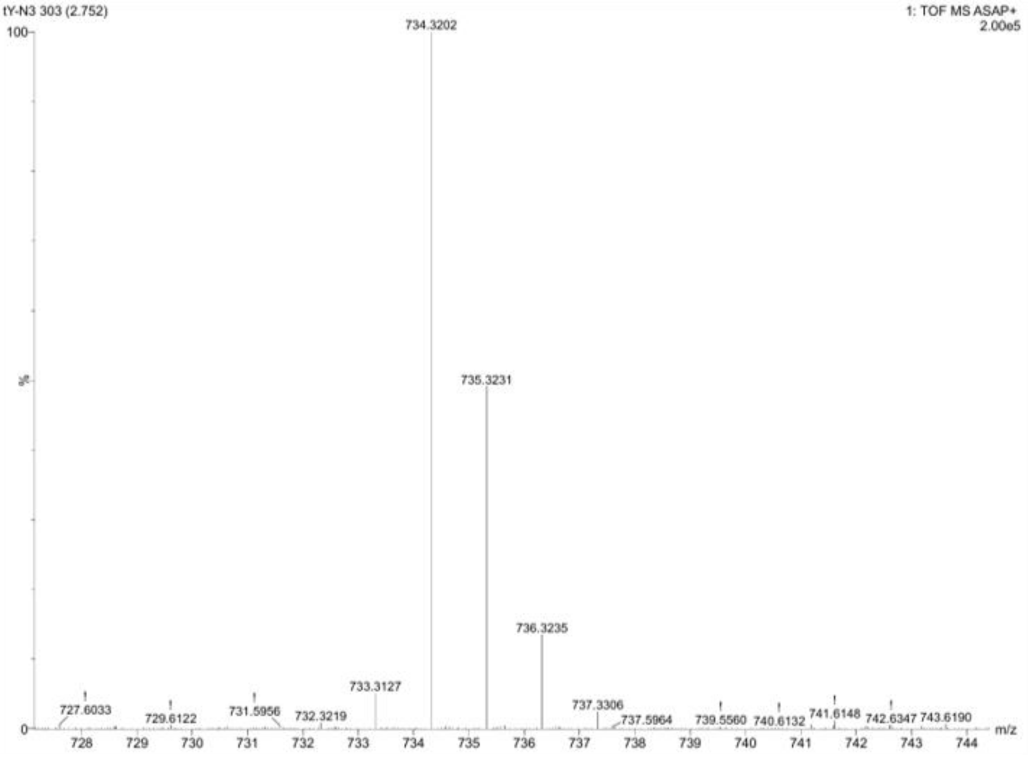

High resolution mass spectrum of **tY-N$_3$.**

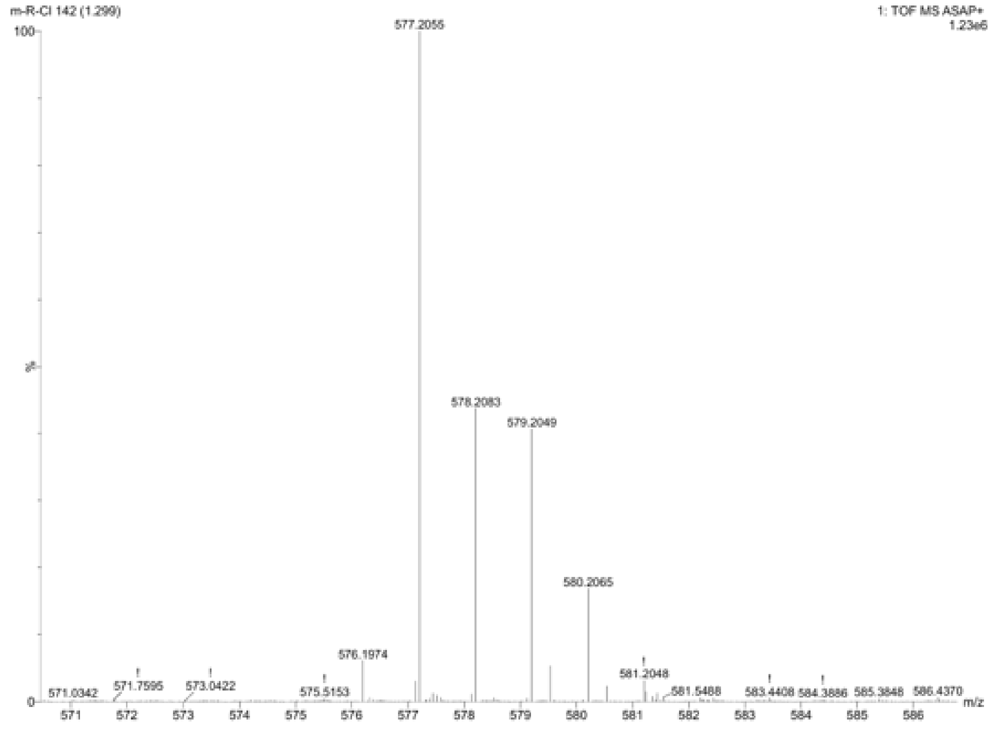

High resolution mass spectrum of **mR-Cl.**

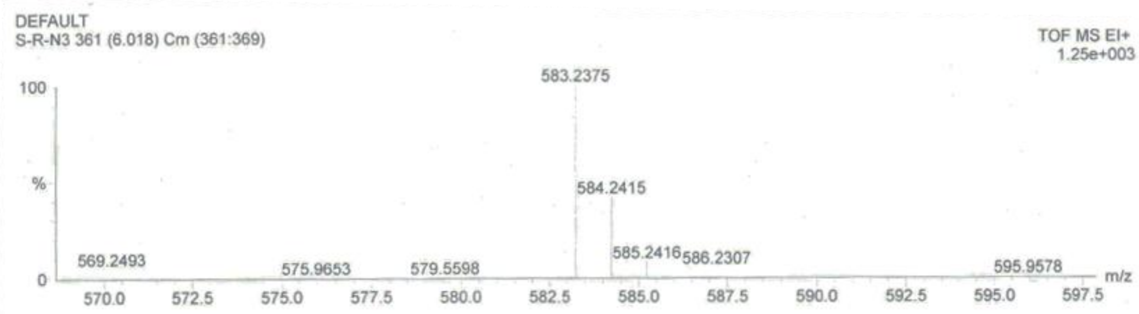

Minimum:	10.00				-1.5		
Maximum:	100.00		30.0	5.0	50.0		
Mass	RA	Calc. Mass	mDa	PPM	DBE	i-FIT	Formula
583.2375	100.00	583.2372	0.3	0.5	28.0	4.2	C39 H29 N5 16O

High resolution mass spectrum of **mR-N$_3$.**

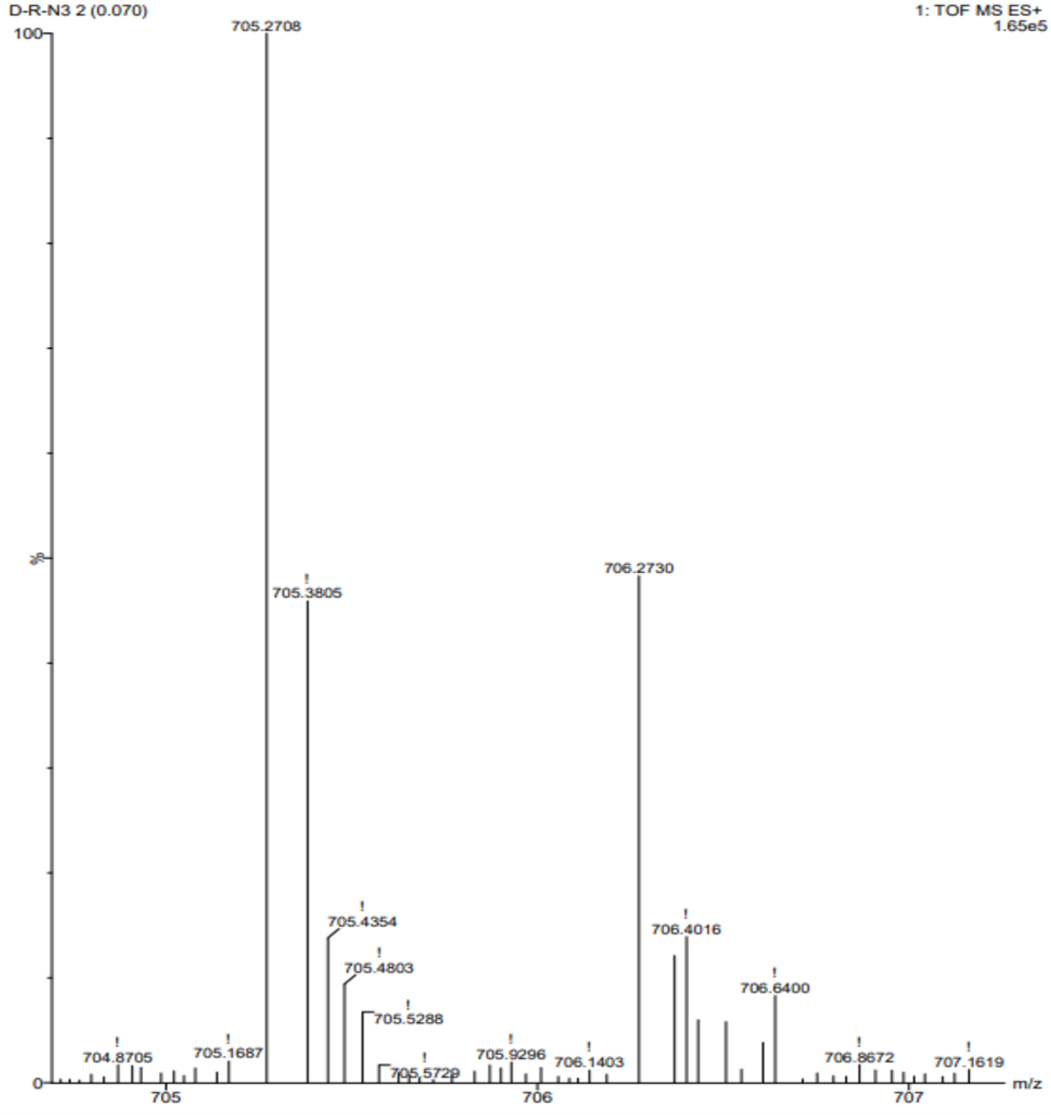

High resolution mass spectrum of **dR-N$_3$.**

References

S1. Dirks RM, Pierce NA. Triggered amplification by hybridization chain reaction. *Proc Natl Acad Sci* 2004; **101:** 15275-15278.

S2. Figg CA, Winegar PH, Hayes OG, Mirkin CA. Controlling the DNA hybridization chain reaction. *J Am Chem Soc* 2020; **142:** 8596-8601.

S3. Li Z, Wang G, Shen Y, Guo N, Ma N DNA-templated magnetic nanoparticle-quantum dot polymers for ultrasensitive capture and detection of circulating tumor cells. *Adv Funct Mater* 2018; **28:** 1707152.

S4. Lin R, Feng Q, Li p *et al.* A hybridization-chain-reaction-based method for amplifying immunosignals. *Nat Methods* 2018; **15:** 275-278.

S5. Wu Z, Liu GQ, Yang XL, Jiang JH. Electrostatic nucleic acid nanoassembly enables hybridization chain reaction in living cells for ultrasensitive mRNA imaging. *J Am Chem Soc* 2015; **137:** 6829-6836.

S6. Tang Y, Zhang XL, Tang LJ, Yu RQ, Jiang JH. In situ imaging of individual mRNA mutation in single cells using ligation-mediated branched hybridization chain reaction (ligation-bHCR). *Anal Chem* 2017; **89:** 3445-3451.

S7. Wang J, Chao J, Liu H *et al.* Clamped hybridization chain reactions for the self-assembly of patterned DNA hydrogels. *Angew Chem Int Ed* 2017; **56:** 2171-2175.

S8. Jia B, Ge J, Ma Y *et al.* Spatially preorganized hybridization chain reaction for the prompt diagnosis of inflammation. *Angew Chem Int Ed* 2025; **64:** e202421022.

S9. Liu X, Mao D, Song Y *et al.* Computer-aided design of reversible hybridization chain reaction (cad-HCR) enables multiplexed single-cell spatial proteomics imaging. *Sci Adv* 2022; **8:** eabk0133.

S10. Li J, Liu S, Sun L *et al.* Amplified visualization of protein-specific glycosylation in zebrafish via proximity-induced hybridization chain reaction. *J Am Chem Soc* 2018; **140:** 16589-16595.

S11. Liu L, Liu JW, Wu H *et al.* Branched hybridization chain reaction circuit for ultrasensitive localizable imaging of mRNA in living cells. *Anal Chem* 2018; **90:** 1502-1505.

S12. Xiao M, Lai W, Wang F *et al.* Programming drug delivery kinetics for active burst release with DNA toehold switches. *J Am Chem Soc* 2019; **141:** 20354-20364.
